# Supplementary material for: Saurodesmus robertsoni Seeley 1891—The oldest Scottish cynodont
Source: PLoS One. 2024 May 29;19(5):e0303973. doi: 10.1371/journal.pone.0303973 (PMC11135747; doi:10.1371/journal.pone.0303973)
Supplement: S1 File — ‘Younginiformes’, Sauropterygia, Choristodera, basal panarchosaurs, ‘Protorosauria’, Allokotosauria, stem Archosauria, Kuehneosauridae. (DOCX) [file pone.0303973.s001.docx]

**Supplementary Material**

***Saurodesmus robertsoni* Seeley 1891 – the oldest Scottish cynodont**

Tomasz Szczygielski^1^*, Marc Johan Van den Brandt^2^, Leandro Gaetano^2, 3^, Dawid Dróżdż^4=^

^1^Institute of Paleobiology, Polish Academy of Sciences, Warsaw, Poland.

^2^ Evolutionary Studies Institute (ESI), University of the Witwatersrand, Johannesburg, PO WITS 2050, South Africa

^3^Instituto de Estudios Andinos “Don Pablo Groeber” (IDEAN, UBA-CONICET), Ciudad Autónoma de Buenos Aires, Argentina

^4^Nalecz Institute of Biocybernetics and Biomedical Engineering, Polish Academy of Sciences, Warsaw, Poland

*Corresponding author

E-mail: [t.szczygielski@twarda.pan.pl](mailto:t.szczygielski@twarda.pan.pl) (T.Sz.)

**Additional comparisons**

**‘Younginiformes’**

In the humeri of the ‘Younginiformes’, e.g., *Youngina capensis* Broom, 1914 [1], the proximal and distal parts of the shaft are rotated at 90 degrees to each other, and have a clear entepicondylar foramen and ectepicondylar groove [2–4]. The femur seems to have a sigmoidal shape and lacks significant proximal and distal expansions [4]. Both humerus and femora are slim compared to NHMUK PV OR 28877 [3,4], therefore it seems unlikely that NHMUK PV OR 28877 might belong to this group.

**Sauropterygia**

In sauropterygians, humeri and femora do not form such well distinguished proximal and distal structures as those in NHMUK PV OR 28877 [5–8]. In nothosaurs, the humerus is thick and bowed, and the femur is straight with slightly expanded proximal and distal end [7]. The humeri and femora of placodonts (e.g., *Placodus gigas* Agassiz, 1833 [9]) have a similar shape in general [8,10]. In plesiosaurs, in which the limbs evolved into fins, both humeri and femora, have a similar morphology with much more expanded distal end in proportion to the proximal end [5,6].

**Choristodera**

In Choristodera, the proximal and distal ends of the humerus are significantly twisted in relation to each other at an angle of about 45 to 60 degrees, and are moderately expanded, compared to NHMUK PV OR 28877. They have a distinct deltopectoral crest, and at least some have the ectepicondylar groove which is lacking in NHMUK R28877 [11–15].

The femora of Choristodera have a straight shaft with a weakly defined femoral head, their distal condyles are much slenderer in comparison to NHMUK PV OR 28877, and they have a distinct internal trochanter [11–15].

Although the Choristodera have a very wide temporal range extending from the Middle Jurassic to Miocene [16], it seems that the morphology of their stylopodia remains conservative. A single species, *Pachystropheus rhaeticus*, is known from the Late Triassic (Rhaetian) of southwest England [14,15,17], but its taxonomical affiliation to Choristodera was disputed, mostly due to its poor preservation [16,18]*.* Nevertheless, the morphology of its stylopodia is consistent with the Chorsitodera [14,15]*.*

**Basal panarchosaurs**

The humeri of basal panarchosaurus, such as *Aenigmastropheus parringtoni* Ezcurra *et al.*, 2014 [19], a late Wuchiapingian stem archosauromorph, differ from NHMUK PV OR 28877 in the presence of the ectepicondylar groove, capitellum and trochlea separated from the epicondyles, and depressed surfaces of the platelike distal expansion [19]. A similar morphology is expected in other basal representatives of the Panarchosauria, refuting interpretation of NHMUK PV OR 28877 as a humerus of a representative of an early-branching steam archosaur lineage.

**‘Protorosauria’**

‘Protorosaurian’ humeri differ from NHMUK PV OR 28877 in weakly expanded proximal ends with a simple, proximally directed articular surface and no separate, laterally directed processes, simpler, plate-like predominantly unilaterally (medially) expanded distal ends of humeri with distinct ectepicondylar notch, as well as sinuous femora with upright trochanters and little to no proximal and distal expansion [2,19–21]. Although mostly known from the Middle Triassic, some representatives of the group survived to the Late Triassic [22].

**Allokotosauria**

Humeri of Allokotosauria are distinguished from NHMUK PV OR 28877 based on their laterally expanded proximal articular surface without separation of the processes, larger torsion, presence of the ectepicondylar groove and distinct fossae on the dorsal and ventral surfaces of the distal expansion, and lack of flattening of the shaft distal the intertubercular fossa and along the entepicodylar edge of the distal end [23–26]. The shape of the distal end and angle between the proximal and distal expansions differ between allokotosaur taxa, *Trilophosaurus buettneri* Case, 1928 [27] (Trilophosauridae) and *Azendohsaurus madagaskarensis* Flynn *et al.*, 2010 [28] (Azendohsauridae) contrast with NHMUK PV OR 28877 in the presence of a prominent entepicondyle and distal articular condyles positioned closely together and separated from the edges of the distal expansion [25,26] while *Malerisarus* spp. (Azendohsauridae) exhibits a much stronger humeral torsion [23,24]. Femora of allokotosaurs resemble NHMUK PV OR 28877 in their rather simple, triangular distal ends with limited proximal reach of the articular surfaces but fifer in very weakly expanded proximal end, ventral direction of the internal trochanter and rounded edges of the distal end [23–26]. Additionally, azendohsaurids have clearly sinuous femora torsion [23,24,26] while *Trilophosaurus buettneri* differs in ventrally protruding tibial condyle and straight tibial edge of the distal end [25].

**Stem Archosauria**

Overall, Archosauriformes more distantly related to archosaurs have their stylopodial bones developed similarly to the more derived representatives of the group: the humeri having transverly expanded proximal and distal ends; deltopectoral crest distinctly parallel to the humeral head; thick deltopectoral crest and internal tuberosity (compared to NHMUK PV OR 28877 if interpreted as a humerus), and having both distal condyles round shaped (one is acute in NHMUK PV OR 28877) with clear entepi- or ectepicondyle foramina (absent in NHMUK PV OR 28877); the femora being distinctly sigmoidal, femoral head oriented medially, and prominent fourth trochanter or internal trochanter in the Proterosuchidae or Erythrosuchidae (all of these features are absent in NHMUK PV OR 28877). Those characteristics are fully applicable to the basal members of the Eucrocopoda (encompassing Avemetatarsalia and Pseudosuchia), including the Euparkeriidae [29–31] and Proterochampsia [32–36], which have their stylopodial bones more or less alike their more derived cousins. Therefore, that excludes the possibility of NHMUK PV OR 28877 belonging to one of those groups.

Little is known about the limbs of the basal eucrocopodan clade, the Doswelliidae. The stylopodial bones are preserved exclusively in *Archeopelta arborensis* Desojo *et al.*, 2011 [37], *Doswellia kaltenbachi* Weems, 1980 [38] (femur), and *Rugarhynchos sixmilensis* (Heckert *et al.*, 2012) [39] (distal femur) [37–43]. However, in the latter two, the bones are too poorly preserved for adequate anatomical comparisons [39,41,43]. In the case of *Archeopelta arborensis*, only the proximal part of the humerus is preserved. Citing the description of the authors, “the humeral head of *Archeopelta* is proximomedially deflected” [37], so in the dorsoventral aspect the lateral edge of transversally expanded proximal part of the shaft is bevelled and slopes diagonally mediolaterally in reference to the straight middle portion of the shaft. This differs from other archosauriforms, in which the lateral edge of the proximal expansion is a straight continuation of the middle portion of the shaft or expands it laterally; the same is true for NHMUK PV OR 28877 (if interpreted as a humerus). The femur of *Archeopelta arborensis* has its general form similar as other archosauriforms [37]. Some analyses [2,44] also recover *Vancleavea campi* Long & Murry, 1995 [42] as a member of the Doswelliidae. However, neither of the stylopodial bones of *Vancleavea campi* reassemble NHMUK PV OR 28877 in general shape. Both the humerus and femur have less developed heads and condyles, and are more gracile in comparison to NHMUK PV OR 28877. The humerus has a well-developed deltopectoral crest and equally-sized and shaped distal condyles. The only similarity is the lack of ectepi- or entepicondylar grooves [45]. The femur of *Vancleavea campi* is sigmoidal with twisted femoral head and condyles. It does not have the fourth or internal trochanter [45], which could be considered a similarity with NHMUK PV OR 28877, if not for the entirely different shape of the shaft.

In the most basal of the Archosauriformes (Proterosuchidae and Erythrosuchidae) the humeri resemble NHMUK PV OR 28877 in their most general shape due to the significant and equal transverse expansion of the proximal and distal parts, as well as their general robust shape. However, NHMUK PV OR 28877 is more gracile and lacks other mentioned features of the humerus [2,46]. The femora of the Proterosuchidae and Erythrosuchidae also slightly differ morphologically from the more advanced archosauriformes. However, they are also different form NHMUK PV OR 28877 in having much less significant proximal and distal expansions compared to the middle portion of the shaft and in having a well-developed internal trochanter [46].

**Kuehneosauridae**

In the gliding Kuehneosauridae, both the humerus and femur are slender and gracile compared to NHMUK PV OR 28877 [47–50]. All known kuehneosaurids are also considerably smaller [48,50]. In the Kuehneosauridae, both the proximal and distal ends of the humeri are expanded, but proportionally not as strongly as in NHMUK PV OR 28877, and the middle portion of the shaft in the Kuehneosauridae is proportionally much longer than in NHMUK PV OR 28877 [47–49]. They also have a distinct entepicondylar foramen which is absent in NHMUK PV OR 28877 [47,48]. The femur in the Kuehneosauridae is a long, straight bone with medially oriented femoral head, and thus not fitting NHMUK PV OR 28877 [47–49]. In addition, long bones of kuehneosaurids have a hollow shaft [50], in contrast to NHMUK PV OR 28877.

**References**

1. Broom R. A new thecodont reptile. Proc Zool Soc London. 1914;1914: 1072–1077.

2. Ezcurra MD. The phylogenetic relationships of basal archosauromorphs, with an emphasis on the systematics of proterosuchian archosauriforms. PeerJ. 2016;4: e1778. doi:10.7717/peerj.1778

3. Smith RMH, Evans SE. New material of *Youngina*: evidence of juvenile aggregation in Permian diapsid reptiles. Palaeontology. 1966;39: 289–303.

4. Gow CE. The Morphology and Relationships of *Youngina capensis* Broom and *Prolacerta broomi* Parrington. Palae ont afr. 1975;18: 89–131.

5. Andrews CW. A descriptive catalogue of the marine reptiles of the Oxford Clay, part 1. London: British Museum (Natural History); 1910. doi:10.4324/9780203035993

6. Robin EO. The evolution of plesiosaur and pliosaur morphotypes in the Plesiosauria (Reptilia: Sauropterygia). Paleobiology. 2002;28: 101–112.

7. Klein N, Eggmaier S, Hagdorn H. The redescription of the holotype of *Nothosaurus mirabilis* (Diapsida, Eosauropterygia)—a historical skeleton from the Muschelkalk (Middle Triassic, Anisian) near Bayreuth (southern Germany). PeerJ. 2022;10: e13818. doi:10.7717/peerj.13818

8. Rieppel O. The genus *Placodus*: systematics, morphology, paleobiogeography, and paleobiology. Fieldiana Geol. 1995;31. doi:10.5962/bhl.title.3301

9. Agassiz L. Recherches sur les poissons fossiles. Tome II, contenant l’histoire de l’orde des ganoïdes. Neuchatel: Petitpierre; 1833.

10. Drevermann F. Die Placodontier. 3. Das skelett von *Placodus gigas* Agassiz im Senckenberg-Museum. Abhandlungen der Senckenbergischen Naturforschenden Gesellschaft. 1933. pp. 321–364.

11. Matsumoto R, Dong L, Wang Y, Evans SE. The first record of a nearly complete choristodere (Reptilia: Diapsida) from the Upper Jurassic of Hebei Province, People’s Republic of China. J Syst Palaeontol. 2019;17: 1031–1048. doi:10.1080/14772019.2018.1494220

12. Lü JC, Kobayashi Y, Li ZG. A new species of *Ikechosaurus* (Reptilia: Choristodera) from the Jiufutang Formation (Early Cretaceous) of Chifeng City, Inner Mongolia. Bull l’Institut R des Sci Nat Belqique, Sci la Terre. 1999;69: 37–47.

13. Gao KQ, Ksepka DT. Osteology and taxonomic revision of *Hyphalosaurus* (Diapsida: Choristodera) from the Lower Cretaceous of Liaoning, China. J Anat. 2008;212: 747–768. doi:10.1111/j.1469-7580.2008.00907.x

14. Storrs GW, Gower DJ. The earliest possible choristodere (Diapsida) and gaps in the fossil record of semi-aquatic reptiles. J Geol Soc London. 1993;150: 1103–1107. doi:10.1144/gsjgs.150.6.1103

15. Storrs GW, Large NF. The diapsid reptile *Pachystropheus rhaeticus*, a probable choristodere from the Rhaetian of Europe. Palaeontology. 1996;39: 323–349.

16. Matsumoto R, Evans SE. Choristoderes and the freshwater assemblages of Laurasia. J Iber Geol. 2010;36: 253–274. doi:10.5209/rev

17. Huene F von. Ein Rhynchocephale aus dem Rhät (*Pachystropheus* n.g.). Neues Jahrb für Mineral Geol und Paläontologie. 1935;74: 441–447.

18. Renesto S. A possible find of *Endennasaurus* (Reptilia Thalattosauria), with a comparison between *Endennasaurus* and *Pachystropheus*. Neues Jahrb fur Geol und Palaontologie - Monatshefte. 2005;2005: 118–128. doi:10.1127/njgpm/2005/2005/118

19. Ezcurra MD, Scheyer TM, Butler RJ. The origin and early evolution of Sauria: Reassessing the Permian Saurian fossil record and the timing of the crocodile-lizard divergence. PLoS One. 2014;9. doi:10.1371/journal.pone.0089165

20. Peyer B. Die Triasfauna der Tessiner Kalkalpen. XII. *Macrocnemus bassanii* Nopcsa. Abhandlungen der Schweizerischen Palaeontol Gesellschaft. 1937;59: 1–140.

21. Wild R. Die Triasfauna der Tessiner Kalkalpen. XXIII. *Tanystropheus longobardicus* (Bassani) (Neue Ergebnisse). Schweizerische Paläontologische Abhandlungen. 1973;95: 1–162.

22. Pritchard AC, Turner AH, Nesbitt SJ, Irmis RB, Smith ND. Late Triassic tanystropheids (Reptilia, Archosauromorpha) from northern New Mexico (Petrified Forest Member, Chinle Formation) and the biogeography, functional morphology, and evolution of Tanystropheidae. J Vertebr Paleontol. 2015;35: 37–41. doi:10.1080/02724634.2014.911186

23. Nesbitt SJ, Stocker MR, Ezcurra MD, Fraser NC, Heckert AB, Parker WG, et al. Widespread azendohsaurids (Archosauromorpha, Allokotosauria) from the Late Triassic of western USA and India. Pap Palaeontol. 2022;8: e1413. doi:10.1002/spp2.1413

24. Chatterjee S. *Malerisaurus langstoni*, a new diapsid reptile from the Triassic of Texas. J Vertebr Paleontol. 1986;6: 297–312. doi:10.1080/02724634.1986.10011627

25. Spielmann JA, Lucas SG, Rinehart LF, Heckert AB. The Late Triassic archosauromorph *Trilophosaurus*. New Mex Museum Nat Hist Sci Bull. 2008;43: 1–177.

26. Nesbitt SJ, Flynn JJ, Pritchard AC, Parrish JM, Ranivoharimanana L, Wyss AR. Postcranial osteology of *Azendohsaurus madagaskarensis* (?Middle to Upper Triassic, Isalo Group, Madagascar) and its systematic position among stem archosaur reptiles. Bull Am Museum Nat Hist. 2015;398: 1–126. doi:10.1206/amnb-899-00-1-126.1

27. Case EC. A cotylosaur from the Upper Triassic of western Texas. J Washingt Acad Sci. 1928;18: 177–178.

28. Flynn JJ, Nesbitt SJ, Michael Parrish J, Ranivoharimanana L, Wyss AR. A new species of *Azendohsaurus* (Diapsida: Archosauromorpha) from the Triassic Isalo Group of southwestern Madagascar: Cranium and mandible. Palaeontology. 2010;53: 669–688. doi:10.1111/j.1475-4983.2010.00954.x

29. Sookias RB, Butler RJ. Euparkeriidae. Geol Soc Spec Publ. 2013;379: 35–48. doi:10.1144/SP379.6

30. Demuth OE, Rayfield EJ, Hutchinson JR. 3D hindlimb joint mobility of the stem-archosaur Euparkeria capensis with implications for postural evolution within Archosauria. Sci Rep. 2020;10: 1–14. doi:10.1038/s41598-020-70175-y

31. Borsuk-Białynicka M, Sennikov AG. Archosauriform postcranial remains from the early Triassic karst deposits of southern Poland. Palaeontol Pol. 2009;65: 283–328.

32. Trotteyn MJ, Arcucci AB, Raugust T. Proterochampsia: An endemic archosauriform clade from South America. Geol Soc Spec Publ. 2013;379: 59–90. doi:10.1144/SP379.23

33. Müller RT, Garcia MS, Fonseca A de O. A new proterochampsid (Archosauriformes: Proterochampsia) from the Late Triassic of southern Brazil and the emergence of archosaurian hind limb traits. J Syst Palaeontol. 2022;20: 1–19. doi:10.1080/14772019.2022.2128913

34. Trotteyn MJ. Material Postcraneano de *Proterochampsa barrionuevoi* Reig 1959 (Diapsida: Archosauriformes) del Triásico Superior del Centro-oeste de Argentina. Ameghiniana. 2011;48: 424–446. doi:10.5710/AMGH.v48i4(351)CITATIONS

35. Trotteyn MJ, Martínez RN, Alcober OA. A new proterochampsid *Chanaresuchus ischigualastensis*  (Diapsida, Archosauriformes) in the early Late Triassic Ischigualasto Formation, Argentina. J Vertebr Paleontol. 2012;32: 485–489. doi:10.1080/02724634.2012.645975

36. Arcucci AB. Un nuevo Proterochampsidae (Reptilia–Archosauriformes) de la fauna local de Los Chanares (Triasico Medio), La Rioja, Argentina. Ameghiniana. 1990;27: 365–378.

37. Desojo JB, Ezcurra MD, Schultz CL. An unusual new archosauriform from the Middle-Late Triassic of southern Brazil and the monophyly of Doswelliidae. Zool J Linn Soc. 2011;161: 839–871. doi:10.1111/j.1096-3642.2010.00655.x

38. Weems RE. An unusual uewly discovered archosaur from the Upper Triassic of Virginia, U.S.A. Trans Am Philos Soc. 1980;70: 1. doi:10.2307/1006472

39. Heckert AB, Lucas SG, Spielmann JA. A new species of the enigmatic archosauromorph *Doswellia* from the Upper Triassic Bluewater Creek Formation, New Mexico, USA. Palaeontology. 2012;55: 1333–1348. doi:10.1111/j.1475-4983.2012.01200.x

40. Sues HD, Desojo JB, Ezcurra MD. Doswelliidae: A clade of unusual armoured archosauriforms from the Middle and Late Triassic. Geol Soc Spec Publ. 2013;379: 49–58. doi:10.1144/SP379.13

41. Dilkes D, Sues HD. Redescription and phylogenetic relationships of *Doswellia kaltenbachi* (diapsida: Archosauriformes) from the Upper Triassic of Virginia. J Vertebr Paleontol. 2009;29: 58–79. doi:10.1080/02724634.2009.10010362

42. Long RA, Murry PA. Late Triassic (Carnian and Norian) tetrapods from the southwestern United States. New Mex Museum Nat Hist Sci Bull. 1995;4: 1–254.

43. Wynd BM, Nesbitt SJ, Stocker MR, Heckert AB. A detailed description of *Rugarhynchos sixmilensis*, gen. et comb. nov. (Archosauriformes, Proterochampsia), and cranial convergence in snout elongation across stem and crown archosaurs. J Vertebr Paleontol. 2019;39: e1748042. doi:10.1080/02724634.2019.1748042

44. Sookias RB. The relationships of the Euparkeriidae and the rise of Archosauria. R Soc Open Sci. 2016;3. doi:10.1098/rsos.150674

45. Nesbitt SJ, Stocker MR, Small BJ, Downs A. The osteology and relationships of *Vancleavea campi* (Reptilia: Archosauriformes). Zool J Linn Soc. 2009;157: 814–864. doi:10.1111/j.1096-3642.2009.00530.x

46. Ezcurra MD, Butler RJ, Gower DJ. “Proterosuchia”: The origin and early history of archosauriformes. Geol Soc Spec Publ. 2013;379: 9–33. doi:10.1144/SP379.11

47. Colbert EH. A gliding reptile from the Triassic of New Jersey. Am Museum Novit. 1966; 1–23.

48. Colbert EH. The Triassic Gliding Reptile: *Icarosaurus*. Bull Am Museum Nat Hist. 1970;143: 85–142.

49. Robinson PL. Gliding lizards from the Upper Keuper of Great Britain. Proc Geol Soc London. 1962;1601: 137–146.

50. Stein K, Palmer C, Gill PG, Benton MJ. The aerodynamics of the British Late Triassic Kuehneosauridae. Palaeontology. 2008;51: 967–981. doi:10.1111/j.1475-4983.2008.00783.x
