# Supplementary material for: Saurodesmus robertsoni Seeley 1891—The oldest Scottish cynodont
Source: PLoS One. 2024 May 29;19(5):e0303973. doi: 10.1371/journal.pone.0303973 (PMC11135747; doi:10.1371/journal.pone.0303973)

PLOS ONE

Saurodesmus robertsoni Seeley 1891 – the oldest Scottish cynodont

--Manuscript Draft--

**Manuscript Number:**	PONE-D-23-36210
**Article Type:**	Research Article
**Full Title:**	Saurodesmus robertsoni Seeley 1891 – the oldest Scottish cynodont
**Short Title:**	Reassessment of Saurodesmus robertsoni
**Corresponding Author:**	Tomasz Szczygielski, Ph.D.<br>Institute of Paleobiology Polish Academy of Sciences: Instytut Paleobiologii im Romana Kozlowskiego Polskiej Akademii Nauk<br>Warsaw, POLAND
**Keywords:**	Paleontology; Triassic; Norian; Cynodontia; cynodont; Synapsida; Tritylodontidae; Saurodesmus; Pantestudinata; Testudinata; turtle; Crocodylia; crocodile; Scotland; Great Britain; United Kingdom; Europe, femur; bone; fossil
**Abstract:**	Predating Darwin's theory of evolution, the holotype of Saurodesmus robertsoni is a long-standing enigma. Found at the beginning of 1840s, the specimen is a damaged stylopodial bone over decades variably assigned to turtles, archosaurs, parareptiles, or synapsids, and currently nearly forgotten. We redescribe and re-assess that curious specimen as a valid taxon of a derived cynodont (?Tritylodontidae). This makes Saurodesmus robertsoni the first Triassic cynodont from Scotland and, possibly, one of the earliest representatives of tritylodontids worldwide. Moreover, it highlights the need for revisiting historical problematic specimens, the identification of which could have been previously hampered by the lack of adequate comparative materials in the past.
**Order of Authors:**	Tomasz Szczygielski, Ph.D.
	Marc Johan Van den Brandt, Ph.D.
	Leandro Gaetano, Ph.D.
	Dawid Dróżdż, Ph.D.
**Additional Information:**	
**Question**	**Response**
**Financial Disclosure**<br><br>Enter a financial disclosure statement that describes the sources of funding for the work included in this submission. Review the submission guidelines for detailed requirements. View published research articles from *PLOS ONE* for specific examples.<br><br>This statement is required for submission and **will appear in the published article** if the submission is accepted. Please make sure it is accurate.	Yes

Powered by Editorial Manager® and ProduXion Manager® from Aries Systems Corporation

**Funded studies**<br>Enter a statement with the following details:<br>• Initials of the authors who received each award<br>• Grant numbers awarded to each author<br>• The full name of each funder<br>• URL of each funder website<br>• Did the sponsors or funders play any role in the study design, data collection and analysis, decision to publish, or preparation of the manuscript?<br><br>Did you receive funding for this work?	
Please add funding details.<br>as follow-up to "**Financial Disclosure**<br><br>Enter a financial disclosure statement that describes the sources of funding for the work included in this submission. Review the submission guidelines for detailed requirements. View published research articles from *PLOS ONE* for specific examples.<br><br>This statement is required for submission and **will appear in the published article** if the submission is accepted. Please make sure it is accurate.<br><br>**Funded studies**<br>Enter a statement with the following details:<br>• Initials of the authors who received each award<br>• Grant numbers awarded to each author<br>• The full name of each funder<br>• URL of each funder website<br>• Did the sponsors or funders play any role in the study design, data collection and analysis, decision to publish, or preparation of the manuscript?<br><br>Did you receive funding for this work?"	The study was supported by the National Science Centre, Poland (Narodowe Centrum Nauki, https://www.ncn.gov.pl/en) grant no. 2020/39/B/NZ8/01074 awarded to T. Sz. and GENUS (DSI-NRF Centre of Excellence in Palaeosciences, UID 86073, https://www.genus.africa) and the Millenium Trust South Africa (no number, https://www.mtrust.co.za) awarded to M. J.V.d.B. The funders did not play any role in the study design, data collection and analysis, decision to publish, or preparation of the manuscript.
Please select the country of your main research funder (please select carefully as in some cases this is used in fee calculation).	POLAND - PL

Powered by Editorial Manager® and ProduXion Manager® from Aries Systems Corporation

as follow-up to "**Financial Disclosure**<br><br>Enter a financial disclosure statement that describes the sources of funding for the work included in this submission. Review the submission guidelines for detailed requirements. View published research articles from *PLOS ONE* for specific examples.<br><br>This statement is required for submission and **will appear in the published article** if the submission is accepted. Please make sure it is accurate.<br><br>**Funded studies**<br>Enter a statement with the following details:<br>• Initials of the authors who received each award<br>• Grant numbers awarded to each author<br>• The full name of each funder<br>• URL of each funder website<br>• Did the sponsors or funders play any role in the study design, data collection and analysis, decision to publish, or preparation of the manuscript?<br><br>Did you receive funding for this work?"	
**Competing Interests**<br><br>Use the instructions below to enter a competing interest statement for this submission. On behalf of all authors, disclose any competing interests that could be perceived to bias this work—acknowledging all financial support and any other relevant financial or non-financial competing interests.<br><br>This statement is required for submission and **will appear in the published article** if the submission is accepted. Please make sure it is accurate and that any funding sources listed in your Funding Information later in the submission form are also declared in your Financial Disclosure statement.	The authors have declared that no competing interests exist.

View published research articles from *PLOS ONE* for specific examples.<br><br>**NO authors have competing interests**<br><br>Enter: *The authors have declared that no competing interests exist.*<br><br>**Authors with competing interests**<br><br>Enter competing interest details beginning with this statement:<br><br>*I have read the journal's policy and the authors of this manuscript have the following competing interests: [insert competing interests here]*<br><br>* typeset	
**Ethics Statement**<br><br>Enter an ethics statement for this submission. This statement is required if the study involved:<br><br>• Human participants<br>• Human specimens or tissue<br>• Vertebrate animals or cephalopods<br>• Vertebrate embryos or tissues<br>• Field research<br><br>Write "N/A" if the submission does not require an ethics statement.<br><br>General guidance is provided below. Consult the submission guidelines for detailed instructions. **Make sure that all information entered here is included in the Methods section of the manuscript.**	N/A

Powered by Editorial Manager® and ProduXion Manager® from Aries Systems Corporation

**Format for specific study types**<br><br>**Human Subject Research (involving human participants and/or tissue)**<br>• Give the name of the institutional review board or ethics committee that approved the study<br>• Include the approval number and/or a statement indicating approval of this research<br>• Indicate the form of consent obtained (written/oral) or the reason that consent was not obtained (e.g. the data were analyzed anonymously)<br><br>**Animal Research (involving vertebrate animals, embryos or tissues)**<br>• Provide the name of the Institutional Animal Care and Use Committee (IACUC) or other relevant ethics board that reviewed the study protocol, and indicate whether they approved this research or granted a formal waiver of ethical approval<br>• Include an approval number if one was obtained<br>• If the study involved *non-human primates*, add *additional details* about animal welfare and steps taken to ameliorate suffering<br>• If anesthesia, euthanasia, or any kind of animal sacrifice is part of the study, include briefly which substances and/or methods were applied<br><br>**Field Research**<br><br>Include the following details if this study involves the collection of plant, animal, or other materials from a natural setting:<br>• Field permit number<br>• Name of the institution or relevant body that granted permission	
**Data Availability**<br><br>Authors are required to make all data underlying the findings described fully available, without restriction, and from the time of publication. PLOS allows rare exceptions to address legal and ethical concerns. See the PLOS Data Policy and FAQ for detailed information.	Yes - all data are fully available without restriction

Powered by Editorial Manager® and ProduXion Manager® from Aries Systems Corporation

A Data Availability Statement describing where the data can be found is required at submission. Your answers to this question constitute the Data Availability Statement and **will be published in the article**, if accepted.<br><br>**Important:** Stating 'data available on request from the author' is not sufficient. If your data are only available upon request, select 'No' for the first question and explain your exceptional situation in the text box.<br><br>Do the authors confirm that all data underlying the findings described in their manuscript are fully available without restriction?	
**Describe where the data may be found in full sentences. If you are copying our sample text, replace any instances of XXX with the appropriate details.**<br><br>• If the data are **held or will be held in a public repository**, include URLs, accession numbers or DOIs. If this information will only be available after acceptance, indicate this by ticking the box below. For example: *All XXX files are available from the XXX database (accession number(s) XXX, XXX.).*<br>• If the data are all contained **within the manuscript and/or Supporting Information files**, enter the following: *All relevant data are within the manuscript and its Supporting Information files.*<br>• If neither of these applies but you are able to provide **details of access elsewhere**, with or without limitations, please do so. For example:<br><br>*Data cannot be shared publicly because of [XXX]. Data are available from the XXX Institutional Data Access / Ethics Committee (contact via XXX) for researchers who meet the criteria for access to confidential data.*<br><br>*The data underlying the results presented in the study are available from (include the name of the third party*	All relevant data are within the manuscript. The specimens described belong to their respective collections held at scientific institutions and museums (see Institutional abbreviations) and are available for study.

and contact information or URL).<br>• This text is appropriate if the data are owned by a third party and authors do not have permission to share the data.<br><br>* typeset	
Additional data availability information:	

Powered by Editorial Manager® and ProduXion Manager® from Aries Systems Corporation

Manuscript
Click here to access/download;Manuscript;Saurodesmus 2023 11 01 Submission.docx

Saurodesmus robertsoni Seeley 1891 – the oldest Scottish cynodont

Tomasz Szczygielski[1]*, Marc Johan Van den Brandt[2], Leandro Gaetano[2, 3], Dawid Dróżdż[4]

[1]Institute of Paleobiology, Polish Academy of Sciences, Warsaw, Poland.

[2] Evolutionary Studies Institute (ESI), University of the Witwatersrand, Johannesburg, PO WITS 2050, South Africa

[3]Instituto de Estudios Andinos "Don Pablo Groeber" (IDEAN, UBA-CONICET), Ciudad Autónoma de Buenos Aires, Argentina

[4]Nalecz Institute of Biocybernetics and Biomedical Engineering, Polish Academy of Sciences, Warsaw, Poland

*Corresponding author

E-mail: t.szczygielski@twarda.pan.pl (T.Sz.)

Abstract

Predating Darwin's theory of evolution, the holotype of *Saurodesmus robertsoni* is a long-standing enigma. Found at the beginning of 1840s, the specimen is a damaged stylopodial bone over decades variably assigned to turtles, archosaurs, parareptiles, or synapsids, and currently nearly forgotten. We redescribe and re-assess that curious specimen as a valid taxon of a derived cynodont (?Tritylodontidae). This makes *Saurodesmus robertsoni* the first Triassic cynodont from Scotland and, possibly, one of the earliest representatives of tritylodontids worldwide. Moreover, it highlights the need for revisiting historical problematic specimens, the identification of which could have been previously hampered by the lack of adequate comparative materials in the past.

Data availability: All relevant data are within the manuscript. The specimens described belong to their respective collections held at scientific institutions and museums (see Institutional abbreviations) and are available for study.

Funding: The study was supported by the National Science Centre, Poland (Narodowe Centrum Nauki, https://www.ncn.gov.pl/en) grant no. 2020/39/B/NZ8/01074 awarded to T. Sz. and GENUS (DSI-NRF Centre of Excellence in Palaeosciences, UID 86073, https://www.genus.africa) and the Millenium Trust South Africa (no number, https://www.mtrust.co.za) awarded to M. J.V.d.B.

Competing interests: The authors have declared no competing interests exist.

Introduction

Saurodesmus robertsoni Seeley, 1891 [2] is an enigmatic taxon based on as single problematic limb bone from the Late Triassic of Linksfield, Elgin, Scotland (NHMUK PV OR 28877). Despite its long, relatively rich and regular record of mentions in the scientific literature, its systematic history is exceptionally convoluted. The first, never published illustration of the specimen, a watercolour by

Jonathan Stiven (~1799–1872), was created in 1840 (Fig 1). Based on the associated caption, NHMUK PV OR 28877 was found by Alexander Robertson. The watercolour is annotated in pencil with an apparently erroneous reference to one of two possible papers by Malcolmson [3,4], neither of which, however, clearly refers to this particular bone (see Material and methods).

Fig 1. *Saurodersmus robertsoni* NHMUK PV OR 28877, right femur and associated rock fragment before they were separated. "Drawn by J. Stiven, from a specimen found at Linksfield by Alex^{r}. Robertson Esqr. Inverugie F. G. S. Elgin 1840". Watercolour. Note the bone is figured upside-down (proximal end towards the bottom of the page). Reproduced by permission of the Geological Society of London.

The specimen was first officially announced by Owen [5] in his report on British fossil reptiles for the year 1841 as a turtle femur resembling (but not identical with) that of *Trionyx* spp. The first published illustration of the specimen, also captioned as a turtle femur, was published by Duff ([6], pl. V, fig. 10). Anderson and Anderson ([7], footnote by A. Robertson) and Pictet [8] listed it as a femur of *Trionyx* sp. After acquisition by the British Museum (Natural History) in 1854, it was catalogued by W. Davies as a "humerus? of a Chelonian?" [1] (also in the collection inventory book). Jones [9] mentioned it as a femur of a "chelonian animal" and Maack [10] questioned its turtle affinities, but Judd [11] repeated the earlier identification as a femur of *Trionyx* sp. Seeley [12] suggested a dinosaurian affinity for the specimen. Quenstedt [13] denied its identification as *Trionyx* sp. which he attributed to Owen [5], despite the latter only mentioned the resemblance of the specimen to that genus, and probably even as a turtle at all, deeming it "insufficient". Lydekker [14] mentioned it as a right humerus or femur of a turtle referable to '*Chelytherium obscurum*' Meyer, 1863 [15] (synonym of *Proterochersis robusta* Fraas, 1913 [16] (see [17]; application for conservation of the junior name submitted [18]) from the Norian of Germany or a related taxon but gave no justification and noted that it is different from humeri and femora of any other known turtle. Woodward and Sherborn [19],

however, listed it again as a possible femur of *Trionyx* sp. A year later, Seeley [1,2] revised his earlier interpretation, hesitantly identifying the specimen as a crocodilian right humerus, and was the first to describe the specimen in detail and assign a generic and specific name to it. He also provided more detailed figures, for the first time revealing the ventral surface of the bone, which was embedded in the rock matrix until the preparatory work performed in 1889 [1]. The crocodilian interpretation of the specimen was straight away categorically rejected by Richard Lydekker in the discussion published alongside the paper [1], who suggested once again a testudinate or rhynchocephalian affinity, and the dubious taxonomical character of the specimen was stressed even in the short synopsis of the paper published the same year in American Geologist [20]. Soon after, Seeley observed some similarity of the proximal end of NHMUK PV OR 28877 to pareiasaurian femora [21] and of the distal end to dinocephalian femora [22–24], allowing the possibility that *Saurodesmus robertsoni* may be an anomodont (note that the group at the time differed in its taxonomic definition from the current understanding of the Anomodontia, as defined by Kammerer & Angielczyk [25]). The perceived similarity with dinocephalians was later reiterated by Seeley [26] in regard to the whole bone. Finally, Seeley [27], revised his interpretation yet again, noticing the resemblance of the proximal part of the femur to the newly established cynodont *Cynognathus crateronotus* Seeley, 1895 [27]; however, he did not specify whether he considered this similarity systematically important. Huene [28] classified the specimen as a right crurotarsan (his Parasuchia, but in contrast to the modern understanding of the group, including a mix of phytosaurs and aetosaurs) humerus, but noted [28] that it may represent his Lycosauria (a group within Theriodontia in Anomodontia including several other problematic taxa). Subsequently, he mentioned the specimen as a crocodile humerus [29]. Nopcsa [30], on the contrary, refuted the identification of the bone as an archosaurian humerus based on the morphology of the proximal end and as a non-archosaurian reptilian humerus based on the lack of the ectepicondylar foramen. Instead, he considered it to be a pleurodiran turtle femur. Curiously, NHMUK PV OR 28877 was listed by Hummel [31] in the fossil trionychian part of "Fossilium catalogus" as a femur of an unknown form (unspecified whether turtle or not) under "*Trionyx* sp. Owen 1842" but without any reference to *Saurodesmus robertsoni*. Attribution of *Saurodesmus robertsoni* to the Triassic turtle family Proterochersidae was first suggested by Nopcsa [32], and later in the second English translation

of Zittel's "Grundzüge der Paläontologie" [33] (interpreting the bone as a femur) and by Romer [34] (interestingly, the taxon was later omitted by Romer [35]). In all these cases there was justification even though all three works treated proterochersids as amphichelydians ("primitive turtles"; e.g. [36]) not belonging to the Pleurodira (in contrast to initial interpretations; e.g. [16]). A similar approach also led Bergounioux [37] and Huene [38] to declare *Saurodesmus robertsoni* as a possible femur of a proterochersid turtle (this time classifying Proterochersidae as a family of the Pleurodira). Later, the specimen was listed as a marine [sic!] primitive turtle by Swinnerton ([39] and subsequent reprints), as a possible turtle by Kuhn [40], as a right crocodilian humerus by Ingles and Sawyer [41], and as a crocodile by Benton and Walker [42]. Taylor and Cruickshank [43], based on their personal communication with G. W. Storrs, and Storrs [44] dubbed it a reptilian humerus of unknown affinity but not crocodilian. Its turtle relationship was recently questioned by Joyce [45] and this conclusion was upheld by Szczygielski [17] but no serious effort was taken to revise the specimen in recent decades. Adding to the complicated systematic history of that troublesome taxon, "*Saurodesmus*" is also an occasional misspelling of *Staurodesmus* Teiling, 1948 [46] – a genus of charophyte algae [47–51]. The aim of this work is to redescribe *Saurodesmus robertsoni*, provide new documentation of the only known specimen, and identify it taking into account new discoveries of similar patterned long bones in other amniotes made since its description.

Institutional abbreviations

BP (ESI), Evolutionary Studies Institute (formerly Bernard Price Institute), University of the Witwatersrand, Johannesburg, South Africa; CXPM, Chuxiong Prefectural Museum, Chuxiong, China; FMNH CUP, Catholic University of Peking collection of the Field Museum of Natural History, Chicago, USA; IVPP, Institute of Vertebrate Paleontology and Paleoanthropology, Chinese Academy of Sciences, Beijing, China; MB, Museum für Naturkunde, Berlin, Germany; MCZ, Museum of Comparative Zoology, Harvard University, Cambridge, USA; NHMUK, The Natural History Museum, London, United Kingdom; NMQR, National Museum, Bloemfontein, South Africa; PIN,

Palaeontological Institute, Russian Academy of Sciences, Moscow, Russia; PULR, Universidad Nacional de La Rioja, La Rioja, Argentina; SMNS, Staatliches Museum für Naturkunde Stuttgart, Stuttgart, Germany; UF H, Florida Museum of Natural History, Division of Herpetology, Gainesville, USA; UFRGS-PV-1043-T, Departamento de Paleontologia e Estratigrafia, Instituto de Geociências, Universidade Federal do Rio Grande do Sul, Porto Alegre, Brazil; ZPAL, Institute of Paleobiology, Polish Academy of Sciences, Warsaw, Poland.

Material and methods

NHMUK PV OR 28877 comes from a glacial estuarine shale erratic of Rhaetian (Late Triassic) age from Linksfield, Elgin [1,11,42,44]. It belonged to the private collection of Alexander Robertson from Inverugie, Aberdeenshire, Scotland [1,5,6] and was purchased by the Natural History Museum, UK, London, in 1854 [1].

The locality of Linksfield first identified by Gordon [52] was initially considered Early Jurassic (Lias) [5,52], later an equivalent to the Late Jurassic–Early Cretaceous Purbeck or Wealden strata [3,4,6,7], and eventually dated to Rhaetian [9,53–55]. Geological studies in the locality were performed by Brickenden [56] and details summarized by Judd [11]. Beside *Saurodesmus robertsoni*, the locality has also yielded the remains of plants, invertebrates, plesiosaurs, and fish [1,5,58,59,6,7,11,19,42–44,57].

The oldest known illustration of the specimen by Jonathan Stiven (Fig 1) states that it was found by Alexander Robertson and includes a note in pencil: “Malcolmson (Wealden Elgin) 1838 [or 1839 – not entirely clear] Proc Vol 3 p. 141”. This seems to be a mix-up of two references: a report on supposed Wealden strata in Linksfield published by Malcolmson in 1838 (Proceedings of the Geological Society of London, volume 2, pp. 667–669) [3] and an abstract of his presentation from 1839 (Proceedings of the Geological Society of London, volume 3, pp. 141–144) [4] concerned with Palaeozoic localities from the area and only mentioning “the Purbeck beds at Linksfield” in passing.

However, neither of these papers mentions NHMUK PV OR 28877, at least not in a clear way. Malcolmson [3] declared that “the Rev. G. Gordon has found a Saurian bone” in Linksfield, but that does not fit the caption on the illustration attributing the find to Robertson. Note that no other sources explicitly state who found the bone (Owen [5], Duff [6], and Seeley [1] only wrote that it had been in the possession of Robertson, not that he was the original discoverer) nor when it was found (Stiven’s illustration is captioned “Elgin 1840” but the layout of the text suggests that this is the date when the watercolour was produced, not necessarily the date of the finding). Therefore, there is a slight chance that this is the same bone that was indeed found by Gordon and, e.g., given to Robertson, that they found it together, or that the attribution of the find to either Gordon (by Malcolmson [3]) or Robertson (by Stiven) was in error. Alternatively, this may be a separate specimen – in such a case, however, Gordon’s specimen would either have to be a plesiosaur neural arch figured by Duff [6] (pl. V, fig. 2; no owner named) or it probably never resurfaced in the scientific literature; although the Linksfield strata contain plesiosaurs, the only other published bone specimens were vertebrae found by John Martin and Charles Moore [6,43,54,58]. Publication of the full version of Malcolmson’s [4] communication was postponed, as it was decided by the Council of the Geological Society that fish material should be first studied and described by Louis Agassiz, and its final rendition (still mentioning Linksfield only in passing and not referring to NHMUK PV OR 28877) was assembled fifteen years after Malcolmson’s death from recovered manuscript and geological sections [60]. If the intended use of Stiven’s watercolour of NHMUK PV OR 28877 was to accompany Malcolmson’s work in any way, it was never fulfilled.

NHMUK PV OR 28877 and its associated natural mould as well as the humeri of *Stagonolepis olenkae* Sulej, 2010 [61] ZPAL Ab. III/1175 and *Parasuchus* sp. ZPAL Ab. III/1994, femora of *Bienotherium yunnanense* Young, 1940 [62] FMNH CUP 2285, *Bradysaurus baini* (Seeley, 1892) [21] NHMUK PV R 4066, *Cynognathus crateronotus* NMQR 1206, Cynognathia indet. (*Cynognathus* sp. or *Diademodon* sp.) BP/1/1675, *Langbergia modisei* Abdala, Neveling, & Welman, 2006 [63] NMQR 3255, *Moschorhinus kitchingi* Broom, 1920 [64] NMGR 3939, and *Tritylodon longaevus* Owen, 1884 [65] BP/1/5089 and BP/1/4783, and humerus and femur of *Palaeochersis talampayensis*

Rougier, Fuente, & Arcucci, 1995 [66] PULR 068, were digitized using photogrammetry by photographing each piece and generating sparse clouds, depth maps, and mesh in Agisoft Metashape 1.8.4.14856 Pro. Humeri and femora of *Proterochersis porebensis* Szczygielski & Sulej, 2016 [67] ZPAL V. 39/50, ZPAL V. 39/156, and ZPAL V.39/432, *Proganochelys quenstedti* Baur, 1887 [68] SMNS 16980 and SMNS 17203, *Labidosaurus hamatus* (Cope, 1895) [69] MB.R.4433, *Ornithorhynchus anatinus* (Shaw & Nodder, 1799) [70] ZPAL z.p. M. 12/13, and *Silesaurus opolensis* Dzik, 2003 [71] ZPAL Ab. III/452 were digitized using the Shining 3D EinScan Pro 2X 3D scanner fixed on a tripod with Ein-Turntable (alignment based on features), and EXScan Pro 3.2.0.2–3.7.0.3 software. The number of turntable steps was varied, chosen depending on the specimen. 3D models of the humerus and femur of *Trionyx triunguis* (Forskål, 1775) [72] UF H65522 (UF:Herp:65522; ark:/87602/m4/454288 and ark:/87602/m4/454285) and femur of *Chelus fimbriatus* [73] UF H85199 (UF:Herp:85199; ark:/87602/m4/450855) were generated based on CT-scans performed on General Electric phoenix v|tome|x m 240 in the UF Nanoscale Research Facility (University of Florida), available on MorhoSource (project "Macroevolutionary patterns of shape evolution in turtles", NKC 1.0). The 3D model of the humerus of *Crocodylus porosus* Schneider, 1801 [74] (Laboratory of Stephen Wroe, l-sw:xcb:Cp4; ark:/87602/m4/M114012) was generated based on CT-scans performed on Siemens Somatom Definition AS+ in I-MED Radiology (I-MED Radiology Armidale) and is available on MorhoSource [75] (CC BY 4.0). For figures, snapshots of the 3D models were captured in MeshLab 2021.10 [76] in orthographic view with Radiance Scaling (Lambertian and Lit Sphere) shader [77] enabled to enhance the surface detail and lighting. Photographs of *Brasilodon quadrangularis* Bonaparte et al., 2003 [78] UFRGS-PV-1043-T were modified from Guignard *et al.* [79] – CC BY 4.0.

Comparative descriptions encompassing representatives of numerous amniote groups, frequently differing in stance and degree of torsion of stylopodial bones, especially when dealing with problematic specimens of uncertain affinity, unavoidably leads to some problems concerning directional terminology as homologous structures and surfaces may be, e.g., variably directed posteriorly or ventrally, medially or anteriorly, etc. To avoid confusion, we prioritize nomenclature

typically employed for each group and in the cited references. That means that the specimen in question needs to be considered accordingly. The terminology for cynodont femora follows the convention employed by Jenkins [80] and Guignard *et al.* [79]. Comparisons with cynodont taxa are based on personal observation of the specimens, photographs, 3D models, and previously published information [79,80,89–98,81,99–105,82–88] (see Table 1).

Table 1. Cynodont taxa allowing morphological comparisons with NHMUK PV OR 28877 and relevant literature references.

**Clade**	**Taxon**	**References**
early cynodonts	*Bolotridon frerensis* (Seeley, 1895) [27]	[27]
	Galesaurus planiceps Owen, 1860 [106]	[102,107,108]
	Procynosuchus delaharpeae Broom, 1937 [109]	[96]
	Thrinaxodon liorhinus Seeley, 1894 [110]	[80]
Cynognathia	*Andescynodon mendozensis* Bonaparte, 1969 [111]	[84,95]
	Boreogomphodon jeffersoni Sues & Olsen, 1990 [112]	[95,113]
	Cricodon metabolus Crompton, 1954 [89]	[89]
	Cynognathus crateronotus Seeley, 1895 [27]	[27]
	Diademodon tetragonus Seeley, 1894 [114]	[80,88,115]
	Exaeretodon argentinus (Cabrera, 1943) [116]	[95,117]
	Luangwa drysdalli Brink, 1963 [118]	[95,104]
	Massetognathus ochagaviae Barberena, 1981 [119]	[103]
	Massetognathus pascuali Romer, 1967 [120]	[92,95]
	Pascualgnathus polanskii Bonaparte, 1966 [121]	[95]
	Scalenodon angustifrons (Parrington, 1946) [122]	[95]
	Traversodon stahleckeri Huene, 1936 [100]	[95,100]
	Menadon besairiei Flynn et al., 2000 [123]	[99]

	Santacruzodon hopsoni Abdala & Ribeiro, 2003 [124]	[99]
Probainognathia	*Aleodon cromptoni* Martinelli et al., 2017 [125]	[126]
	Brasilodon quadrangularis Bonaparte et al., 2003 [78]	[79,85]
	Chiniquodon omaruruensis Mocke, Gaetano, & Abdala, 2020 [127]	[127]
	Chiniquodon theotonicus Huene, 1936 [100]	[93,94,100,126]
	Irajatherium hernandezi Martinelli et al., 2005 [82]	[82,91]
	Probainognathus jenseni Romer, 1970 [128]	[93]
	Prozostrodon brasiliensis (Barbarena, Bonaparte, & Teixeira, 1987) [129]	[87]
	Therioherpeton cargini Bonaparte & Barberena, 1975 [130]	[131]
	Trucidocynodon riograndensis De Oliveira, Soares, & Schultz, 2010 [105]	[105,132]
	Sinoconodon rigneyi Patterson & Olson, 1961 [133]	[134]
Tritylodontidae	*Bienotherium yunnanense* Young, 1940 [62]	[98]
	Bienotheroides wansienensis Young, 1982 [135]	[97]
	Oligokyphus major Kühne, 1956 [90]	[90]
	Tritylodon longaevus Owen, 1884 [65]	[86]
	Tritylodontidae indet. CXPM C2019 2A235	[136]
	Tritylodontidae indet. MCZ 8838 (?*Kayentatherium wellesi* Kermack, 1982 [137])	[83]
Mammaliaformes	*Eozostrodon parvus* Parrington, 1941 [138]	[81,134]
	Erythrotherium parringtoni Crompton, 1964 [139]	[81]
	Haldanodon exspectatus Kühne & Krusat, 1972 [140]	[101]
	Megazostrodon rudnerae Crompton & Jenkins, 1968 [141]	[81]
	Morganucodon watsoni Kühne, 1949 [142]	[143]
	Morganucodontidae indet. PIN 4774/1	[143]

Results

Systematic palaeontology

SYNAPSIDA Osborn, 1903 [144]

THERAPSIDA Broom, 1905 [145]

CYNODONTIA Owen, 1861 [146]

?TRITYLODONTIDAE Cope, 1884 [147]

Saurodesmus robertsoni Seeley, 1891 [1]

Holotype: NHMUK PV OR 28877, almost complete right femur (Fig 2).

Fig 2. *Saurodersmus robertsoni* NHMUK PV OR 28877, right femur (A–B, D–I) and associated rock fragment with natural mould of the posterior surface of the bone (A, C) in anterior (A, C, D), posterior (B, F), lateral (E), medial (G), proximal (H), and distal (I) view.

Diagnosis: NHMUK PV OR 28877 can be distinguished from all other non-mammaliaform and early mammaliaform cynodonts by the following combination of femoral features: comparatively short diaphysis, lateromedially expanded proximal and distal regions, well-developed trochanters, proximodistally long and laterally flaring greater trochanter, lesser trochanter proximally placed and mostly medially projected, intertrochanteric and adductor fossa separated by a poorly-developed intertrochanteric crest, well-developed medial epicondyle, medial (tibial) and lateral (fibular) condyles similar sized in ventral aspect, pocket-like fossa proximal to the medial (tibial) condyle ventrally, “semicolon-shaped” distal articular surface.

Provenance and age: The holotype comes from the Linksfield locality (Elgin, UK). The bearing levels were interpreted to be of Rhaetian (Late Triassic) age [9,53–55].

Description

NHMUK PV OR 28877 (Fig 2) is interpreted here as a cynodont femur based on its general form, lateromedially expanded and dorsoventrally flattened proximal and distal ends with a semi-circular proximal fossa and well-developed distal condyles (ruling out non-stylopodial bones), and the absence of structures identifiable as ectepicondylar and/or entepicondylar foramen or groove (ruling out humeri of most synapsids and reptiles; see Discussion).

It is a stout element, with a relatively short and nearly straight diaphysis in dorsal/ventral view. The shaft shows some damage, particularly medially and dorsally, and has no distinct morphological features. The ventral and the distal half of the lateral surface of the diaphysis are flat whereas the dorsal surface is rounded. Its narrowest point (both in the dorsoventr[illegible]nd mediolateral aspects) is located around the middle of the preserved bone's length, indicating that it was slightly distal to mid-length when the proximal region was intact.

The proximal region has a dorsal curvature regarding the diaphysis as observed medially/laterally. The proximal end is triangular in the dorsoventral aspect. Its medial edge (Fig 2G, H) is straight and rounded, the lateral edge is sharper and gently convex laterally and proximoventraly (Fig 2E, H). The femoral head is broken off at the base alongside the top of the greater trochanter (Fig 2A, E, G, H). Therefore, the shape, size, and precise direction of the head, as well as the presence and morphology of the neck are unknown. Likewise, the apex of the lesser trochanter is damaged, however, it is clearly separated from the femoral head by a very small saddle-shaped notch on the edge of the intertrochanteric fossa (Fig 2A, D, F), bearing a low but sharp ridge. The ventromedially oriented lesser trochanter is crest-like with a somewhat expanded proximal tip. The tip of the lesser trochanter was distal to the proximal tip of the greater trochanter (Fig 2). Ventrally, the shallow intertrochanteric fossa proximally and the adductor fossa more distally are separated by a low

(~~medially more distinct than laterally~~), delicately bowed, lateroproximodorsally directed intertrochanteric crest (Fig 2F). The adductor fossa is rounded. The dorsal surface of the femur, distal to the missing femoral head is convex, bears several shallow, longitudinal vascular grooves. The proximoventrally facing surface distal to the adductor fossa is nearly flat. There is no third trochanter.

The distal end is nearly as lateromedially wide as the proximal end and ~~flares~~ similarly flared (Fig 2). Its lateral face is flat dorsoventrally and gently concave proximolaterally proximodistally. Medially, the distal region of the bone is narrower than laterally, with a rough proximal portion. A triangular medial epicondyle with sharp edges points ventrally. The dorsal surface of the distal end of the bone projects three rounded eminences separated by two shallow, wide concavities (the lateral one being the laterally shifted patellar groove). The lateral eminence is associated with the lateral (fibular) condyle; the middle one, located at the level of the long axis of the bone, is associated with the lateral limit of the medial (tibial) condyle; and the medial one (less profound) associated with the medial epicondyle. The surfaces of the lateral (fibular) and medial (tibial) condyles and of the medial epicondyle are damaged (Fig 2A, D, I), but the shape of the distal end of the bone is not substantially altered. The distal articular surface of the bone can be roughly characterized as “semicolon-shaped”: the lateral (fibular) condyle is large and oval; the intercondylar fossa is distinct but not very deep; and the medial (tibial) condyle is elongated-ovoid, rounded dorsolaterally and pinched ventromedially, projecting further posteriorly than the lateral condyle (Fig 2I). The distal reach of both condyles is roughly the same (Fig 2A, B, D, F). Ventrally, the proximal extent of the articular surfaces of both condyles is comparable and relatively small, lower than they are wide (Fig 2B, F). The lateromedial extension of the medial (tibial) condyle is smaller than that of the lateral (fibular) one, and both reach the respective edges of the epiphysis (Fig 2F); the medial (tibial) condyle extends medially slightly past the epicondyle. The proximal edges of the condyles are well marked and associated with two small but distinct fossae (Fig 2B, F). Particularly the medial fossa is clearly delineated distally and medially by a fold-like ridge, which continues towards the tibial epicondyle. The popliteal fossa is very shallow and indistinct (Fig 2~~B~~, F).

Overall, the current state of the specimen is virtually the same as in the 1800s, except of a small crack (not shown in any historical figure) lateral to the hole in the middle of the shaft and an additional transverse break through the shaft (separate from the two old glue-filled transverse cracks shown in previous figures) which was not indicated by Stiven (Fig 1) and Duff [6] but was shown by Seeley [1], and thus possibly occurred during the process or after the bone was separated from the matrix in 1889 [1]. At least during Stiven's time (Fig 1), the hole in the middle of the shaft was not yet filled with yellow glue; Duff's [6] and Seeley's [1] figures are black and white and somewhat idealized, so provide no information in that regard.

Interestingly, Stiven's illustration (Fig 1) shows the specimen upside-down, suggesting that at least initially the notch between the femoral head and the lesser trochanter could have been interpreted as an ectepicondylar foramen, and the worn surface of the distal end as a poorly preserved humeral head and the greater and lesser trochanters. Admittedly, until the bone was fully removed from the matrix to reveal its ventral structures, its morphology could have been even more confusing.

Discussion

Although the taxonomic validity of *Saurodesmus robertsoni* was never questioned (despite R. Lydekker in his comment to Seeley's [1] paper , considering it "not worthy of being made a type of a genus"), its systematic placement was always troublesome. Since its discovery, NHMUK PV OR 28877 was referred or compared to representatives of a wide spectrum of amniote groups (turtles, crocodiles, generalized crurotarsans, procolophonians, mammals, dinocephalians, and cynodonts), but these attributions rarely stood the test of time and changed repeatedly. Despite these numerous attempts at identification, most recently, the taxon was deemed an indeterminate reptile [44,45]. This uncertainty resulted not only from the incompleteness and odd morphology of the specimen, but also immense developments in vertebrate palaeontology which took place since the early 1840s, when the specimen was first studied, most notably in the state of knowledge concerning the temporal distribution, relationships, and morphological evolution of various amniote lineages. For example, at

the time it was still a relatively common practice to assign Mesozoic or even Palaeozoic vertebrate finds to extant genera [5,148–151], which signifies how different the perception was of possible taxon duration and inclusiveness. Adding the much smaller array of comparative material available back then, many of the interpretations were based on studies of much younger material, frequently not representative of Triassic taxonomic and anatomical diversity, and thus at least partly speculative [1,152,153]. For that reason, historical arguments concerning the identity of *Saurodesmus robertsoni* need to be re-evaluated.

Pantestudinata

As correctly noted by Seeley [1], the similarity of NHMUK PV OR 28877 to turtle limb bones are mostly restricted to the presence of a U-shaped fossa ventrally on the proximal end and a vague resemblance of the overall form. However, upon closer examination, these features strike as only superficial, and more differences become apparent. Seeley [1] listed a number of characters distinguishing NHMUK PV OR 28877 from turtle stylopodial bones (given the nomenclature used by him, mostly humeri, but most characters are also applicable to femora): (1) presence of a medullary cavity (contested by Lydekker in his comment published alongside Seeley's [1] paper); (2) straight rather than dorsoventrally (anteroposteriorly, in cynodont terms) sinuous profile; (3) supposed lateral and medial processes ("radial and ulnar crests", i.e., greater and lesser trochanters, according to our interpretation) separated by a wide fossa; (4) distinctiveness and small size of the supposed lateral process; (5) unusual development, convexity, and roughness of the supposed medial process; (6) larger thickness of the shaft at the contact with the head; (7) form of transverse curved ridges crossing the supposed intertubercular fossa; (8) incongruent morphology of the distal end: unusual broadness, flattening and concavity of the lateral ("ulnar") edge, compression of the medial ("radial") side into a convex edge, dissimilar distal articular surface, and absence of ectepicondylar groove or foramen. Nonetheless, the interpretation of NHMUK PV OR 28877 as a turtle femur gained much traction throughout the specimen's history [5,6,37–40,7,9,11,19,30,32–34], although it was almost never

actually supported by morphological arguments. Nopcsa [30] argued for its identity as a pleurodiran femur, noting the structural resemblance of its proximal end to both the turtle humeri and femora and the lack of the ectepicondylar foramen (refuting identification as a humerus). He interpreted the more elaborate development of its distal articular condyles as an ancestral trait due to its Triassic age and the secondary simplification of the knee morphology in pleurodires related to their aquatic ecology. It must be stressed that Seeley's [1] assessment was based only on extant turtles (limb material of Triassic pantestudinates being completely unknown at the time), and Nopcsa [30] had at his disposal only information on the proximal end of the femur of *Proganochelys quenstedti* (considered a cryptodire at the time) published by Jaekel [154], therefore they had no possibility of comparisons with actual Triassic turtles.

Quite surprisingly, Nopcsa [32], the English editors of the (posthumously published [155]) second English edition of Zittel's "Grundzüge der Paläontologie" [33], Romer [34], Bergounioux [37], and Huene [38] interpreted NHMUK PV OR 28877 as a possible proterochersid femur. Curiously, this assignment (or the specimen itself) is not present in the original German version of "Grundzüge der Paläontologie" ([156] and earlier editions) and likely was an initiative of A. S. Woodward, responsible for revisions and additions to the second English edition, and/or R. Broom, F. Nopcsa, or F. von Huene, who were acknowledged "for help with the fossil reptiles" [33]. In a sense, this view was first presented by Lydekker [14], who rather haphazardly suggested an affinity of NHMUK PV OR 28877 with the German Norian turtle '*Chelytherium obscurum*', which later turned out to be a synonym of *Proterochersis robusta* [17,18]; however, at the time neither the name *Proterochersis* (introduced by Fraas [16]) nor the family Proterochersidae (named by [30]) existed. Lyddeker [14] was explicitly aware of *Proganochelys quenstedti* (named by Baur [68]) and convinced of its taxonomic distinctiveness from '*Chelytherium obscurum*', therefore the reasoning behind this referral is unclear. Curiously, the assignment of NHMUK PV OR 28877 to the Proterochersidae was never justified in any way; in fact, realistically it never could be, due to the complete lack of limb material attributable to the Proterochersidae at the time, which did not stop Nopcsa [32] from listing "femur with wings on proximal end" as one of the diagnostic characters of the Proterochersidae. The authors might have

already been familiar with the general morphology of Triassic turtle humeri ([157]: probably *Proganochelys quenstedtii*) but their insight into femoral anatomy most likely was still limited, both taxonomically and when it comes to specimen number and quality. In addition to Jaekel's [154] partial femur of *Proganochelys quenstedti* housed in the Berlin Museum, in 1956 the Staatliches Museum für Naturkunde Stuttgart already had in its collection limb bones of the same species (a badly crushed distal end of femur SMNS 15759 and much better preserved and more complete bones of SMNS 16980, SMSN 17203, and SMNS 17204; [158–161]) but their first detailed description and documentation was published decades later [162] and the first stylopodial skeleton of proterochersids was discovered only very recently [67,163,164]. Its recovery enables testing of the hypothesis about the proterochersid identity of *Saurodesmus robertsoni*. Moreover, recent discoveries of non-testudinate pantestudinates [165–167] and new Triassic turtle species [66,67,168] allow a better insight into the variability and evolution of limb bones at the brink of turtle lineage and re-evaluation of the arguments for and against the turtle relationships of *Saurodersmus robertsoni*.

As correctly noted by Seeley [1] and Nopcsa [30], the absence of the ectepicondylar foramen or groove generally discredits NHMUK PV OR 28877 as a turtle humerus, since the groove or foramen is present in all known representatives of the Pantestudinata [157,162,165,166,169–171] (Fig 3) except *Pappochelys rosinae* Schoch & Sues, 2015 [167,172]; even in small humeri of supposedly juvenile individuals of Triassic turtles (*Proganochelys quenstedtii* SMNS 17203: Fig 3S–X [162]; *Proterochersis porebensis* ZPAL V. 39/442, ZPAL V. 39/444, ZPAL V. 39/446: [164]), a sharply defined, deep ectepicondylar groove spans along most of the dorsal surface of the distal humeral expansion, therefore its absence in NHMUK PV OR 28877 cannot be explained by ontogeny or surface damage. *Eunotosaurus africanus* Seeley, 1892 [173] has both the ectepicondylar foramen and the entepicondylar foramen [171], the latter of which is absent in Triassic Pantestudinata. The absence of foramina or grooves, however, is not the only difference between NHMUK PV OR 28877 and pantestudinate humeri. As noted by Seeley [1], the intertubercular fossa in pantestudinate humeri lacks transverse ridges [157,162–164,169–172,174,175], NHMUK PV OR 28877 is straighter than Triassic pantestudinate humeri, and its distal end lacks the characteristic deflection opposite to the proximal

end, which is observed in all Triassic and later pantestudinates [157,162,164–166,169,172]. Although the exact angle at which the proximal head was set in NHMUK PV OR 28877 relative to the shaft is uncertain [1], the whole proximal expansion is in fact more parallel to the distal expansion than in any pantestudinate with known humeri [162,164–166,169–172,174]. Also the asymmetry of the distal end, with one edge flattened and concave proximodistally and the other convex and sharpened, and wide (spanning the whole width of the bone) but restricted proximally articular surfaces of the distal condyles were correctly indicated by Seeley [1] as incongruent with pantestudinate humeri. All Triassic pantestudinates have their distal humeral expansions relatively symmetric with two roughly symmetrically developed epicondyles and straight or only gently curved and mostly rounded edges [157,162,164–166,169,170,172]. In *Eunotosaurus africanus* and all Triassic Pantestudinata in which the ventral surface of the humeri is exposed and well ossified articular surfaces, the trochlea and capitellum are restricted in their mediolateral extent, clearly separated from the maximum mediolateral extrema of the epicondyles, and never as wide relative to their proximal extent as the articular surfaces in NHMUK PV OR 28877 [157,162,164,165,169–172]. Finally, in the humeri of Triassic Pantestudinata the medial condyle is never as pinched out as the medial condyle in NHMUK PV OR 28877 [162,164,169] and the epicondyles lack separate dorsal eminences, so the dorsal surface of the bears two rather than three eminences separated by a single rather than two fossae [162,164,166,169,170,172]. Seeley [1] also formulated several arguments regarding the shape and size of the proximal processes and the supposed intertubercular fossa, as well as the thickness of the shaft. These characters are variable in Pantestudinata, including Triassic taxa, although the proximal processes are indeed poorly developed (small, thin, and weakly protruding) compared to the lateral and medial humeral processes of Triassic turtles and whereas the fossa is not particularly wide relative to the Triassic pantestudinate intertubercular fossae, it is comparatively very shallow [157,162–165,169,170,172,175].

Fig 3. Comparison of *Saurodersmus robertsoni* NHMUK PV OR 28877 (A–F) with humeri of turtles. G–L, *Proterochersis porebensis* ZPAL V. 39/156 (Proterochersidae, right, juvenile); M–R,

Proterochersis porebensis ZPAL V. 39/50 (Proterochersidae, right, adult); S–X, *Proganochelys quenstedti* SMNS 17203 (Proganochelyidae, left mirrored to right, juvenile); Y–D′, *Proganochelys quenstedti* SMNS 16980 (Proganochelyidae, right, subadult); E′–J′, *Palaeochersis talampayensis* PULR 068 (Australochelyidae, right); K′–P′, *Trionyx triunguis* UF H65522 (Cryptodira: Trionychidae, right). A, G, M, S, Y, E′, K′, dorsal view; B, H, N, T, Z, F′, L′, medial (posterior) view; C, I, O, U, A′, G′, M′, ventral view; D, J, P, V, B′, H′, N′, lateral (anterior) view; E, K, Q, W, C′, I′, O′, proximal view; F, L, R, X, D′, J′, P′, distal view. Ectepicondylar grooves/foramina indicated by red arrowheads. Not to scale.

Interpretation of NHMUK PV OR 28877 as a pantestudinate femur is seemingly more appealing due to the lack of foramina or grooves in that bone (Fig 4), but most of the other features listed above also preclude such an identification, even if they are less clearly expressed in pantestudinate femora than in humeri. The femur of *Eunotosaurus africanus* exhibits a completely different form – it is straight, more rod-like, with weaker flattening of the proximal and distal end and much less distinct morphological features [171]. In *Pappochelys rosinae*, the femur is relatively straight but slenderer and less expanded at the ends than NHMUK PV OR 28877, with more elongate, very narrow, and deeper intertrochanteric fossa [167,172]. In *Eorhynchochelys sinensis* Li *et al.*, 2018 [166] and *Odontochelys semitestacea* Li *et al.*, 2008 [165], the curvature along the shaft is comparable as in NHMUK PV OR 28877 [165,166], but in Testudinata it is stronger, which is particularly visible in more pronounced deflection of the distal end relative to the femoral head [67,162,169,175]. Femoral torsion is less severe than humeral torsion but still higher in all Triassic pantestudinates than in NHMUK PV OR 28877 [162,164–166,169,172] and most pronounced particularly in Triassic non-testudinate pantestudinates [165,166,172]. The morphology of trochanters and intertrochanteric fossa is varied in Pantestudinata but generally in Triassic species the fossa is narrower, deeper, and devoid of transverse ridges, the trochanters in proximal view form a distinct angle of 90° or less, and particularly the greater trochanter is more upright and thicker than in NHMUK PV OR 28877 [67,154,162,166,169,172,175,176]. The proximal end of the Triassic pantestudinate femora is also

bulkier than in NHMUK PV OR 28877 [67,154,162,165,169,172,175,176]. The posterior flattening of the shaft resembles the condition seen in Triassic pantestudinate femora [67,162,169,172,175,176] but the structure of the distal end is mediolaterally reversed relative to NHMUK PV OR 28877: in pantestudinates, the anterior (lateral) face of the distal expansion is typically flattened and may be concave whereas the posterior (medial) edge is sharpened [67,162,166,169]. Furthermore, in Triassic pantestudinates the distal femoral condyles are not aligned mediolaterally with the edges of the distal expansion as in NHMUK PV OR 28877 but the lateral (fibular) condyle is located closer to the midwidth of the distal expansion and further distally than the medial (tibial) condyle [67,162,163,166,169,172]. Unlike in NHMUK PV OR 28877, both femoral condyles in pantestudinates project medially distinct ridges along the ventral surface of the distal expansion, thus the popliteal fossa is well delineated and medially (posteriorly) a second triangular fossa is present more posteriorly, between the ridge of the lateral (fibular) condyle and the fibular epicondyle [67,162,163,166,169,172]. Finally, there is no epicondylar eminence dorsally [67,162,165,166,169,172]. Thus, the distal articular morphology in Triassic Pantestudinata is indeed more elaborate than in many extant taxa, as speculated by Nopcsa [30], but its configuration is different than in *Saurodesmus robertsoni*.

Fig 4. Comparison of *Saurodersmus robertsoni* NHMUK PV OR 28877 (A–F) with femora of turtles. G–L, *Proterochersis porebensis* ZPAL V. 39/432 (Proterochersidae, right, Late Triassic); M–R, *Proganochelys quenstedti* SMNS 17203 (Proganochelyidae, left mirrored to right, juvenile, Late Triassic); S–X, *Proganochelys quenstedti* SMNS 16980 (Proganochelyidae, left mirrored to right, subadult, Late Triassic); Y–D′, *Palaeochersis talampayensis* PULR 068 (Australochelyidae, left mirrored to right, Late Triassic); E′–J′, *Trionyx triunguis* UF H65522 (Cryptodira: Trionychidae, right, extant); K′–P′, *Chelus fimbriatus* UF H85199 (Pleurodira: Chelidae, right, extant). A, G, M, S, Y, E′, K′, dorsal view; B, H, N, T, Z, F′, L′, medial (posterior) view; C, I, O, U, A′, G′, M′, ventral view; D, J, P, V, B′, H′, N′, lateral (anterior) view; E, K, Q, W, C′, I′, O′, proximal view; F, L, R, X, D′, J′, P′, distal view. Not to scale.

We were not able to unambiguously confirm the presence of a distinct medullary cavity in NHMUK PV OR 28877 – at the moment of our study the bone was separated into two parts, but the surface of the break was obscured by glue remnants and sediment particles, and a region filled with thin trabeculae (if not completely empty). Regardless, the differences in morphology extending beyond the variability observed within the Pantestudinata and not consistent with observed evolutionary trajectories between the earliest known pantestudinates (*Eunotosaurus africanus*) and Triassic Testudinata allow to exclude the possibility that *Saurodesmus robertsoni* is a representative of that lineage.

Parareptilia

Parareptilian resemblance of *Saurodesmus robertsoni* have been indicated twice in the literature. Seeley [21] perceived NHMUK PV OR 28877 to be similar to the femur of *Bradysaurus* (‘*Pareiasaurus*’) *baini* (Fig 5G–L) based on the shape of the trochanters and the presence of a rugosity on the laterodorsal surface, which led him to the idea that NHMUK PV OR 28877 may represent an anomodont femur (note that he considered the Anomodontia to include at least the theriodonts, dicynodonts, placodonts, pareiasaurs, procolophonids, and mesosaurs). Seeley [177] also noted some resemblance of the intertrochanteric fossa on the proximal end of NHMUK PV OR 28877 with that on the femur of *Procolophon* sp. (also comparing it to the homologous structure of birds, mammals, and turtles), although he earlier considered procolophonid humeri as anatomically different [1] from NHMUK PV OR 28877.

Fig 5. Comparison of *Saurodersmus robertsoni* NHMUK PV OR 28877 (A–F) with femora of non-diapsid reptiles. G–L, *Bradysaurus baini* NHMUK PV R 4066 (Pareiasauria, right, middle Permian); M–R, *Labidosaurus hamatus* MB.R.4433 (Captorhinidae, right, early Permian, cast); A, G,

M, anterior view; B, H, N, lateral view; C, I, O, posterior view; D, J, P, medial view; E, K, Q, proximal view; F, L, R, distal view. Not to scale.

Incidentally, the characters pointed out by Seeley as common between the procolophonids and pareiasaurs and *Saurodesmus robertsoni* are among the characters historically proposed as suggesting phylogenetic relationships between all of those taxa and turtles [178–181]. They seem to simply reflect a shared similarity resulting from various degrees of modification of an ancestral amniote morphology and perhaps some biomechanical locomotor parallelisms rather than strongly indicating a shared ancestry [182,183].

Despite having those basic anatomical components and their significant dorsoventral compression, pareiasaur femora differ from NHMUK PV OR 28877 in a number of ways: they are much broader, the result of a significantly broader or wider shaft, which is at least 50% as wide as the proximal and distal expansions; the direction of the femoral head is subparallel to the plane of the distal expansion; the internal trochanter is located more distally and is subperpendicular to the proximal expansion; the greater (major) trochanter typically extends distally along most of the shaft's length a flattened postaxial flange (usually resulting in the posterior edge being straighter than the anterior edge); the internal (lesser) trochanter and adductor ridge is usually well developed, elongated, often sigmoid-shaped and somewhat medially positioned, away from the anterior (preaxial) edge of the femur; the dorsal surface of the distal expansion is typically pierced by a large and deep nutrient foramen and groove, elongated along the direction of the shaft; the distal end is not significantly flattened; the distal lateral condyle often extends significantly beyond the distal medial condyle (related to a relatively sprawling or oblique hind-limb posture); the articular surfaces of the condyles are longer than wide, oval in outline, with sharply defined, flattened, and substantial ventral exposure, and are separated by a deep intercondylar groove (sulcus) [178,179,191,181,184–190] (Fig 5G–L). There is no Triassic record of pareiasaurs, since the clade perished at the Permo-Triassic boundary [184,187,191].

In comparison to *Saurodesmus robertsoni*, the femur of procolophonoids is more rod-like, its proximal end is more massive and less expanded, the trochanters are more parallel to the long axis of the bone, the intertrochanteric fossa is deeper and narrower, the shaft is thicker, the popliteal and intercondylar fossae are much deeper and better defined, the distal condyles are narrower, and the edges or the bone are rounded rather than sharpened [177,192–197]. Unlike pareiasaurs, procolophonoids did persist into the Triassic [193].

We feel it is not necessary to compare NHMUK PV OR 28877 to femora of other parareptilian clades, such as the small aquatic mesosaurs, the 'nycteroleters', the bolosauridae, and the lanthanosuchoidae, since none of these clades, besides for procolophons, survived the Permo-Triassic extinction event [198], none of these clades have previously been compared to specimen NHMUK PV OR 28877 in the literature, and the specimen's morphology is very different from the femora of these clades.

Non-diapsid Eureptilia

The humeri of earliest eureptiles, such as captorhinids (Fig 5M–R) or *Protorothyris archeri* Price, 1937 [199], retain numerous plesiomorphic characters, e.g., poorly differentiated humeral head, proximal and distal expansions set at about a right angle, massively expanded, plate-like distal end, retention of the ectepicondylar foramen, and presence of a prominent, ovoid capitellum [162,200–203], and thus are dissimilar to NHMUK PV OR 28877. Conversely, the femora correspond with NHMUK PV OR 28877 in some general, plesiomorphic characters, such as the presence of a distinct intertrochanteric fossa and lesser trochanter or distinctly expanded distal end, as well as the thickness of the lateral epicondyle and sharpening of the medial epicondyle, but differ, e.g., in more ventrally directed lesser trochanter, less dorsally offset femoral head, narrower and thicker proximal end, larger robustness and stoutness, clearly bipartite rather than tripartite distal end with deeply incised patellar groove, and the distal projection of the lateral condyle beyond the medial condyle [162,200,201,203]. There are no representatives of non-diapsid eureptiles known from the Triassic [203,204].

‘Younginiformes’

In the humeri of the ‘Younginiformes’, e.g., *Youngina capensis* Broom, 1914 [205], the proximal and distal parts of the shaft are rotated at 90 degrees to each other, and have a clear entepicondylar foramen and ectepicondylar groove [206–208]. The femur seems to have a sigmoidal shape and lacks significant proximal and distal expansions [208]. Both humerus and femora are slim compared to NHMUK PV OR 28877 [207,208], therefore it seems unlikely that NHMUK PV OR 28877 might belong to this group.

Sauropterygia

In sauropterygians, humeri and femora do not form such well distinguished proximal and distal structures as those in NHMUK PV OR 28877 [209–212]. In nothosaurs, the humerus is thick and bowed, and the femur is straight with slightly expanded proximal and distal end [211]. The humeri and femora of placodonts (e.g., *Placodus gigas* Agassiz, 1833 [213]) have a similar shape in general [212,214]. In plesiosaurs, in which the limbs evolved into fins, both humeri and femora, have a similar morphology with much more expanded distal end in proportion to the proximal end [209,210].

Choristodera

In Choristodera, the proximal and distal ends of the humerus are significantly twisted in relation to each other at an angle of about 45 to 60 degrees, and are moderately expanded, compared to NHMUK PV OR 28877. They have a distinct deltopectoral crest, and at least some have the ectepicondylar groove which is lacking in NHMUK R28877 [215–219].

The femora of Choristodera have a straight shaft with a weakly defined femoral head, their distal condyles ~~and~~ are much slenderer in comparison to NHMUK PV OR 28877, and they have a distinct internal trochanter [215–219].

Although the Choristodera have a very wide temporal range extending from the Middle Jurassic to Miocene [220], it seems that the morphology of their stylopodia remains conservative. A single species, *Pachystropheus rhaeticus*, is known from the Late Triassic (Rhaetian) of southwest England [218,219,221], but its taxonomical affiliation to Choristodera was disputed, mostly due to its poor preservation [220,222]. Nevertheless, the morphology of its stylopodia is consistent with the Chorsitodera [218,219].

Panarchosauria

Basal panarchosaurs

The humeri of basal panarchosaurus, such as *Aenigmastropheus parringtoni* Ezcurra *et al.*, 2014 [223], a late Wuchiapingian stem archosauromorph, differ from NHMUK PV OR 28877 in the presence of the ectepicondylar groove, capitellum and trochlea separated from the epicondyles, and depressed surfaces of the platelike distal expansion [223]. A similar morphology is expected in other basal representatives of the Panarchosauria, refuting interpretation of NHMUK PV OR 28877 as a humerus of a representative of an early-branching steam archosaur lineage.

‘Protorosauria’

‘Protorosaurian’ humeri differ from NHMUK PV OR 28877 in weakly expanded proximal ends with a simple, proximally directed articular surface and no separate, laterally directed processes, simpler, plate-like predominantly unilaterally (medially) expanded distal ends of humeri with distinct ectepicondylar notch, as well as sinuous femora with upright trochanters and little to no proximal and

distal expansion [206,223–225]. Although mostly known from the Middle Triassic, some representatives of the group survived to the Late Triassic [226].

Allokotosauria

Humeri of Allokotosauria are distinguished from NHMUK PV OR 28877 based on their laterally expanded proximal articular surface without separation of the processes, larger torsion, presence of the ectepicondylar groove and distinct fossae on the dorsal and ventral surfaces of the distal expansion, and lack of flattening of the shaft distal the intertubercular fossa and along the entepicodylar edge of the distal end [227–230]. The shape of the distal end and angle between the proximal and distal expansions differ between allokotosaur taxa, *Trilophosaurus buettneri* Case, 1928 [231] (Trilophosauridae) and *Azendohsaurus madagaskarensis* Flynn *et al.*, 2010 [232] (Azendohsauridae) contrast with NHMUK PV OR 28877 in the presence of a prominent entepicondyle and distal articular condyles positioned closely together and separated from the edges of the distal expansion [229,230] while *Malerisarus* spp. (Azendohsauridae) exhibits a much stronger humeral torsion [227,228]. Femora of allokotosaurs resemble NHMUK PV OR 28877 in their rather simple, triangular distal ends with limited proximal reach of the articular surfaces but fifer in very weakly expanded proximal end, ventral direction of the internal trochanter and rounded edges of the distal end [227–230]. Additionally, azendohsaurids have clearly sinuous femora torsion [227,228,230] while *Trilophosaurus buettneri* differs in ventrally protruding tibial condyle and straight tibial edge of the distal end [229].

Rhynchosauria

Seeley [1] simply deemed rhynchosaurian humeri dissimilar to *Saurodesmus robertsoni* but did not elaborate. Since the Rhynchosauria is represented in the Late Triassic of the Elgin area [233–235], it deserves consideration. Humeral anatomy of rhynchosaurs shares with NHMUK PV OR 28877 the

presence of the proximal and distal expansion but differs in the presence of a wide and continuous proximal articular surface encapsulating the medial and lateral processes, proportionally larger proximal end, large torsion of about 90°, presence of the ectepicondylar foramen, symmetry of the distal expansion with distinct anterior and posterior fossae, and roundness of the distal condyles and entepi- and ectepicondylar edges [234,236,237]. In fact, NHMUK PV OR 28877 is slightly more reminiscent of rhynchosaur femora, due to their less expanded proximal end, distinct internal trochanter, lack of pronounced torsion, simpler morphology of the distal end and wider than long articular surfaces of the distal condyles with anterior condyle extending slightly more proximally than the posterior condyle; however, it is slenderer, lacks the clearly ventral direction of the internal trochanter, defined adductor crest, and triangular fossae in the middle of the ventral and dorsal surface of the distal end, and differs in the non-equidimensional distal condyles, less rectangular distal articular surface, and sharpened anterior edge of the distal end [234,236,237].

Stem Archosauria

Overall, Archosauriformes more distantly related to archosaurs have their stylopodial bones developed similarly to the more derived representatives of the group: the humeri having transverly expanded proximal and distal ends; deltopectoral crest distinctly parallel to the humeral head; thick deltopectoral crest and internal tuberosity (compared to NHMUK PV OR 28877 if interpreted as a humerus), and having both distal condyles round shaped (one is acute in NHMUK PV OR 28877) with clear entepi- or ectepicondyle foramina (absent in NHMUK PV OR 28877); the femora being distinctly sigmoidal, femoral head oriented medially, and prominent fourth trochanter or internal trochanter in the Proterosuchidae or Erythrosuchidae (all of these features are absent in NHMUK PV OR 28877). Those characteristics are fully applicable to the basal members of the Eucrocopoda (encompassing Avemetatarsalia and Pseudosuchia), including the Euparkeriidae [238–240] and Proterochampsia [241–245], which have their stylopodial bones more or less alike their more derived

cousins. Therefore, that excludes the possibility of NHMUK PV OR 28877 belonging to one of those groups.

Little is known about the limbs of the basal eucrocopodan clade, the Doswelliidae. The stylopodial bones are preserved exclusively in *Archeopelta arborensis* Desojo *et al.*, 2011 [246], *Doswellia kaltenbachi* Weems, 1980 [247] (femur), and *Rugarhynchos sixmilensis* (Heckert *et al.*, 2012) [248] (distal femur) [246–252]. However, in the latter two, the bones are too poorly preserved for adequate anatomical comparisons [248,250,252]. In the case of *Archeopelta arborensis*, only the proximal part of the humerus is preserved. Citing the description of the authors, "the humeral head of *Archeopelta* is proximomedially deflected" [246], so in the dorsoventral aspect the lateral edge of transversally expanded proximal part of the shaft is bevelled and slopes diagonally mediolaterally in reference to the straight middle portion of the shaft. This differs from other archosauriforms, in which the lateral edge of the proximal expansion is a straight continuation of the middle portion of the shaft or expands it laterally; the same is true for NHMUK PV OR 28877 (if interpreted as a humerus). The femur of *Archeopelta arborensis* has its general form similar as other archosauriforms [246]. Some analyses [206,253] also recover *Vancleavea campi* Long & Murry, 1995 [251] as a member of the Doswelliidae. However, neither of the stylopodial bones of *Vancleavea campi* reassemble NHMUK PV OR 28877 in general shape. Both the humerus and femur have less developed heads and condyles, and are more gracile in comparison to NHMUK PV OR 28877. The humerus has a well-developed deltopectoral crest and equally-sized and shaped distal condyles. The only similarity is the lack of ectepi- or entepicondylar grooves [254]. The femur of *Vancleavea campi* is sigmoidal with twisted femoral head and condyles. It does not have the fourth or internal trochanter [254], which could be considered a similarity with NHMUK PV OR 28877, if not for the entirely different shape of the shaft.

In the most basal of the Archosauriformes (Proterosuchidae and Erythrosuchidae) the humeri resemble NHMUK PV OR 28877 in their most general shape due to the significant and equal transverse expansion of the proximal and distal parts, as well as their general robust shape. However, NHMUK PV OR 28877 is more gracile and lacks other mentioned features of the humerus [206,255]. The femora of the Proterosuchidae and Erythrosuchidae also slightly differ morphologically from the

more advanced archosauriformes. However, they are also different form NHMUK PV OR 28877 in having much less significant proximal and distal expansions compared to the middle portion of the shaft and in having a well-developed internal trochanter [255].

Kuehneosauridae

In the gliding Kuehneosauridae, both the humerus and femur are slender and gracile compared to NHMUK PV OR 28877 [256–259]. All known kuehneosaurids are also considerably smaller [257,259]. In the Kuehneosauridae, both the proximal and distal ends of the humeri are expanded, but proportionally not as strongly as in NHMUK PV OR 28877, and the middle portion of the shaft in the Kuehneosauridae is proportionally much longer than in NHMUK PV OR 28877 [256–258]. They also have a distinct entepicondylar foramen which is absent in NHMUK PV OR 28877 [256,257]. The femur in the Kuehneosauridae is a long, straight bone with medially oriented femoral head, and thus not fitting NHMUK PV OR 28877 [256–258]. In addition, long bones of kuehneosaurids have a hollow shaft [259], in contrast to NHMUK PV OR 28877.

Pseudosuchia

Seeley [1] originally identified *Saurodesmus robertsoni* as a member of “a primitive crocodilian stock” representing a new suborder of Crocodylia with a mix of crocodilian and lacertilian characters. He noticed some resemblances of NHMUK PV OR 28877 with the humeri of extant gavials and crocodiles (Fig 6G–L), as well as fossil crocodiles, of which he mentioned *Crocodilus hastingiae* Owen, 1848 [260] (an Eocene crocodile from the United Kingdom, now *Diplocynodon hantoniensis* [261]). Due to differences in used terminology, his description is confusing for the modern reader, making it difficult to recognize the specific characters he had in mind. Because he treated the bone as a humerus, the structure referred by him as the radial process/crest is probably the lesser trochanter (in his understanding, the deltopectoral crest – in respect to crocodile humerus; lateral process – in respect

to the humeri of the Pantestudinata). Consequently, his ulnar border and ulnar tuberosity probably mean the posteromedial edge of the proximal part of the bone, which is identified here as the greater trochanter (in his understanding, the internal tuberosity – in respect to crocodile humerus).

Fig 6. Comparison of *Saurodersmus robertsoni* NHMUK PV OR 28877 (A–F) with humeri of archosaurs. G–L, *Crocodylus porosus* Laboratory of Stephen Wroe uncat. (Crocodylia, right, extant); M–R, *Stagonolepis olenkae* ZPAL Ab. III/1175 (Aetosauria, right, Late Triassic); S–X, *Parasuchus* sp. ZPAL Ab. III/1994 (Phytosauria, right, Late Triassic); X–C′, *Silesaurus opolensis* ZPAL Ab. III/452 (Dinosauromorpha, left mirrored to right, Late Triassic). A, G, M, S, X, dorsal view; B, H, N, T, Y, medial view; C, I, O, U, Z, ventral view; D, J, P, V, A′, lateral view; E, K, Q, W, B′, proximal view; F, L, R, X, C′, distal view. Ectepicondylar groove indicated by red arrowheads. Not to scale.

Seeley [1] noted three similarities between NHMUK PV OR 28877 and crocodilian humeri, namely (1) the sharp inner prolongation of his radial process, which probably translates to the sharp proximal edge of the intertrochanteric fossa, as identified here (in his understanding, proximal surface of the deltopectoral crest in the crurotarsan humerus); (2) prolongation of the proximal articular surface beyond his radial process (i.e., the articular surface of the head located proximally to the lesser trochanter, as identified here); (3) hollow limb bones (by which he probably mean a well-developed medullary cavity, in contrast to turtles). However, he also noted more differences, namely that: (1) his radial crest is flexed too much dorsally – the deltopectoral crest points ventrally in crocodiles and is perpendicular to the humeral head (e.g., *Diplocynodon hantoniensis* humerus NHMUK OR 30206; [262]); (2) his ulnar (i.e., lateral, according to our interpretation) border is compressed to a sharp muscular edge (internal tuberosity and proximal posteromedial border is robust and has a smooth edge in crocodiles); (3) the proximal and distal ends are more expanded than in crocodiles; (4) the bone is straight (lack of bending of the shaft – in *Diplocynodon hantoniensis* NHMUK OR 30206 the shaft of the humerus forms a delicate arch; [262]); and (5) the condyle on his radial (i.e., medial, according to

our interpretation) side is compressed in comparison to crocodiles (which likely refers to the ventrally concave area and the sharpened edge of the tibial epicondyle; lateral condyle in respect to the crurotarsan humerus). Nevertheless, beside the compression of his ulnar (i.e., lateral) margin of the proximal end (considered by him an apomorphy relative to all other reptiles), he claimed the overall morphology homologous, and therefore settled for identification of *Saurodesmus robertsoni* as a member of the Crocodylia representing a new suborder with a mix of crocodilian and lacertilian characters.

There is a vast difference in morphology between the humerus and femur in crocodiles and their kin. The most significant is that the femora have a distinctly sigmoidal shaft, the femoral head is directed medially and substantially twisted in respect to the condyles, and there is the fourth trochanter (a knob-like feature, attachment area for the muscles retracting hind limbs) on the posterior surface of the shaft [262–266]. Therefore, the interpretation of NHMUK PV OR 28877 as a crocodilian femur can be rejected straight away and it was never discussed in the literature. The resemblance of NHMUK PV OR 28877 to the crocodilian humerus is also only superficial and restricted to the general form of the bone with its expanded proximal and distal ends, as well as its relatively straight shaft. However, in crocodiles and their relatives, the expansion of the ends is not as symmetrical as in NHMUK PV OR 28877, but rather more pronounced medially than laterally (Fig 6G–L) [262–268]. Beside the perpendicular orientation of the deltopectoral crest noted as a difference form the crocodiles by Seeley [1], both the deltopectoral crest and the internal tuberosity are large structures in the latter, being areas of attachments for the muscles responsible for major movements of the forelimbs [263–267,269,270]. Finally, there are no structures that can be referred to the ecto- and entepicondylar grooves or foramina, however, in crocodiles these might be poorly developed [262].

Interestingly, Seeley [1] mentioned that neither of the other reptile species from the Elgin Sandstone has the same resemblance to crocodiles as NHMUK PV OR 28877, which is puzzling in the context of the aetosaur *Stagonolepis robertsoni* Agassiz, 1844 [57], an armoured member of the Pseudosuchia, the clade that includes ancestors of modern crocodiles [206,271]. At the moment of Seeley's publication, the resemblance of *Stagonolepis robertsoni*

to crocodiles has been already noted by Huxley [272–274]. However, the proximal end of the humerus in aetosaurs is much more expanded transversely compared to other pseudosuchians (Fig 6M–X) [266,267], which was also noted by Seeley [1], and the exact relations between various groups now included within the Pseudosuchia were not sorted at the time [206,271], which may partly explain Seeley's [1] opinion.

It seems that Seeley's [1] identification of NHMUK PV OR 28877 as a crocodilian humerus was the only one that was supported by some discussion, and other mentions of the specimen as a crocodilian are only repeating his results [28,29,42,43]. Huene [28] arbitrarily assigned *Saurodesmus robertsoni* to his "Crurtoarsi", probably following the idea that if it is a crocodilian, it should be related to other crocodile-like reptiles. At that moment it was also considered ~~clear~~ that *Stagonolepis robertsoni* was related with other crocodile-like animals from the Germanic Basin, especially the genus *Belodon* [272–274]. However, this conclusion was mostly based on the morphology of ostederms, and at the dermal armour asigned at that time to that later genus was later proven to be the dermal armour of an aetosaur [251,275–277].

Ornithodira

According to Seeley [1], NHMUK PV OR 28877 exhibits some resemblance to pterosaurs in characters in which it is divergent from the crocodilians. This vague statement is difficult to address, but aside of the strongly mediolaterally expanded proximal end and relative gracility, there is little meaningful similarity – NHMUK PV OR 28877 lacks, ~~e.g.,~~ the anteroposterior expansion of the proximal end, subrectangular shape of the distal articulation surface, and the trochlear form of the distal articulations which were present in early Mesozoic pterosaurs [278–282].

Lagerpetids lack the ectepicondylar foramen or groove and have much simpler humeral morphology than pterosaurs, but differ from NHMUK PV OR 28877 in only very slightly deflected proximal articular surface, medial deflection of the whole proximal end, barely expanded, more

rectangular in articular view distal end, distal condyles separated by a pronounced groove, distally protruding medial condyle, greater torsion, and overall much more gracile and slender form [283–285]. Likewise, dinosauromorph humeri can be distinguished from the holotype of *Saurodesmus robertsioni* based on their overall slenderness, less deflected, predominantly proximally directed humeral head, slight medial deflection of the proximal end, lack of a separate lateral process, and barely expanded distal end with two rounded, similarly shaped and sized distal condyles (Fig 6X–C′) [71,269,285–288].

Lepidosauromorpha

A lacertilian identity of NHMUK PV OR 28877 was refuted by Seeley [1] based on its lack of twisting, smaller articular condyles, and the lack of a ridge connecting ventrally the supposed lateral crest with the supposed humeral head, although he noted that the compression along the anterior margin is similar as in lizards. Despite that, Lydekker (comment to Seeley [1]) suggested a rhynchocephalian relationship of *Saurodesmus robertsoni*. Even though fossils of true Triassic squamates were discovered only relatively recently and remain rare [289–292], Seeley's [1] arguments for rejection of such a relationship remain valid. Triassic lepidosauromorphs still had both the entepicondylar and ectepicondylar foramen, the loss of which is a derived character [290–293].

Synapsida

Possible synapsid affinities of NHMUK PV OR 28877 were brought up several times in the 19th century, but only in passing and their significance is in most cases ~~moot,~~ given, e.g., the convoluted history and unestablished contents of the group at the time, and problems concerning distinction between the synapsids, parareptiles, and placodonts ~~which were not yet solved:~~ Seeley [1] indicated some similarity of the proximal end to the Jurassic mammalian femur described by him earlier [294] but noted dissimilarity of the distal ends; later, he highlighted a similarity of either the distal end [22–

24] or the whole bone [26] to dinocephalian femora but also remarked that in *Saurodesmus robertsoni* the proximal and distal ends are more expanded, the anterior edge of the distal end is more compressed, and the trochanter minor is directed more medially and larger [26]; even later [27], he stated that NHMUK PV OR 28877 as a whole can be compared to the proximal part (the only known at the time) of the femur of his newly described cynodont, *Cynognathus crateronotus*, but he also indicated the ventral rather than anterior inclination of the trochanter minor in the latter and did not declare whether he considered the two taxa to be closely related. Huene [28] suggested, ~~as an alternative for the hypothesis of crurotarsan relationships of *Saurodesmus robertsoni* but~~ without providing any morphological evidence, its inclusion into his Lycosuchia, containing non-cynodont synapsids but also some problematic taxa, such as *Actiosaurus gaudryi* Sauvage, 1883 [295] and *Crurosaurus problematicus* Huene, 1902 [28]. Both of the latter turned out later not to be synapsids, the former being eventually reinterpreted as a choristodere (see above), and the latter remaining problematic [296,297].

Most historically cited resemblances of NHMUK PV OR 28877 with synapsids outside of the cynodont grade seem to relate to plesiomorphic characters, reflecting a generalized synapsid condition. This is certainly true for dinocephalian femora, which share with *Saurodesmus robertsoni* a shallow intertrochanteric fossa and anteroposterior flattening, but otherwise the similarity is rather faint – ~~they~~ are much stockier, lack the anterior deflection of the proximal end, the proximal and distal ends are less flared, the internal trochanter is buttonlike or ridgelike and directed posteriorly, and the anterior and posterior surfaces of the shaft bear a sublongitudinal concavity and may be crossed by a wide ridge connecting one of the condyles with the proximal end (Fig 7G–L) [26,298–300]. Although the femora of therocephalian synapsids are slenderer than those of dinocephalians and they may exhibit a similar form factor of the distal articular surface to that of NHMUK PV OR 28877 (Fig 7R), they also are characterized by less flared proximal and distal ends, shallower intertrochanteric fossa, less prominent trochanters, and straighter and ridged shafts (Fig 7M–R) [301].

Fig 7. Comparison of *Saurodersmus robertsoni* NHMUK PV OR 28877 (A–F) with femora of

non-cynodontian synapsids. G–L, *Tapinocaninus pamelae* NMQR 2987 (Dinocephalia, left mirrored to right, late Permian); M–R, *Moschorhinus kitchingi* NMQR 3939 (Therocephalia, left mirrored to right, late Permian to Early Triassic). A, G, M, anterior view; B, H, N, lateral view; C, I, O, posterior view; D, J, P, medial view; E, K, Q, proximal view; F, L, R, distal view. Not to scale.

NHMUK PV OR 28877 differs from cynodont humeri in its general form and observable structures, most notably it lacks the rotation between the proximal and distal regions, the deltopectoral crest, the entepicondylar foramen, proportionally mediolaterally expanded distal region, and close alignment of rounded distal condyles clearly separated from the epicondyles, but fits the morphology of cynodont femora (Figs 8, 9). While proximally the general outline of the bone and the ventral fossa are indeed similar to Seeley's [294] femur of a Jurassic mammal from the Stonesfield Slate, we agree with his [1] notion that the more symmetric and less mediolaterally expanded distal end with a more pronounced popliteal fossa, as well as the grooved posterior surface of the shaft are different. A much better comparison can be found with non-mammalian representatives of the Cynodontia.

Fig 8. Comparison of *Saurodersmus robertsoni* NHMUK PV OR 28877 (A–F) with femora of non-mammaliamorph cynodonts. G–L, *Thrinaxodon liorhinus* BP/1/7199 (non-eucynodont Epicynodontia, right, Early Triassic); M–R, *Cynognathus crateronotus* NMQR 1206 (Eucynodontia: stem Cynognathia, right, Middle to Late Triassic); S–X, Cynognathia indet. (*Diademodon* sp. or *Cynognathus* sp.) BP/1/1675 (Eucynodontia: stem Cynognathia, right, Middle to Late Triassic); Y-D′, *Langbergia modisei* NMQR 3255 (Cynognathia: Trirachodontidae). A, G, M, S, Y, anterior view; B, H, N, T, Z, lateral view; C, I, O, U, A′, posterior view; D, J, P, V, B′, medial view; E, K, Q, W, C′, proximal view; F, L, R, X, D′, distal view. Not to scale. Note the variable development of the hooked greater trochanter in cynognathians.

Fig 9. Comparison of *Saurodersmus robertsoni* NHMUK PV OR 28877 (A–F) with femora of

mammaliamorph cynodonts. G–P, *Tritylodon longaevus* BP/1/5089 (G–K, proximal end) and BP/1/4783 (L–P, distal end) (Tritylodontidae, left mirrored to right, Early Jurassic, casts); Q–V, *Bienotherium yunnanense* FMNH CUP 2285 (Tritylodontidae, left mirrored to right, Early Jurassic); W–B′ *Brasilodon quadrangularis* UFRGS-PV-1043-T (Brasilodontidae, right, Late Triassic; modified from Guignard *et al.* [79]); C′–H′, *Ornithorhynchus anatinus* ZPAL z.p. M. 12/13 (Mammalia: Monotremata, left mirrored to right, extant). A, G, L, Q, W, C′, anterior view; B, H, M, R, X, D′, lateral view; C, I, N, S, Y, E′, posterior view; D, J, O, T, Z, F′, medial view; E, K, U, A′, G′, proximal view; F, P, V, B′, H′, distal view. Not to scale.

The anatomy of the femur of *Saurodesmus robertsoni* is comparable to that of many non-mammaliaform cynodonts and early mammaliaforms (i.e., morganucodontans). As stated by Guignard *et al.* [79], some of the variability highlighted below may result from different ontogenetic stages, and the perceived size, shape, and extent of the different structures may be impacted by preservation. Nonetheless, *Saurodesmus robertsoni* displays a fundamentally cynodontian femoral structure.

As observed in dorsal/ventral view, the relative mediolateral expansion of the proximal and distal regions of *Saurodesmus robertsoni* femur are similar to *Chiniquodon theotonicus* Huene, 1936 [100], *Prozostrodon brasiliensis* (Barbarena, Bonaparte, & Teixeira, 1987) [129], the indeterminate tritylodontid MCZ 8838, *Langbergia modisei* (NMQR 3255), and *Traversodon stahleckeri* Huene, 1936 [100], differing from other cynodonts. Among these, the femoral proportions of *Saurodesmus robertsoni* are most similar to those of *Prozostrodon brasiliensis*.

The femoral head in NHMUK PV OR 28877 is interpreted to have been offset from the long axis of the shaft, in a degree comparable to that observed in some traversodontids (i.e., *Boreogomphodon jeffersoni* Sues & Olsen, 1990 [112], *Massetognathus ochagaviae* Barberena, 1981 [119], *Luangwa drysdalli* Brink, 1963 [118], *Traversodon stahlecker*[illegible]), probainognathians (i.e., *Probainognathus jenseni* Romer, 1970 [128], *Brasilodon quadrangularis*, *Bienotherium yunnanense*, tritylodontid indet. CXPM C2019 2A235), and morganucodontans (Morganucodontidae indet. PIN

4774/1, *Eozostrodon parvus* Parrington, 1941 [138]). On the other hand, most non-mammalian cynodonts show a less dorsally projected femoral head, including early and derived forms.

The lesser trochanter is medially and slightly ventrally projected, and dorsally exposed, separated from the femoral head by a well-defined notch as in the traversodontid *Andescynodon mendozensis* Bonaparte, 1969 [111], the early probainognathian *Trucidocynodon riograndensis* ~~De~~ Oliveira, Soares, & Schultz, 2010 [105], tritylodontids (i.e., *Tritylodon longaevus*, the unnamed tritylodontid CXPM C2019 2A235 from Lufeng, *Bienotherium yunnanense* (FMNH CUP 2285, FMNH CUP 2295), *Bienotheroides wansienensis* Young, 1982 [135], *Oligokyphus major* Kühne, 1956 [90], the indeterminate tritylodontid MCZ 8838), *Eozostrodon parvus*, *Morganucodon watsoni* Kühne, 1949 [142], the indeterminate morganucodontid PIN 4774/1 from Peski, and *Haldanodon exspectatus* Kühne & Krusat, 1972 [140]. Among these taxa, *Saurodesmus robertsoni* shares with *Bienotherium yunnanense* (FMNH CUP 2285, FMNH CUP 2295), *Tritylodon longaevus*, *Oligokyphus major*, *Eozostrodon parvus*, *Morganucodon watsoni*, the indeterminate morganucodontid PIN 4774/1 from Peski, and *Haldanodon exspectatus* the relatively proximal location of the lesser trochanter. A similar orientation of the lesser trochanter is observed in *Irajatherium hernandezi* Martinelli et al., 2005 [82] and *Therioherpeton cargnini* Bonaparte & Barberena, 1975 [130], but in these taxa it is not separated from the head by a notch. Early cynodonts (i.e., *Thrinaxodon liorhinus* Seeley, 1894 [110], *Galesaurus planiceps* Owen, 1860 [106], *Procynosuchus delaharpeae* Broom, 1937 [109]), most cynognathians (i.e., *Diademodon tetragonus* Seeley, 1894 [114], *Cynognathus crateronotus*, *Cricodon metabolus* Crompton, 1954 [89], *Langbergia modisei* (NMQR 3255), *Luangwa drysdalli*, *Pascualgnathus polanskii* Bonaparte, 1966 [121], *Scalenodon angustifrons* (Parrington, 1946) [122], *Traversodon stahleckeri*, *Massetognathus pascuali* Romer, 1967 [120], *Massetognathus ochagaviae*, *Exaeretodon argentinus* (Cabrera, 1943) [116], *Boreogomphodon jeffersoni*, *Santacruzodon hopsoni*, and *Menadon besairei*) and some probainognathians (i.e., *Chiniquodon theotonicus*, *Prozostrodon brasiliensis*, *Brasilodon quadrangularis*) differ from *Saurodesmus robertsoni* in the mostly ventral orientation of the lesser trochanter.

The ~~preserved region of the~~ greater trochanter of *Saurodesmus robertsoni* is most comparable to

that of the traversodontid *Boreogomphodon jeffersoni* and the tritylodontids *Oligokyphus major*, *Tritylodon longaevus*, and the unnamed taxon CXPM C2019 2A235 from Lufeng, in general outline and relative development. The greater trochanter of *Bienotherium yunnanense* (FMNH CUP 2285, FMNH CUP 2295; possibly ontogenetically variable, because the flaring is less prominent in the small FMNH CUP 2298) and *Bienotheroides wansienensis*, is more laterally flaring than in *Saurodesmus robertsoni*. The greater trochanter of *Saurodesmus robertsoni* is more laterally flaring and has a more expanded base than in early cynodonts (i.e., *Thrinaxodon liorhinus, Galesaurus planiceps, Procynosuchus delaharpeae*). It flares less laterally and is longer proximodistally than in many probainognathians and in most cynognathians, except for *Exaeretodon argentinus*, which shows a comparable proximodistal extension. Unlike *Saurodesmus robertsoni*, and other non-mammalian probainognathians, the greater trochanter is poorly developed in *Irajatherium*. *Saurodesmus robertsoni* lacks a hook-like greater trochanter, differing from the traversodontids *Luangwa drysdalli* and *Traversodon stahleckeri*. Although this character is difficult to assess in many taxa based on the available literature, and may be to some extent influenced by diagenesis, at least the preserved base of the greater trochanter in *Saurodesmus robertsoni* is significantly thinner dorsoventrally (more plate-like) than in *Bienotherium yunnanense*. In the latter taxon, it has a distinctly rhomboid cross-section, regardless of the size/ontogenetic age of the individual (FMNH CUP 2285, FMNH CUP 2295, FMNH 2298). A thinner, plate-like greater trochanter is present, e.g., in *Tritylodon longaevus* (particularly at the base; Fig 9H, I, K), the unnamed taxon CXPM C2019 2A235 from Lufeng, and *Brasilodon quadrangularis* (Fig 9X, Y, A′).

Ventrally, the shallow intertrochanteric and adductor fossae are separated by a faint intertrochanteric crest. This region in *Saurodesmus robertsoni* is most similar to *Thrinaxodon liorhinus* and the unnamed tritylodontid XPM C2019 2A235, and unlike that of most non-mammaliaform cynodonts, which bear a strong intertrochanteric crest (i.e., *Cynognathus crateronotus, Diademodon tetragonus, Luangwa drysdalli, Andescynodon mendozensis, Massetognathus pascuali, Pascualgnathus polanskii, Traversodon stahleckeri, Chiniquodon theotonicus, Probainognathus jenseni*, and *Brasilodon quadrangularis*) or show a single proximoventral fossa (i.e., *Procynosuchus*

delaharpeae, *Galesaurus planiceps*, *Langbergia modisei* (NMQR 3255), *Boreogomphodon jeffersoni*, *Massetognathus ochagaviae*, *Exaeretodon argentinus*, *Santacruzodon hopsoni*, *Menadon besairei*, *Trucidocynodon riograndensis*, *Prozostrodon brasiliensis*, *Bienotherium yunnanense*, *Bienotheroides wansienensis*, *Oligokyphus major*, *Tritylodon longaevus*, the indeterminate tritylodontid MCZ 8838, *Eozostrodon parvus*, *Morganucodon watsoni*, the indeterminate morganucodontid PIN 4774/1 from Peski, and *Haldanodon exspectatus*).

The general outline of the distal region of the femur of *Saurodesmus robertsoni* as observed dorsally is most comparable to *Prozostrodon brasiliensis*, the indeterminate tritylodontid MCZ 8838, *Bienotherium yuannanese*, *Oligokyphus major*, and the unnamed tritylodontid XPM C2019 2A235, but these taxa lack a well-developed medial epicondyle. However, a subtle medial epicondyle is present in the large *Bienotherium yuannanese* FMNH CUP 2285 (Fig 9Q, S, T). In the unnamed tritylodontid XPM C2019 2A235, the lateral condyle is much more projected than in *Saurodesmus robertsoni*. Although this character is not always obvious from in the published literature, at least in *Bienotherium yunnanense* (FMNH CUP 2285; Fig 9Q, V) and PIN 4774/1, as in *Saurodesmus robertsoni*, the distinct dorsal eminence proximal to the medial (tibial) condyle is located close to the level of the long axis of the femoral shaft, that is almost in the middle of the width of the distal expansion.

Ventrally, the medial (tibial) condyle together with the presence of a small, pocket-like fossa proximally to it and walled medially by a sharpened ridge in *Saurodesmus robertsoni* resemble the morphology in the unnamed tritylodontid XPM C2019 2A235. This fossa is also present in *Bienotherium yuannanese* FMNH CUP 2285, *Brasilodon quadrangularis*, and in the indeterminate morganucodontid PIN 4774/1 from Peski, in which it was interpreted as an attachment site for *M. gastrocnemius* [143]. However, unlike in *Saurodesmus robertsoni*, in these latter taxa the medial condyle is less developed lateromedially and relatively smaller when compared to the lateral condyle. Kühne [90] reported a possibly similar fossa in *Oligokyphus major* but the exact morphology is not clear from the published drawings. Compared to *Saurodesmus robertsoni*, the medial and lateral condyles of *Oligokyphus major* are proportionally higher proximodistally and closer to each other. The

fossa and lateral ridge seem to be absent in juvenile individuals of *Brasilodon quadrangularis* and in other non-mammaliaform cynodonts.

~~In distal view, t~~the distal end of *Saurodesmus robertsoni* is overall most similar to *Bienotherium yunnanense* and the Lufeng tritylodontid XPM C2019 2A235, even despite the damage of the latter specimen. However, in *Bienotherium yunnanense* the lateral condyle is more ventrally extended than in *Saurodesmus robertsoni*.

Although we were unable to confirm or refute the presence of a distinct medullary cavity (noted by Seeley [1] and contested by Lydekker's comment published alongside that paper), both possible variants (empty cavity or small medullary region crossed by trabeculae) may occur in cynodont femora [302], making this character inconclusive until a better sampling allowing taxonomic distinctions is obtained.

Overall, the morphology of the femur of *Saurodesmus robertsoni* best corresponds with that in derived, non-mammaliaform prozostrodontians. In particular, it displays morphological traits that, taken together, most closely resemble those observed in tritylodontids, among which it appears to be most comparable to the unnamed specimen from Lufeng (XPM C2019 2A235).

Although each anatomical feature of NHMUK PV OR 28877 fits within the variability observed in cynodont femora, the combination of characters is unique in comparison with other species with known femoral anatomy, allowing to diagnose *Saurodesmus robertsoni* on the basis of its femoral anatomy. ~~Whereas it is possible that the taxonomic validity of *Saurodesmus robertsoni* may be contested in the future, given the current limited record of the postcranial anatomy in cynodonts, it is presently upheld.~~

Saurodesmus robertsoni is the only Triassic cynodont from Scotland. Thus far, the recognized record of Triassic cynodonts in the United Kingdom consisted only of the Mammaliaformes [101,303]: *Hypsiprymnopsis rhaeticus* Dawkins, 1864 [304] (Allotheria) [304,305], *Kuehneotherium* sp. (Kuehneotheriidae) [306], *Morganucodon watsoni* (Morganucodontidae) [138], *Thomasia moorei* (Owen, 1871) [305], *Thomasia antiqua* (Plieninger, 1847) [307], and *Thomasia* sp. (Allotheria)

[305,308] from southern England. Only the hypodigm of *Morganucodon watsoni* includes femora, and the direct comparisons on a more taxonomically broad framework performed above show that *Saurodesmus robertsoni* morphology should be interpreted as less derived. If *Saurodesmus robertsoni* is a tritylodontid, it is one of the earliest representatives of that group. Thus far, only one potential Norian representative of the Tritylodontidae was described from the USA – a distal humerus identified as Tritylodontidae? indet. [309]; however, the subequal development of its radial and ulnar condyles seems to arguably better fit morganucodontids (compare with Jenkins [81]). In the Rhaetian, the record of tritylodontids consists of *Chalepotherium plieningeri* (Ameghino, 1903) [310] and *Oligokyphus triserialis* Hennig, 1922 [311] from Germany [308,311]. The oldest (and, apparently, only) previously identified tritylodontid from Scotland, *Stereognathus hebridicus* Waldman & Savage, 1972 [312], appeared in the Middle Jurassic (middle Bathonian) of the Isle of Skye [312]. Conversely, if *Saurodersmus robertsoni* is a brasilodontid, it would be the youngest member of that family and its only occurrence from the Triassic Northern Hemisphere – outside of the Ladinian–Norian of Brazil, the possible record of the Brasilodontidae thus far is restricted to the Early Triassic of India [79,313–315]. Of course, these interpretations will need to be re-evaluated and validated considering upcoming discoveries of postcranial material of other cynodont groups, as well as possible cranial and dental material of *Saurodesmus robertsoni* that, hopefully, will be obtained in the future.

Conclusions

Saurodesmus robertsoni, a problematic taxon from the Rhaetian of Scotland based on a single specimen (NHMUK PV OR 28877), is here identified as a derived non-mammaliaform cynodont. Despite its incompleteness, the taxon is here considered valid, making it not only one of the historically oldest finds of that group, but also one of the oldest valid cynodont names. That solves the nearly two centuries-long history of controversy concerning the identity of NHMUK PV OR 28877 and has some bearing on the understanding of the spatiotemporal distribution of cynodonts in the latest Triassic.

Acknowledgements

Michael Day (NHMUK) is thanked for access to specimen NHMUK PV OR 28877 and for the help with digitization. Gabriela Cisterna (PULR), Eudald Mujal Grané (SMNS), Rainer Schoch (SMNS), Daniela Schwarz (MB), William Simpson (FMNH CUP), and Heike Straebelow (MB) are thanked for granting access to their respective collections. Bernhard Zipfel and Sifelani Jirah (ESI), and Jennifer Botha, Will Archer, Sekhomotso Gubuza, Masabata Chaka, Confidence Nemudivhiso, Nthaopa Nthari, and Thabang Ntsala (NMQR) are thanked for access and repairs to specimens. Caroline Lam is thanked for the access and agreement for reproduction of Jonathan Stiven's illustration of NHMUK PV OR 28877 preserved in the archives of the Geological Society of London. We thank Fernando Abdala for the helpful comments on the manuscript and photographs of *Bienotherium yuannanese* and *Tritylodon longaevus*. Vincent Fernandez (ESRF – European Synchrotron Radiation Facility, Grenoble, France, and ESI) for the synchrotron scan-based 3D model of the femur of *Thrinaxodon liorhinus* BP/1/7199 (the "odd couple" specimen; [316]); Marco Romano (Sapienza University, Rome, Italy, and ESI) for the photogrammetric 3D model of the femur of *Tapinocaninus pamelae* Rubidge, 1991 [317] NMQR 2987 [298]; Justyna Słowiak-Morkovina (ZPAL) for additional photographs (including photogrammetry) of the femora of *Bienotherium yunnanense* (FMNH CUP 2285 and FMNH CUP 2295). Moreover, Justyna Słowiak-Morkovina and Jolanta Kobylińska (ZPAL) are thanked for the 3D model of the femur of *Ornithorhynchus anatinus* ZPAL z.p. M. 12/13. Guilherme Hermanson and colleagues provided access to the *Trionyx triunguis* humerus and femur and *Chelus fimbriatus* femur 3D data and Michael Stein and colleagues provided access to the *Crocodilus porosus* humerus 3D data originally appearing in Stein *et al.* [75] with data collection funded by DE150100862, DP140102656, DP140102659, DP130100197, DP170101420, and DP180100792. The files were downloaded from www.MorphoSource.org, Duke University. Studies and revisions of Triassic postcranial material attributed to pantestudinates is supported by the National Science Centre, Poland (Narodowe Centrum Nauki) grant 2020/39/B/NZ8/01074 (T. Szczygielski). The support of

GENUS (DSI-NRF Centre of Excellence in Palaeosciences, UID 86073) and the Millenium Trust South Africa towards this research is hereby acknowledged. Opinions expressed and conclusions arrived at, are those of the author and are not necessarily to be attributed to the GENUS.

References

1. Seeley HG. On *Saurodesmus robertsoni* (Seeley), a crocodilian reptile from the Rhaetic of Linksfield, in Elgin. Q J Geol Soc. 1891;47: 166–170.

2. Seeley HG. On *Saurodesmus robertsoni*, a crocodilian reptile from the Rhaetic of Linksfield, in Elgin. Ann Mag Nat Hist Zool Bot Geol. 1891;7: 300.

3. Malcolmson JG. On the occurrence of Wealden strata at Linksfield, near Elgin; on the remains of fishes in the Old Red Sandstone of that neighbourhood; and on raised beaches along the adjacent coast. Proc Geol Soc London. 1838;2: 667–668. doi:10.1144/GSL.JGS.1926.082.01-04.02

4. Malcolmson JG. On the relations of the different parts of the Old Red Sandstone, in which organic remains have recently been discovered, in the counties of Murray, Nairn, Bariff, and Inverness. Proc Geol Soc London. 1839;3: 141–144.

5. Owen R. Report on British fossil reptiles, part II. Reports Br Assoc Adv Sci. 1842;11: 60–204.

6. Duff P. Sketch of the Geology of Moray. Elgin: Forsynth & Young; 1842.

7. Anderson G, Anderson P. Guide to the highlands and islands of Scotland including Orkney and Zetland descriptive of their scenery, statistics, antiquities, and natural history. Containing also directions for visiting the lowlands of Scotland with descriptive notices and maps, vie. Edinburgh: Adam and Charles Black; 1851. Available: https://www.ptonline.com/articles/how-to-get-better-mfi-results

8. Pictet F-J. Traité de paléontologie ou histoire naturelle des animaux fossiles considérés das

leurs rapports zoologiques et géologiques. Seconde édition, revue, corrigée, donsidérablement augumentée. Paris: J.-B. Baillière; 1853.

9. Jones TR. A monograph on the fossil Estheriae. London: Palaeontographical Society; 1862.

10. Maack GA. Die bis jetzt bekannten fossilen Schildkröten und die im oberen Jura bei Kelheim (Bayern) und Hannover neu aufgefundenen ältesten Arten derselben. Palaeontographica. 1869;18: 193–338.

11. Judd JW. The Secondary rocks of Scotland. Q J Geol Soc London. 1873;29: 97–195.

12. Seeley HG. The Dinosauria. Pop Sci Rev. 1880;19: 44–60.

13. Quenstedt FA. Handbuch der Petrefaktenkunde. Tübingen: Verlag der H. Laupp'schen Buchhandlung; 1885. doi:10.5962/bhl.title.30642

14. Lydekker R. Catalogue of the fossil Reptilia and Amphibia in the British Museum (Natural History), Cromwell Road, S.W. Part III. Containing the order Chelonia. London: Order of the Trustees; 1889.

15. Meyer H von. Mittheilung an Professor H. B. Geinitz. Neues Jahrb für Mineral Geol und Paläontologie. 1863;1863: 444–450.

16. Fraas E. *Proterochersis*, eine pleurodire Schildkröte aus dem Keuper. Jahreshefte des Vereins für Vaterländische Naturkd Württemb. 1913;69: 13–30.

17. Szczygielski T. Obscure by name: solving the enigma of *Chelytherium obscurum*, the first described Triassic turtle. Zool J Linn Soc. 2021;192: 1111–1122.

18. International Commission on Zoological Nomenclature. Notice of new applications to the Commission (Case 3858–3862). Bull Zool Nomencl. 2022;78: 2–3. doi:10.21805/bzn.v78.a042

19. Woodward AS, Sherborn CD. A catalogue of British fossil Vertebrata. London: Dulau & Co.; 1890.

20. Seeley HG. Two new reptiles. Am Geol. 1891;8: 56.

21. Seeley HG. VIII. Researches on the structure, organization, and classification of the fossil reptilia.–VII. Further observations on pareiasaurus. Philos Trans R Soc London. 1892;183: 311–370. doi:10.1098/rstb.1892.0008

22. Seeley HG. XIII. Researches on the structure, organisation, and classification of the fossil Reptilia. Part VIII. On further evidences of *Deuterosaurus* and *Rhopalodon* from the Permian rocks of Russia. Proc R Soc London. 1894;54: 168–169. doi:10.1098/rspl.1893.0064

23. Seeley HG. Researches on the structure, organization, and classification of the fossil reptilia. Part VIII.: On further evidences of *Deuterosaurus* and *Rhopalodon* from the Permian rocks of Russia. Ann Br Geol 1893. 1895;1895: 321–322.

24. Seeley HG. On further evidenced of *Deuterosaurus* and *Rhopalodon* from the Permian rocks of Russia. Ann Mag Nat Hist Zool Bot Geol. 1893;12: 74–75.

25. Kammerer CF, Angielczyk KD. A proposed higher taxonomy of anomodont therapsids. Zootaxa. 2009;24: 1–24.

26. Seeley HG. XV. Researches on the structure, organization, and classification of the fossil Reptilia.–VIII. Further evidences of the skeleton in *Deuterosaurus* and *Rhopalosaurus*, from the permian rocks of Russia. Philos Trans R Soc London. 1894;185: 663–717. doi:10.1098/rstb.1894.0015

27. Seeley HG. II. Researches on the structure, organisation and classification of the fossil reptilia.—Part IX., section 5. On the skeleton in new Cynodontia from the Karroo Rocks. Philos Trans R Soc London. 1895;186: 59–148. doi:10.1098/rstb.1894.0021

28. Huene F von. Übersicht über die Reptilien der Trias. Geol und Paläontologische Abhandlungen (Neue Ser. 1902;6: 1–84.

29. Huene F von. Ueber die reptilführenden Sandsteine bei Elgin in Schottland. Cent für Mineral Geol und Paläontologie. 1913;1913: 617–623.

30. Nopcsa FB. Die Familien der Reptilien. Fortschritte der Geologie un Palaeontologie. Berlin:

Verlag von Gebrüder Borntraeger; 1923.

31. Hummel K. Fossilium catalogus I: Animalia. Pars 52: Trionychia fossilia. Quenstedt W, editor. Berlin: W. Junk; 1932.

32. Nopcsa FB. The genera of reptiles. Palaeobiologica. 1928;1: 163–188.

33. Zittel KA von. Text-book of palaeontology. Vol. II. London: MacMillan and co.; 1932.

34. Romer AS. Vertebrate paleontology. Chicago, London: The University of Chicago Press; 1945.

35. Romer AS. Vertebrate paleontology. Third edition. Chicago, London: The University of Chicago Press; 1966.

36. Hay OP. On the group of fossil turtles known as the Amphichelydia; With remarks on the origin and relationships of the suborders, superfamilies, and families of Testudines. Bull Am Museum Nat Hist. 1905;21: 137–175.

37. Bergounioux F-M. Testudinata. In: Piveteau J, editor. Traité de paléontologie Tome V Amphibiens, reptiles, oseaux. Paris: Masson; 1955. pp. 487–544.

38. Huene F von. Paläontologie und Phylogenie der Niederen Tetrapoden. Jena: Gustav Fischer Verlag; 1956. doi:10.2307/1440541

39. Swinnerton HH. Fossils. London: Collins; 1960.

40. Kuhn O. Fossilium catalogus I: Animalia. Pars 107: Testudines. Westphal F, editor. Gravenhage: Uitgeverij Dr W. Junk; 1964.

41. Ingles JM, Sawyer FC. A catalogue of the Richard Owen collection of palaeontological and zoological drawings in the British Museum (Natural History). Bull Br Museum Nat Hist Hist Ser. 1979;6: 109–197.

42. Benton MJ, Walker AD. Palaeoecology, taphonomy, and dating of Permo-Triassic reptiles from Elgin, north-east Scotland. Palaeontology. 1985;28: 207–234.

43. Taylor MA, Cruickshank ARI. A plesiosaur from the Linksfield erratic (Rhaetian, Upper

Triassic) near Elgin, Morayshire. Scottish J Geol. 1993;29: 191–196. doi:10.1144/sjg29020191

44. Storrs GW. Fossil vertebrate faunas of the British Rhaetian (latest Triassic). Zool J Linn Soc. 1994;112: 217–259.

45. Joyce WG. A review of the fossil record of basal Mesozoic turtles. Bull Peabody Museum Nat Hist. 2017;58: 65–113.

46. Teiling E. *Staurodesmus*, genus novum. Containing monospinous desmids. Bot Not. 1948;1: 49–83.

47. Lesiak T. Desmids flora analysis of the peat-bog Lubiec in 1984-1995. Acta Univ Lodz Folia Bot. 2000;15: 227–243.

48. Xenopoulos MA, Frost PC. UV radiation, phosphorus, and their combined effects on the taxonomic composition of phytoplankton in a boreal lake. J Phycol. 2003;39: 291–302. doi:10.1046/j.1529-8817.2003.02125.x

49. Solano Mellizo DC. Cambios temporales y morfologia funcional de la comunidad fitoplanctónica en un lago andino (Laguna de Guatavita, Cundinamarca). Pontificia Universidad Javeriana. 2005.

50. Bicudo CE de M. PEFI algal flora: a half-century of investigation. Hoehnea. 2020;47: e232019. doi:10.1590/2236-8906-23/2019

51. Gontcharov AA. Phylogeny and classification of Zygnematophyceae (Streptophyta): current state of affairs. Fottea. 2008;8: 87–104. doi:10.5507/fot.2008.004

52. Gordon G. Existence of lias on the southern side of the Murray Firth. Proc Geol Soc London. 1833;1: 384. doi:10.1144/GSL.JGS.1926.082.01-04.02

53. Symonds WS. On the physical relations of the reptiliferous sandstone of Elgin. Edinburgh New Philos J. 1860;12: 95–101.

54. Moore C. On the so-called Wealden Beds at Linksfield, and the reptiliferous sandstones of

Elgin. Quaterly J Geol Soc London. 1860;16: 445–447.

55. Anderson FW. Rhaetic Ostracoda. Bull Geol Surv Gt Britain. 1964;21: 133–174.

56. Brickenden L. On the occurrence of the Boulder Clay in the limestone quarry, Linksfield, Elgin, N.B. Quaterly J Geol Soc London. 1851;7: 289–292.

57. Agassiz L. Monographie des poissons fossiles du vieux grès rouge ou Système Dévonien (Old Red Sandstom), des îles Britanniques et de Russie. Soleure: Jent et Gassman; 1844.

58. Taylor MA. Further note on “A plesiosaur from the Linksfield erratic (Rhaetian, Upper Triassic) near Elgin, Morayshare” by M. A. Taylor and A. R. I. Cruickshank (Scottish Journal of Geology 29, 191-6, 1993). Scottish J Geol. 1995;31: 182.

59. Woodward AS. Catalogue of the fossil fishes in the British Museum (Natural History), Cromwell Road, S.W. Part I. Containing the Elasmobranchii. London: Or; 1889. Available: https://www.ptonline.com/articles/how-to-get-better-mfi-results

60. Malcolmson JG. On the relations of the different parts of the Old Red Sandstone in which organic remains have recently been discovered, in the counties of Moray, Nairn, Banff, and Inverness. Quaterly J Geol Soc London. 1859;15: 336–352.

61. Sulej T. The skull of an early Late Triassic aetosaur and the evolution of the stagonolepidid archosaurian reptiles. Zool J Linn Soc. 2010;158: 860–881. doi:10.1111/j.1096-3642.2009.00566.x

62. Young C-C. Preliminary notes on the Lufeng vertebrate fossils. Bull Geol Soc China. 1940;20: 235–240. doi:10.1111/j.1755-6724.1940.mp203-4003.x

63. Abdala F, Neveling J, Welman J. A new trirachodontid cynodont from the lower levels of the Burgersdorp Formation (Lower Triassic) of the Beaufort Group, South Africa and the cladistic relationships of Gondwanan gomphodonts. Zool J Linn Soc. 2006;147: 383–413. doi:10.1111/j.1096-3642.2006.00224.x

64. Broom R. On some new therocephalian reptiles from the Karroo Beds of South Africa. Proc

Zool Soc London. 1920;24: 343–355. doi:10.1111/j.1469-7998.1833.tb06418.x

65. Owen R. On the skull and dentition of a Triassic mammal (*Tritylodon longævus*, Owen) from South Africa. Q J Geol Soc London. 1884;40: 146–152. doi:10.1144/GSL.JGS.1884.40.01-04.07

66. Rougier GW, Fuente MS de la, Arcucci AB. Late Triassic turtles from South America. Science (80-). 1995;268: 855–858. doi:10.1126/science.268.5212.855

67. Szczygielski T, Sulej T. Revision of the Triassic European turtles *Proterochersis* and *Murrhardtia* (Reptilia, Testudinata, Proterochersidae), with the description of new taxa from Poland and Germany. Zool J Linn Soc. 2016;177: 395–427. doi:10.1111/zoj.12374

68. Baur G. Ueber den Ursprung der Extremitäten der Ichthyopterygia. Berichte über Versammlungen des Oberrheinischen Vereines. 1887;20: 17–20.

69. Cope ED. The reptilian order Cotylosauria. Proc Am Philos Soc. 1895;34: 436–457.

70. Shaw G, Nodder FP. The naturalist’s miscellany: or, coloured figures of natural objects; drawn and described immediately from nature. Vol. 10. London: Nodder & Co.; 1799.

71. Dzik J. A beaked herbivorous archosaur with dinosaur affinities from the early late triassic of Poland. J Vertebr Paleontol. 2003;23: 556–574. doi:10.1671/A1097

72. Forskål P. Descriptiones animalium avium, amphibiorum, piscium, insectorum, vermium; quae in itinere orientali observavit. Hauniae: Officina Mölleri, Aulae Typographi; 1775. p. 164. Available: file:///C:/Users/sdegrave/Desktop/shrimp literature/F/Forskal - 1775.pdf

73. Schneider JG. Allgemeine Naturgeschichte der Schildkröten: Nebst einem systemischen Verzeichnisse der einzelnen Arten und zwey Kupfern. Leipzig: J. G. Müller; 1783.

74. Schneider JG. Historiae amphibiorum naturalis et literariae. Fasciculus secundus continens crocodilos, scincos, chamaesauras, boas, pseudoboas, elapes, angues, amphisbaenas et caecilias. Jena: Friedrich Frommann; 1801.

75. Stein MD, Hand SJ, Archer M, Wroe S, Wilson LAB. Quantitatively assessing mekosuchine crocodile locomotion by geometric morphometric and finite element analysis of the forelimb. PeerJ. 2020;8: e9349. doi:10.7717/peerj.9349

76. Cignoni P, Callieri M, Corsini M, Dellepiane M, Ganovelli F, Ranzuglia G. MeshLab: An open-source mesh processing tool. Sixth Eurographics Italian Chapter Conference. 2008. pp. 129–136. doi:10.2312/LocalChapterEvents/ItalChap/ItalianChapConf2008/129-136

77. Vergne R, Pacanowski R, Barla P, Granier X, Schlick C. Radiance scaling for versatile surface enhancement. Proceedings of the 2010 ACM SIGGRAPH symposium on Interactive 3D Graphics and Games. 2010. pp. 143–150.

78. Bonaparte JF, Martinelli AG, Schultz CL, Rogerio R. The sister group of mammals: small cynodonts from the Late Triassic of southern Brazil. Rev Bras Paleontol. 2003;5: 5–27.

79. Guignard ML, Martinelli AG, Soares MB. The postcranial anatomy of *Brasilodon quadrangularis* and the acquisition of mammaliaform traits among non-mammaliaform cynodonts. PLoS One. 2019;14: e0216672. doi:10.1371/journal.pone.0216672

80. Jenkins FA. The postcranial skeleton of African cynodonts. Problems in the early evolution of the mammalian postcranial skeleton. Peabody Museum Nat Hist Bull. 1971;36: 1–216.

81. Jenkins FA, Parrington FR. The postcranial skeletons of the Triassic mammals *Eozostrodon, Megazostrodon* and *Erythrotherium*. Philos Trans R Soc London. 1976;273: 387–431. doi:10.1098/rstb.1976.0022

82. Martinelli AG, Bonaparte JF, Schultz CL, Rubert R. A new tritheledontid (Therapsida, Eucynodontia) from the Late Triassic of Rio Grande do Sul (Brazil) and its phylogenetic relationships among carnivorous non-mammalian eucynodonts. Ameghiniana. 2005;42: 191–208.

83. Sues H-D, Jenkins FA. The postcranial skeleton of *Kayentatherium wellesi* from the of Arizona and the phylogenetic significance of postcranial features in tritylodontid cynodonts. In: Carrano

MT, Gaudin TJ, Blob RW, Wible JR, editors. Amniote paleobiology: perspectives on the evolution of mammals, birds, and reptiles. Chicago: University of Chicago Press; 2006. pp. 114–152.

84. Liu J, Powell J. Osteology of *Andescynodon* (Cynodontia: Traversodontidae) from the Middle Triassic of Argentina. Am Museum Novit. 2009;3674: 1–19. doi:10.1206/606.1

85. Bonaparte JF, Soares MB, Martinelli AG. Discoveries in the Late Triassic of Brazil improve knowledge on the origin of mammals. Hist Nat. 2012;2: 5–30.

86. Gaetano LC, Abdala F, Govender R. The postcranial skeleton of the Lower Jurassic *Tritylodon longaevus* from Southern Africa. Ameghiniana. 2017;54: 1–35. doi:10.5710/AMGH.11.09.2016.3011

87. Guignard ML, Martinelli AG, Soares MB. Reassessment of the postcranial anatomy of *Prozostrodon brasiliensis* and implications for postural evolution of non-mammaliaform cynodonts. J Vertebr Paleontol. 2018;38: 1–18. doi:10.1080/02724634.2018.1511570

88. Gaetano LC, Mocke H, Abdala F. The postcranial anatomy of *Diademodon tetragonus* (Cynodontia, Cynognathia). J Vertebr Paleontol. 2018;38. doi:10.1080/02724634.2018.1451872

89. Crompton AW. On some Triassic cynodonts from Tanganyika. Proc Zool Soc London. 1954;125: 617–669.

90. Kühne WG. The Liassic therapsid *Oligokyphus*. London: British Museum (Natural History); 1956. doi:10.5962/bhl.title.118726

91. de Oliveira TV, Martinelli AG, Soares MB. New information about *Irajatherium hernandezi* Martinelli, Bonaparte, Schultz & Rubert 2005 (Eucynodontia, Tritheledontidae) from the upper triassic (Caturrita Formation, Paraná Basin) of Brazil. Palaontologische Zeitschrift. 2011;85: 67–82. doi:10.1007/s12542-010-0078-5

92. Jenkins FA. The Chañares (Argentina) Triassic reptile fauna. VII. The postcranial skeleton of

the traversodontid *Massetognathus pascuali* (Therapsida, Cynodontia). Breviora. 1970;352: 1–28.

93. Romer AS, Lewis AD. The Chañares (Argentina) Triassic reptile fauna. XIX. Postcranial materials of the cynodonts *Probelesodon* and *Probainognathus*. Breviora. 1973;407: 1–26.

94. Romer AS. The Brazilian Triassic cynodont reptiles *Belesodon* and *Chiniquodon*. Breviora. 1969;332: 1–16.

95. Liu J, Schneider VP, Olsen PE. The postcranial skeleton of *Boreogomphodon* (Cynodontia: Traversodontidae) from the Upper Triassic of North Carolina, USA and the comparison with other traversodontids. PeerJ. 2017;5: e3521. doi:10.7717/peerj.3521

96. Kemp TS. The primitive cynodont *Procynosuchus*: structure, function and evolution of the postcranial skeleton. Philos Trans R Soc London. 1980;288: 217–258.

97. Sun A, Li Y. The postcranial skeleton of the late tritylodont *Bienotheroides*. Vertebr Palasiat. 1985;23: 135–150. Available: http://www.paleoglot.org/files/Sun%26Li_85.pdf

98. Young C-C. Mammal-like reptiles from Lufeng, Yunnan, China. Proc Zool Soc London. 1947;1: 537–597.

99. Bertoni RS. Morfologia pós-craniana de cinodontes *Santacruzodon*, Triássico Médio do Rio Grande do Sul, Bacia do Paraná, Brasil. Universidade Federal do Rio Grande do Sul. 2014.

100. Huene F von. Lieferung 2. Cynodontia. Die Fossilen Reptilien des Südamerikanischen Gondwanalandes Ergebnisse der Sauriergrabungen in Südbrasilien 1928/29. Munich: C. H. Beck'sche Verlagsbuchhandlung; 1936. pp. 83–160.

101. Martin T. Postcranial anatomy of *Haldanodon exspectatus* (Mammalia, Docodonta) from the Late Jurassic (Kimmeridgian) of Portugal and its bearing for mammalian evolution. Zool J Linn Soc. 2005;145: 219–248. doi:10.1111/j.1096-3642.2005.00187.x

102. Parrington FR. On the cynodont genus *Galesaurus*, with a note on the functional significance of the changes in the evolution of the theriodont skull. Ann Mag Nat Hist. 1934;13: 38–67.

103. Pavanatto AEB, Müller RT, Da-Rosa ÁAS, Dias-da-Silva S. New information on the postcranial skeleton of *Massetognathus ochagaviae* Barberena, 1981 (Eucynodontia, Traversodontidae), from the Middle Triassic of Southern Brazil. Hist Biol. 2016;28: 978–989. doi:10.1080/08912963.2015.1070148

104. Kemp TS. Aspects of the structure and functional anatomy of the Middle Triassic cynodont *Luangwa*. J Zool. 1980;191: 193–239. doi:10.1111/j.1469-7998.1980.tb01456.x

105. de Oliveira TV, Soares MB, Schultz CL. *Trucidocynodon riograndensis* gen. nov. et sp. nov. (Eucynodontia), a new cynodont from the Brazilian Upper Triassic (Santa Maria Formation). Zootaxa. 2010;71: 1–71. doi:10.11646/zootaxa.2382.1.1

106. Owen R. On some reptilian fossils from South Africa. Q J Geol Soc London. 1860;16: 49–63.

107. Haughton SH. On Cynodontia from the Middle Beaufort Beds of Harrismith, Orange Free State. Ann Transvaal Museum. 1924;11: 74–92. Available: http://content.ajarchive.org/cgi-bin/showfile.exe?CISOROOT=/00411752&CISOPTR=719%5Cnhttp://hdl.handle.net/10499/AJ2883

108. Butler E, Abdala F, Botha-Brink J. Postcranial morphology of the Early Triassic epicynodont *Galesaurus planiceps* (Owen) from the Karoo Basin, South Africa. Pap Palaeontol. 2019;5: 1–32. doi:10.1002/spp2.1220

109. Broom R. A further contribution to our knowledge of the fossil reptiles of the Karroo. Proc Zool Soc London. 1937;B107: 299–318.

110. Seeley HG. Researches on the structure, organisation, and classification of the fossil Reptilia.—Part IX., Section 1. On the Therosuchia. Philos Trans R Soc London. 1894;185: 987–1018. doi:10.1016/S0140-6736(02)26014-6

111. Bonaparte JF. Dos nuevas '"faunas"' de reptiles triásicos de Argentina. 1st International IUGS Symposium of Gondwana. 1969. pp. 283–306.

112. Sues HD, Olsen PE. Triassic vertebrates of Gondwanan aspect from the Richmond basin of

Virginia. Science (80-). 1990;249: 1020–1023. doi:10.1126/science.249.4972.1020

113. Sues H-D, Hopson JA. Anatomy and phylogenetic relationships of *Boreogomphodon jeffersoni* (Cynodontia: Gomphodontia) from the Upper Triassic of Virginia. J Vertebr Paleontol. 2010;30: 1202–1220. doi:10.1080/02724634.2010.483545

114. Seeley HG. XIII. Researches on the structure, organization, and classification of the fossil Reptilia.—Part IX., Section 3. On *Diademodon*. Philos Trans R Soc London. 1894;185: 1029–1041. doi:10.1098/rstb.1894.0023

115. Broom R. On some points in the anatomy of the theriodont reptile *Diademodon*. Proc Zool Soc London. 1905;1: 96–102. doi:10.1111/j.1469-7998.1833.tb06418.x

116. Cabrera Á. El primer hallazgo de terápsidos en la Argentina. Notas del Mus La Plata. 1943;8: 317–331.

117. Bonaparte JF. Descripción del cráneo y mandíbula de *Exaeretodon frenguellii* Cabrera y su comparación con Diademodontidae, Tritylodontidae y los Cinodontes sudamericanos. Publicaciones del Mus Munic Ciencias Nat y Tradic Mar del Plata. 1962;1: 135–202.

118. Brink AS. Two cynodonts from the Ntawere formation in the Luangwa valley of Northern Rhodesia. Palaeontol Africana. 1963;8: 77–96.

119. Barberena MC. Uma nova espécie de *Massetognathus* (*Massetognathus ochagaviae*, sp.nov.) de formação Santa Maria, Triásico do Rio Grande do Sul. Pesqui em Geociências. 1981;14: 181–195. Available: https://jcr-incites-thomsonreuters.ez54.periodicos.capes.gov.br/JCRJournalHomeAction.action?

120. Romer AS. The Chañares (Argentina) Triassic reptile fauna. III. Two new gomphodonts, *Massetognathus pascuali* and *M. teruggii*. Breviora. 1967;264: 1–23. Available: https://www.ptonline.com/articles/how-to-get-better-mfi-results

121. Bonaparte JF. Sobre nuevos terápsidos triásicos hallados en el centro de la provincia de Mendoza, Argentina. Acta Geol Lilloana. 1966;8: 91–100.

122. Parrington FR. On the cranial anatomy of cynodonts. Proc Zool Soc London. 1946;116: 181–197.

123. Flynn JJ, Parrish JM, Rakotosamimanana B, Ranivoharimanana L, Simpson WF, Wyss AR. New traversodontids (Synapsida: Eucynodontia) from the Triassic of Madagascar. J Vertebr Paleontol. 2000;20: 422–427. doi:10.1671/0272-4634(2000)020[0422:NTSEFT]2.0.CO;2

124. Abdala F, Ribeiro AM. A new traversodontid cynodont from the Santa Maria Formation (Ladinian-Carnian) of southern Brazil, with a phylogenetic analysis of Gondwanan traversodontids. Zool J Linn Soc. 2003;139: 529–545. doi:10.1111/j.1096-3642.2003.00096.x

125. Martinelli AG, Kammerer CF, Melo TP, Paes Neto VD, Ribeiro AM, Da Rosa ÁAS, et al. The African cynodont *Aleodon* (Cynodontia, Probainognathia) in the Triassic of southern Brazil and its biostratigraphic significance. PLoS One. 2017;12: e0177948.

126. Oliveira T, Schultz C, Soares MB. A partial skeleton of *Chiniquodon* (Cynodontia, Chiniquodontidae) from the Brazilian Middle Triassic. Rev Bras Paleontol. 2009;12: 113–122. doi:10.4072/rbp.2009.2.02

127. Mocke HB, Gaetano LC, Abdala F. A new species of the carnivorous cynodont Chiniquodon (Cynodontia, Chiniquodontidae) from the Namibian Triassic. J Vertebr Paleontol. 2020;39: e1754231. doi:10.1080/02724634.2019.1754231

128. Romer AS. The Chañares (Argentina) Triassic reptile fauna. VI. A chiniquodontid cynodont with an incipient squamosal-dentary articulation. Breviora. Cambridge, Mass Museum of Comparative Zoology, Harvard University [1952-; 1970. pp. 1–18.

129. Barbarena MC, Bonaparte JF, Teixeira AMS. *Thrinaxodon brasiliensis* sp. nov., a primeira ocorrência de cinodontes galessauros para o Triássico do Rio Grande do Sul. Anais do X Congresso Brasileiro de Geologia (Rio deJaneiro), Volume 1. 1987. pp. 67–74.

130. Bonaparte JF, Barberena MC. A possible mammalian ancestor from the Middle Triassic of Brazil (Therapsida-Cynodontia). J Paleontol. 1975;49: 931–936.

131. Bonaparte JF, Barberena MC. On two advanced carnivorous cynodonts from the Late Triassic of southern Brazil. Bull Museum Comp Zool. 2001;156: 59–80.

132. de Oliveira TV, Schultz CL. Functional morphology and biomechanics of the cynodont *Trucidocynodon riograndensis* from the Triassic of Southern Brazil: pectoral girdle and forelimb. Acta Palaeontol Pol. 2016;61: 377–386. doi:10.4202/app.00171.2015

133. Patterson B, Olson EC. A triconodont mammal from the Triassic of Yunnan. International Colloquium on the Evolution of Lower and Non Specialized Mammals Koninklijke Vlaamse Academir voor Wetenschappen, Letteren en Schone Kunsten can Belgie. 1961. pp. 129–191.

134. Parrington FR. On the Upper Triassic mammals. Philos Trans R Soc Lond B Biol Sci. 1971;261: 231–272. doi:10.1098/rstb.1971.0055

135. Young C-C. On a *Bienotherium*-like tritylodont from Szechuan, China [in Chinese]. Selected Works of Yang Zhungjian. Beijing: Science Press; 1982. pp. 10–13.

136. Sullivan C, Liu J, Roberts EM, Huang TD, Yang C, Zhong S. Pelvic morphology of a tritylodontid (Synapsida: Eucynodontia) from the Lower Jurassic of China, and some functional and phylogenetic implications. Comptes Rendus - Palevol. 2013;12: 505–518. doi:10.1016/j.crpv.2013.06.008

137. Kermack DM. A new tritylodontid from the Kayenta formation of Arizona. Zool J Linn Soc. 1982;76: 1–17. doi:10.1111/j.1096-3642.1982.tb01953.x

138. Parrington FR. On two mammalian teeth from the lower Rhaetic of Somerset. Ann Mag Nat Hist. 1941;8: 140–144.

139. Crompton AW. A preliminary description of a new mammal from the Upper Triassic of South Africa. J Zool. 1964;142: 441–452.

140. Kühne WG, Krusat G. Legalisierung des Taxon *Haldanodon* (Mammalia, Docodonta). Neues Jahrb für Geol und Paläontologie, Monatshefte. 1972;1972: 300–302.

141. Crompton AW, Jenkins FA. Molar occlusion in Late Triassic mammals. Biol Rev Camb Philos

Soc. 1968;43: 427–458. doi:10.1111/j.1469-185x.1968.tb00966.x

142. Kühne WG. On a triconodont tooth of a new pattern from a fissure-filling in south Glamorgan. Proc Zool Soc London. 1949;119: 345–350.

143. Gambaryan PP, Averianov AO. Femur of a morganucodontid mammal from the Middle Jurassic of Central Russia. Acta Palaeontol Pol. 2001;46: 99–112.

144. Osborn HF. On the primary division of the Reptilia into two sub-classes, Synapsida and Diapsida. Science (80-). 1903;17: 275–276.

145. Broom R. On the use of the term Anomodontia. Rec Albany Museum. 1905;1: 266–269.

146. Owen R. Palaeontology or systematic summary of extinct animals and their geological relations. Second edition. Edinburgh: Adam and Charles Black; 1861.

147. Cope ED. The Tertiary Marsupialia. Naturalist. 1884;18: 686–697.

148. Kutorga S. Zweiter Beitrag zur Geognosie und Paläntologie Dorpat’s und seiner nächsten umgebungen. St. Petersburg: N. Gretsch; 1837.

149. Gaillardot M. Fossil-Reste des Muschelkalkes der Lorraine. Neues Jahrb für Mineral Geognosie, Geol und Petrefaktenkd. 1836;1836: 725–726. Available: http://www.biodiversitylibrary.org/item/111932

150. Bronn HG. Lethaea geognostica, oder Abbildungen und Beschreibungen der für die Gebirge-Formationen bezeichnendsten Versteinerungen. Erster Band, das Übergangs - bis Oolithen-Gebirge enthaltend. Stuttgart: E. Schweizerbart; 1837.

151. Pictet F-J, Humbert A. Description d’une émyde nouvelle (*Emys etalloni*) du terrain Jurassique supérieur des environs de St. Claude. In: Pictet F-J, editor. Matériaux pour la paléontologie suisse, Première série. Geneve: J. Kessmann; 1857. pp. 1–10.

152. Quenstedt FA. *Psammochelys keuperina*. (Ψάμμος Sand, χέλυς Schildkröte.). Jahreshefte des Vereins für Vaterländische Naturkd Württemb. 1889;45: 120–130.

153. Seeley HG. On a fossil reptile with a trunk from the upper Karroo Rocks of Cape Colony. Rep Br Assoc Adv Sci. 1908;1908: 713.

154. Jaekel O. Die Wirbeltierfunde aus dem Keuper von Halberstadt. Serie II. Testudinata. Palaeontol Zeitschrift. 1916;2: 88–214. doi:10.1007/BF03160328

155. Osborn HF. Karl Alfred von Zittel. Science (80-). 1904;19: 186–188.

156. Zittel KA von. Grundzüge der Paläontologie (Paläozoologie). II. Abteilung. Vertebrata. Munich, Berlin: R. Oldenbourg; 1923.

157. Huene F von. Einige Schildkrötenreste aus der obersten Trias Württembergs. Cent für Mineral Geol und Paläontologie Abt B Geol und Paläontologie. 1926;1926: 509–514.

158. Schmidt M. Die Lebenwelt unserer Trias. Oehringen: Hohenlohesche Buchhandlung Ferdinand Rau; 1928.

159. Berckhemer F. C. Geologische Abteilung. Jahreshefte des Vereins für Vaterländische Naturkd Württemb. 1927;83: XXII–XXVII.

160. Berckhemer F. C. Geologische Abteilung. Jahreshefte des Vereins für Vaterländische Naturkd Württemb. 1932;88: XXIV–XXVIII.

161. Berckhemer F. C. Geologische Abteilung. Jahreshefte des Vereins für Vaterländische Naturkd Württemb. 1933;89: XXIII–XXXI.

162. Gaffney ES. The comparative osteology of the Triassic turtle *Proganochelys*. Bull Am Museum Nat Hist. 1990;194: 1–263.

163. Sulej T, Niedźwiedzki G, Bronowicz R. A new Late Triassic vertebrate fauna from Poland with turtles, aetosaurs, and coelophysoid dinosaurs. J Vertebr Paleontol. 2012;32: 1033–1041. doi:10.1080/02724634.2012.694384

164. Szczygielski T, Piechowski R. Limb anatomy of the Triassic turtles: appendicular osteology of *Proterochersis* (Testudinata, Proterochersidae). Zool J Linn Soc. 2023.

165. Li C, Wu X, Rieppel O, Wang L, Zhao L. An ancestral turtle from the Late Triassic of southwestern China. Nature. 2008;456: 497–501. doi:10.1038/nature07533

166. Li C, Fraser NC, Rieppel OC, Xiao-Chun W, Wu XC. A Triassic stem turtle with an edentulous beak. Nature. 2018;560: 476–479. doi:10.1038/s41586-018-0419-1

167. Schoch RR, Sues H-D. A Middle Triassic stem-turtle and the evolution of the turtle body plan. Nature. 2015;523: 584–587. doi:10.1038/nature14472

168. Joyce WG, Lucas SG, Scheyer TM, Heckert AB, Hunt AP. A thin-shelled reptile from the Late Triassic of North America and the origin of the turtle shell. Proc R Soc London B Biol Sci. 2009;276: 507–513. doi:10.1098/rspb.2008.1196

169. Sterli J, Fuente MS de la, Rougier GW. Anatomy and relationships of *Palaeochersis talampayensis*, a Late Triassic turtle from Argentina. Palaeontogr Abteilung A. 2007;281: 1–61.

170. Scheyer TM, Klein N, Evers SW, Mautner A-K, Pabst B. First evidence of *Proganochelys quenstedtii* (Testudinata) from the Plateosaurus bonebeds (Norian, Late Triassic) of Frick, Canton Aargau, Switzerland. Swiss J Palaeontol. 2022;141: 17.

171. Cox CB. The problematic Permian reptile *Eunotosaurus*. Bull Br Museum (Natural Hist Geol. 1969;18: 167–196.

172. Schoch RR, Sues H-D. Osteology of the Middle Triassic stem-turtle *Pappochelys rosinae* and the early evolution of the turtle skeleton. J Syst Palaeontol. 2017; 1–39. doi:10.1080/14772019.2017.1354936

173. Seeley HG. On a new reptile from Welte Vreden (Beaufort West), *Eunotosaurus africanus* (Seeley). Q J Geol Soc. 1892; 583–585.

174. Lyson TR, Rubidge BS, Scheyer TM, de Queiroz K, Schachner ER, Smith RMH, et al. Fossorial origin of the turtle shell. Curr Biol. 2016;26: 1887–1894. doi:10.1016/j.cub.2016.05.020

175. Marzola M. The Late Triassic vertebrate fauna of the Jameson Land Basin, East Greenland: description, phylogeny, and paleoenvironmental implications. Universidade Nova de Lisboa. 2019.

176. Lichtig AJ, Lucas SG. *Chinlechelys* from the Upper Triassic of New Mexico, USA, and the origin of turtles. Palaeontol Electron. 2021;24: a13. doi:10.26879/886

177. Seeley HG. On the primitive reptile *Procolophon*. Proc Zool Soc London. 1905;1: 218–230.

178. Lee MSY. Correlated progression and the origin of turtles. Nature. 1996. pp. 812–815. doi:10.1038/379812a0

179. Gregory WK. Pareiasaurs versus placodonts as near ancestors to the turtles. Bull Am Museum Nat Hist. 1946;86: 279–326.

180. Laurin M, Reisz RR. A reevaluation of early amniote phylogeny. Zool J Linn Soc. 1995;113: 165–223.

181. Lee MSY. Pareiasaur phylogeny and the origin of turtles. Zool J Linn Soc. 1997;120: 197–280. doi:10.1006/zjls.1997.0080

182. DeBraga M, Rieppel OC. Reptile phylogeny and the interrelationships of turtles. Zool J Linn Soc. 1997;120: 281–354. doi:10.1111/j.1096-3642.1997.tb01280.x

183. Romer AS. Osteology of the reptiles. Chicago & London: The University of Chicago Press; 1976. doi:10.1126/science.63.1618.20

184. Lee MSY. A taxonomic revision of pareiasaurian reptiles: implications for Permian terrestrial paleoecology. Modern Geology. 1997. pp. 231–298.

185. Haughton SH, Boonstra LD. Pareiasaurian studies. Part VI.—The osteology and myology of the locomotor apparatus. A.—Hind limb. Ann South African Museum. 1930;28: 297–367.

186. Boonstra LD. Pareiasaurian studies. Part VII.—On the hind limb of the two little-known pareiasaurian genera: *Anthodon* and *Pareiasaurus*. Ann South African Museum. 1932;28: 429–

435.

187. Van den Brandt MJ, Benoit J, Abdala F, Rubidge BS. Postcranial morphology of the South African middle Permian pareiasaurs from the Karoo Basin of South Africa. Palaeontol Africana. 2021;55: 1–91. doi:10.1080/14772019.2022.2035440

188. Turner ML, Tsuji LA, Ide O, Sidor CA. The vertebrate fauna of the upper Permian of Niger—IX. The appendicular skeleton of *Bunostegos akokanensis* (Parareptilia: Pareiasauria). J Vertebr Paleontol. 2015;35: e994746. doi:10.1080/02724634.2014.994746

189. Xu L, Li XW, Jia SH, Liu J. The Jiyuan tetrapod fauna of the upper Permian of China: new pareiasaur material and the reestablishment of *Honania complicidentata*. Acta Palaeontol Pol. 2015;60: 689–700. doi:https://doi.org/10.4202/app.00035.2013

190. Benton MJ. The Chinese pareiasaurs. Zool J Linn Soc. 2016;177: 813–853.

191. Van den Brandt MJ, Abdala F, Benoit J, Day MO, Groenewald GH, Rubidge BS. Taxonomy, phylogeny and stratigraphical ranges of middle Permian pareiasaurs from the Karoo Basin of South Africa. J Syst Palaeontol. 2022;19: 1367–1393. doi:https://doi.org/10.1080/14772019.2022.2035440

192. DeBraga M. The postcranial skeleton, phylogenetic position, and probable lifestyle of the Early Triassic reptile *Procolophon trigoniceps*. Can J Earth Sci. 2003;40: 527–556. doi:10.1139/e02-082

193. Hamley T, Cisneros JC, Damiani R. A procolophonid reptile from the Lower Triassic of Australia. Zool J Linn Soc. 2021;192: 554–609. doi:10.1093/zoolinnean/zlaa056

194. Da Rosa ÁAS, Schwanke C, Aurélio PLP, Poitevin M, Witeck Neto L. Sítio Linha Várzea – uma nova assembléia fossilífera do Triássico Médio do Sul do Brasil. Geociências. 2005;24: 115–129.

195. Cisneros JC, Schultz CL, Da Rosa ÁAS, Schwanke C, Neto LW, Aurélio PLP. Evidência de fenestras temporais em novos materiais de *Candelaria barbouri* Price, 1947 (Parareptilia:

Procolophonia) no Mesotriássico do sul do Brasil: considerações preliminares. In: Gallo V, Brito PM, Silva HMA, Figueiredo FJ, editors. Paleontologia de vertebrados: Grandes temas e contribuções cientificas. Rio de Janeiro: Interciencia; 2006. pp. 219–227.

196. Meckert D. The procolophonid *Barasaurus* and the phylogeny of early amniotes. McGill University. 1995.

197. Mantell GA. Description of the *Telerpeton elginense*, a fossil reptile recently discovered in the Old Red Sandstone of Moray; with observations on supposed fossil ova of batrachians in the Lower Devonian strata of Forfarshire. Quaterly J Geol Soc London. 1852;8: 100–109.

198. Tsuji LA, Müller J. Assembling the history of the Parareptilia: phylogeny, diversification, and a new definition of the clade. Foss Rec. 2009;12: 71–81.

199. Price LI. Two new cotylosaurs from the Permian of Texas. Proc New Engl Zoölogical Club. 1937;16: 97–102.

200. Sumida SS. The appendicular skeleton of the Early Permian genus *Labidosaurus* (Reptilia, Captorhinomorpha, Captorhinidae) and the hind limb musculature of captorhinid reptiles. J Vertebr Paleontol. 1989;9: 295–313. doi:10.1080/02724634.1989.10011764

201. Kissel RA, Dilkes DW, Reisz RR. *Captorhinus magnus*, a new captorhinid (Amniota: Eureptilia) from the Lower Permian of Oklahoma, with new evidence on the homology of the astragalus. Can J Earth Sci. 2002;39: 1363–1372. doi:10.1139/e02-040

202. Holmes R. The osteology and musculature of the pectoral limb of small captorhinids. J Morphol. 1977;152: 101–140. doi:10.1002/jmor.1051520107

203. Clark J, Carroll RL. Romeriid reptiles from the Lower Permian. Bull Museum Comp Zool. 1973;144: 353–407.

204. DeBraga M, Bevitt JJ, Reisz RR. A new captorhinid from the Permian cave system near Richards Spur, Oklahoma, and the taxic diversity of *Captorhinus* at this locality. Front Earth Sci. 2019;7: 112. doi:10.3389/feart.2019.00112

205. Broom R. A new thecodont reptile. Proc Zool Soc London. 1914;1914: 1072–1077. Available: https://zslpublications.onlinelibrary.wiley.com/doi/abs/10.1111/j.1469-7998.1914.tb07729.x

206. Ezcurra MD. The phylogenetic relationships of basal archosauromorphs, with an emphasis on the systematics of proterosuchian archosauriforms. PeerJ. 2016;4: e1778. doi:10.7717/peerj.1778

207. Smith RMH, Evans SE. New material of *Youngina*: evidence of juvenile aggregation in Permian diapsid reptiles. Palaeontology. 1966;39: 289–303.

208. Gow CE. The Morphology and Relationships of *Youngina capensis* Broom and *Prolacerta broomi* Parrington. Palae ont afr. 1975;18: 89–131.

209. Andrews CW. A descriptive catalogue of the marine reptiles of the Oxford Clay, part 1. London: British Museum (Natural History); 1910. doi:10.4324/9780203035993

210. Robin EO. The evolution of plesiosaur and pliosaur morphotypes in the Plesiosauria (Reptilia: Sauropterygia). Paleobiology. 2002;28: 101–112.

211. Klein N, Eggmaier S, Hagdorn H. The redescription of the holotype of *Nothosaurus mirabilis* (Diapsida, Eosauropterygia)—a historical skeleton from the Muschelkalk (Middle Triassic, Anisian) near Bayreuth (southern Germany). PeerJ. 2022;10: e13818. doi:10.7717/peerj.13818

212. Rieppel O. The genus *Placodus*: systematics, morphology, paleobiogeography, and paleobiology. Fieldiana Geol. 1995;31. doi:10.5962/bhl.title.3301

213. Agassiz L. Recherches sur les poissons fossiles. Tome II, contenant l'histoire de l'orde des ganoïdes. Neuchatel: Petitpierre; 1833.

214. Drevermann F. Die Placodontier. 3. Das skelett von *Placodus gigas* Agassiz im Senckenberg-Museum. Abhandlungen der Senckenbergischen Naturforschenden Gesellschaft. 1933. pp. 321–364.

215. Matsumoto R, Dong L, Wang Y, Evans SE. The first record of a nearly complete choristodere (Reptilia: Diapsida) from the Upper Jurassic of Hebei Province, People's Republic of China. J

Syst Palaeontol. 2019;17: 1031–1048. doi:10.1080/14772019.2018.1494220

216. Lü JC, Kobayashi Y, Li ZG. A new species of *Ikechosaurus* (Reptilia: Choristodera) from the Jiufutang Formation (Early Cretaceous) of Chifeng City, Inner Mongolia. Bull l'Institut R des Sci Nat Belqique, Sci la Terre. 1999;69: 37–47.

217. Gao KQ, Ksepka DT. Osteology and taxonomic revision of *Hyphalosaurus* (Diapsida: Choristodera) from the Lower Cretaceous of Liaoning, China. J Anat. 2008;212: 747–768. doi:10.1111/j.1469-7580.2008.00907.x

218. Storrs GW, Gower DJ. The earliest possible choristodere (Diapsida) and gaps in the fossil record of semi-aquatic reptiles. J Geol Soc London. 1993;150: 1103–1107. doi:10.1144/gsjgs.150.6.1103

219. Storrs GW, Large NF. The diapsid reptile *Pachystropheus rhaeticus*, a probable choristodere from the Rhaetian of Europe. Palaeontology. 1996;39: 323–349.

220. Matsumoto R, Evans SE. Choristoderes and the freshwater assemblages of Laurasia. J Iber Geol. 2010;36: 253–274. doi:10.5209/rev

221. Huene F von. Ein Rhynchocephale aus dem Rhät (*Pachystropheus* n.g.). Neues Jahrb für Mineral Geol und Paläontologie. 1935;74: 441–447.

222. Renesto S. A possible find of *Endennasaurus* (Reptilia Thalattosauria), with a comparison between *Endennasaurus* and *Pachystropheus*. Neues Jahrb fur Geol und Palaontologie - Monatshefte. 2005;2005: 118–128. doi:10.1127/njgpm/2005/2005/118

223. Ezcurra MD, Scheyer TM, Butler RJ. The origin and early evolution of Sauria: Reassessing the Permian Saurian fossil record and the timing of the crocodile-lizard divergence. PLoS One. 2014;9. doi:10.1371/journal.pone.0089165

224. Peyer B. Die Triasfauna der Tessiner Kalkalpen. XII. *Macrocnemus bassanii* Nopcsa. Abhandlungen der Schweizerischen Palaeontol Gesellschaft. 1937;59: 1–140.

225. Wild R. Die Triasfauna der Tessiner Kalkalpen. XXIII. *Tanystropheus longobardicus* (Bassani)

(Neue Ergebnisse). Schweizerische Paläontologische Abhandlungen. 1973;95: 1–162.

226. Pritchard AC, Turner AH, Nesbitt SJ, Irmis RB, Smith ND. Late Triassic tanystropheids (Reptilia, Archosauromorpha) from northern New Mexico (Petrified Forest Member, Chinle Formation) and the biogeography, functional morphology, and evolution of Tanystropheidae. J Vertebr Paleontol. 2015;35: 37–41. doi:10.1080/02724634.2014.911186

227. Nesbitt SJ, Stocker MR, Ezcurra MD, Fraser NC, Heckert AB, Parker WG, et al. Widespread azendohsaurids (Archosauromorpha, Allokotosauria) from the Late Triassic of western USA and India. Pap Palaeontol. 2022;8: e1413. doi:10.1002/spp2.1413

228. Chatterjee S. *Malerisaurus langstoni*, a new diapsid reptile from the Triassic of Texas. J Vertebr Paleontol. 1986;6: 297–312. doi:10.1080/02724634.1986.10011627

229. Spielmann JA, Lucas SG, Rinehart LF, Heckert AB. The Late Triassic archosauromorph *Trilophosaurus*. New Mex Museum Nat Hist Sci Bull. 2008;43: 1–177.

230. Nesbitt SJ, Flynn JJ, Pritchard AC, Parrish JM, Ranivoharimanana L, Wyss AR. Postcranial osteology of *Azendohsaurus madagaskarensis* (?Middle to Upper Triassic, Isalo Group, Madagascar) and its systematic position among stem archosaur reptiles. Bull Am Museum Nat Hist. 2015;398: 1–126. doi:10.1206/amnb-899-00-1-126.1

231. Case EC. A cotylosaur from the Upper Triassic of western Texas. J Washingt Acad Sci. 1928;18: 177–178.

232. Flynn JJ, Nesbitt SJ, Michael Parrish J, Ranivoharimanana L, Wyss AR. A new species of *Azendohsaurus* (Diapsida: Archosauromorpha) from the Triassic Isalo Group of southwestern Madagascar: Cranium and mandible. Palaeontology. 2010;53: 669–688. doi:10.1111/j.1475-4983.2010.00954.x

233. Huxley TH. On *Hyperodapedon*. Q J Geol Soc London. 1869;25: 138–152.

234. Benton MJ. The Triassic reptile *Hyperodapedon* from Elgin: functional morphology and relationships. Philos Trans R Soc London. 1983;302: 605–720.

235. Murchinson RI. On the sandstones of Morayshire (Elgin, &c.) containing reptilian remains; and on their relations to the Old Red Sandstone. Quaterly J Geol Soc London. 1859;15: 419–136.

236. Montefeltro FC, Bittencourt JS, Langer MC, Schultz CL. Postcranial anatomy of the hyperodapedontine rhynchosaur *Teyumbaita sulcognathus* (Azevedo and Schultz, 1987) from the Late Triassic of Southern Brazil. J Vertebr Paleontol. 2013;33: 67–84. doi:10.1080/02724634.2012.710285

237. Mukherjee D, Ray S. A new *Hyperodapedon* (Archosauromorpha, Rhynchosauria) from the Upper Triassic of India: implications for rhynchosaur phylogeny. Palaeontology. 2015;57: 1241–1276. doi:10.1111/pala.12113

238. Sookias RB, Butler RJ. Euparkeriidae. Geol Soc Spec Publ. 2013;379: 35–48. doi:10.1144/SP379.6

239. Demuth OE, Rayfield EJ, Hutchinson JR. 3D hindlimb joint mobility of the stem-archosaur Euparkeria capensis with implications for postural evolution within Archosauria. Sci Rep. 2020;10: 1–14. doi:10.1038/s41598-020-70175-y

240. Borsuk-Białynicka M, Sennikov AG. Archosauriform postcranial remains from the early Triassic karst deposits of southern Poland. Palaeontol Pol. 2009;65: 283–328.

241. Trotteyn MJ, Arcucci AB, Raugust T. Proterochampsia: An endemic archosauriform clade from South America. Geol Soc Spec Publ. 2013;379: 59–90. doi:10.1144/SP379.23

242. Müller RT, Garcia MS, Fonseca A de O. A new proterochampsid (Archosauriformes: Proterochampsia) from the Late Triassic of southern Brazil and the emergence of archosaurian hind limb traits. J Syst Palaeontol. 2022;20: 1–19. doi:10.1080/14772019.2022.2128913

243. Trotteyn MJ. Material Postcraneano de *Proterochampsa barrionuevoi* Reig 1959 (Diapsida: Archosauriformes) del Triásico Superior del Centro-oeste de Argentina. Ameghiniana. 2011;48: 424–446. doi:10.5710/AMGH.v48i4(351)CITATIONS

244. Trotteyn MJ, Martínez RN, Alcober OA. A new proterochampsid *Chanaresuchus*

ischigualastensis (Diapsida, Archosauriformes) in the early Late Triassic Ischigualasto Formation, Argentina. J Vertebr Paleontol. 2012;32: 485–489. doi:10.1080/02724634.2012.645975

245. Arcucci AB. Un nuevo Proterochampsidae (Reptilia–Archosauriformes) de la fauna local de Los Chanares (Triasico Medio), La Rioja, Argentina. Ameghiniana. 1990;27: 365–378.

246. Desojo JB, Ezcurra MD, Schultz CL. An unusual new archosauriform from the Middle-Late Triassic of southern Brazil and the monophyly of Doswelliidae. Zool J Linn Soc. 2011;161: 839–871. doi:10.1111/j.1096-3642.2010.00655.x

247. Weems RE. An unusual uewly discovered archosaur from the Upper Triassic of Virginia, U.S.A. Trans Am Philos Soc. 1980;70: 1. doi:10.2307/1006472

248. Heckert AB, Lucas SG, Spielmann JA. A new species of the enigmatic archosauromorph *Doswellia* from the Upper Triassic Bluewater Creek Formation, New Mexico, USA. Palaeontology. 2012;55: 1333–1348. doi:10.1111/j.1475-4983.2012.01200.x

249. Sues HD, Desojo JB, Ezcurra MD. Doswelliidae: A clade of unusual armoured archosauriforms from the Middle and Late Triassic. Geol Soc Spec Publ. 2013;379: 49–58. doi:10.1144/SP379.13

250. Dilkes D, Sues HD. Redescription and phylogenetic relationships of *Doswellia kaltenbachi* (diapsida: Archosauriformes) from the Upper Triassic of Virginia. J Vertebr Paleontol. 2009;29: 58–79. doi:10.1080/02724634.2009.10010362

251. Long RA, Murry PA. Late Triassic (Carnian and Norian) tetrapods from the southwestern United States. New Mex Museum Nat Hist Sci Bull. 1995;4: 1–254.

252. Wynd BM, Nesbitt SJ, Stocker MR, Heckert AB. A detailed description of *Rugarhynchos sixmilensis*, gen. et comb. nov. (Archosauriformes, Proterochampsia), and cranial convergence in snout elongation across stem and crown archosaurs. J Vertebr Paleontol. 2019;39: e1748042. doi:10.1080/02724634.2019.1748042

253. Sookias RB. The relationships of the Euparkeriidae and the rise of Archosauria. R Soc Open Sci. 2016;3. doi:10.1098/rsos.150674

254. Nesbitt SJ, Stocker MR, Small BJ, Downs A. The osteology and relationships of *Vancleavea campi* (Reptilia: Archosauriformes). Zool J Linn Soc. 2009;157: 814–864. doi:10.1111/j.1096-3642.2009.00530.x

255. Ezcurra MD, Butler RJ, Gower DJ. "Proterosuchia": The origin and early history of archosauriformes. Geol Soc Spec Publ. 2013;379: 9–33. doi:10.1144/SP379.11

256. Colbert EH. A gliding reptile from the Triassic of New Jersey. Am Museum Novit. 1966; 1–23.

257. Colbert EH. The Triassic Gliding Reptile: *Icarosaurus*. Bull Am Museum Nat Hist. 1970;143: 85–142.

258. Robinson PL. Gliding lizards from the Upper Keuper of Great Britain. Proc Geol Soc London. 1962;1601: 137–146.

259. Stein K, Palmer C, Gill PG, Benton MJ. The aerodynamics of the British Late Triassic Kuehneosauridae. Palaeontology. 2008;51: 967–981. doi:10.1111/j.1475-4983.2008.00783.x

260. Owen R. On the fossils obtained by the Marchioness of Hastings from the freshwater Eocene beds of the Hordle Cliffs. Rep Br Assoc Adv Sci. 1848; 65–66.

261. Wood S. On the discovery of an alligator and of several new Mammalia in the Hordwell Cliff; with observations upon the geological phenomena of that locality. London Geol Journal, Rec Discov Br Foreign Paleontol. 1846;1: 1–7.

262. Rio JP, Mannion PD, Tschopp E, Martin JE, Delfino M. Reappraisal of the morphology and phylogenetic relationships of the alligatoroid crocodilian *Diplocynodon hantoniensis* from the late Eocene of the United Kingdom. Zool J Linn Soc. 2020;188: 653. doi:10.1093/zoolinnean/zlz075

263. Weinbaum JC. Postcranial skeleton of *Postosuchus kirkpatricki* (Archosauria: Paracrocodylomorpha), from the upper Triassic of the United States. Geol Soc Spec Publ.

2013;379: 525–553. doi:10.1144/SP379.7

264. Schachner ER, Irmis RB, Huttenlocker AK, Sanders K, Cieri RL, Nesbitt SJ. Osteology of the Late Triassic bipedal archosaur *Poposaurus gracilis* (Archosauria: Pseudosuchia) from western North America. Anat Rec. 2020;303: 874–917. doi:10.1002/ar.24298

265. Chatterjee S. A primitive parasuchid (phytosaur) reptile from the Upper Triasic Maleri fromation of India. Palaeontology. 1978;21: 83–127. doi:10.1098/rstb.1974.0001

266. Walker AD. Triassic reptiles from the Elgin area: *Stagonolepis*, *Dasygnathus* and their allies. Philos Trans R Soc London. 1961;244: 103–204.

267. Dróżdż D. Osteology of a forelimb of an aetosaur *Stagonolepis olenkae* (Archosauria: Pseudosuchia: Aetosauria) from the Krasiejów locality in Poland and its probable adaptations for a scratch-digging behavior. PeerJ. 2018;6: e5595. doi:10.7717/peerj.5595

268. Zeigler KE, Heckert AB, Lucas SG. An illustrated atlas of the phytosaur (Archosauria: Parasuchidae) postcrania from the Upper Triassic Snyder Quarry (Petrified Forest Formation, Chinle Group). New Mex Museum Nat Hist Sci Bull. 2003;24: 81–88.

269. Piechowski R, Tałanda M. The locomotor musculature and posture of the early dinosauriform *Silesaurus opolensis* provides a new look into the evolution of Dinosauromorpha. J Anat. 2020;236: 1044–1100. doi:10.1111/joa.13155

270. Meers MB. Crocodylian forelimb musculature and its relevance to Archosauria. Anat Rec. 2003;274: 891–916. doi:10.1002/ar.a.10097

271. Nesbitt SJ. The early evolution of archosaurs: relationships and the origin of major clades. Bull Am Museum Nat Hist. 2011;352: 1–292. doi:10.1206/352.1

272. Huxley TH. The crocodilian remains found in the Elgin Sandstones, with remarks on the ichnites of Cummingstone. London: HM Stationery Office; 1877.

273. Huxley TH. On the *Stagonolepis robertsoni* (Agassiz) of the Elgin Sandstones; and on the recently discovered footmarks in the sandstones of Cummingstone. Quaterly J Geol Soc

London. 1859;15: 440–460.

274. Huxley TH. On *Stagonolepis robertsoni*, and on the evolution of the Crocodilia. Q J Geol Soc London. 1875;31: 423–438. doi:10.1144/GSL.JGS.1875.031.01-04.29

275. Meyer H von. Reptilien aus dem Stubensandstein des oberen Keupers. Palaeontographica. 1861; 301–346.

276. Long RA, Ballew KL. Aetosaur dermal armor from the Late Triassic of Southwestern North America, with special reference to material from Chinle Formation of Petrified Forest National Park. Museum North Arizona Bull. 1985;54: 45–68.

277. Meyer C von. Fossile Knochen aus dem Keuper. In: Meyer CH von, Plininger T, editors. Plininger Beiträge zur Paläontologie Wüttemberg's, enthaltend die fossilen Wirbeltierreste aus dem Triasgebilden mit besonderer Rücksicht auf die Labyrinthodonten des Keupers. Stuttgart: E. Schweizerbart'sche Verlagsbuchhandlung; 1844. pp. 1–45.

278. Stecher R. A new Triassic pterosaur from Switzerland (Central Austroalpine, Grisons), *Raeticodactylus filisurensis* gen. et sp. nov. Swiss J Geosci. 2008;101: 185–201. doi:10.1007/s00015-008-1252-6

279. Padian K. A functional analysis of flying and walking in pterosaurs. Palaeobiology. 1983;9: 218–239.

280. Kellner AWA. Comments on triassic pterosaurs with discussion about ontogeny and description of new taxa. An Acad Bras Cienc. 2015;87: 669–689. doi:10.1590/0001-3765201520150307

281. Dalla Vecchia FM. *Seazzadactylus venieri* gen. et sp. nov., a new pterosaur (Diapsida: Pterosauria) from the Upper Triassic (Norian) of northeastern Italy. PeerJ. 2019;7: e7363. doi:10.7717/peerj.7363

282. Martínez RN, Apaldetti C, Correa GA, Abelín DO. A norian lagerpetid dinosauromorph from the Quebrada del Barro Formation, northwestern Argentina. Ameghiniana. 2016;53: 1–13.

doi:10.5710/AMGH.21.06.2015.2894

283. McCabe MB, Nesbitt SJ. The first pectoral and forelimb material assigned to the lagerpetid *Lagerpeton chanarensis* (Archosauria: Dinosauromorpha) from the upper portion of the Chañares Formation, Late Triassic. Palaeodiversity. 2021;14. doi:10.18476/pale.v14.a5

284. Kellner AWA, Holgado B, Grillo O, Pretto FA, Kerber L, Pinheiro FL, et al. Reassessment of *Faxinalipterus minimus*, a purported Triassic pterosaur from southern Brazil with the description of a new taxon. PeerJ. 2022;10: 1–32. doi:10.7717/peerj.13276

285. Martz JW, Small BJ. Non-dinosaurian dinosauromorphs from the Chinle Formation (Upper Triassic) of the Eagle Basin, northern Colorado: *Dromomeron romeri* (Lagerpetidae) and a new taxon, *Kwanasaurus williamparkeri* (Silesauridae). PeerJ. 2019;7: e7551. doi:10.7717/peerj.7551

286. Colbert EH. The Triassic dinosaur *Coelophysis*. Museum North Arizona Bull. 1989;57: 160.

287. Novas FE, Agnolin FL, Ezcurra MD, Temp Müller R, Martinelli AG, Langer MC. Review of the fossil record of early dinosaurs from South America, and its phylogenetic implications. J South Am Earth Sci. 2021;110: 103341. doi:10.1016/j.jsames.2021.103341

288. Nau D, Lallensack JN, Bachmann U, Sander PM. Postcranial osteology of the first early-stage juvenile skeleton of *Plateosaurus trossingensis* from the Norian of Frick, Switzerland. Acta Palaeontol Pol. 2020;65: 679–708. doi:10.4202/APP.00757.2020

289. Datta PM, Ray S. Earliest lizard from the Late Triassic (Carnian) of India. J Vertebr Paleontol. 2006;26: 795–800.

290. Conrad JL. Phylogeny and systematics of Squamata (Reptilia) based on morphology. Bull Am Museum Nat Hist. 2008;310: 1–182. doi:10.1206/310.1

291. Simões TR, Caldwell MW, Tałanda M, Bernardi M, Palci A, Vernygora O, et al. The origin of squamates revealed by a Middle Triassic lizard from the Italian Alps. Nature. 2018;557: 706–709. doi:10.1038/s41586-018-0093-3

292. Renesto S, Posenato R. A new lepidosauromorph reptile from the Middle Triassic of the Dolomites (northern Italy). Riv Ital di Paleontol e Stratigr. 2003;109: 463–374.

293. Fraser NC, Walkden GM. The postcranial skeleton of the Upper Triassic sphenodontid *Planocephalosaurus robinsonae*. Palaeontology. 1984;27: 575–595.

294. Seeley HG. Note on a femur and humerus of a small mammal from the Stonesfield Slate. Quaterly J Geol Soc London. 1879;35: 456–463.

295. Sauvage MHE. Recherches sur les reptiles trouvés dans l'étage Rhétien des environs d'Autun. Ann des Sci Géologiques. 1883;14: 1–44. Available: https://gallica.bnf.fr/ark:/12148/bpt6k432579f

296. Fischer V, Cappetta H, Vincent P, Garcia G, Goolaerts S, Martin JE, et al. Ichthyosaurs from the French Rhaetian indicate a severe turnover across the Triassic–Jurassic boundary. Naturwissenschaften. 2014;101: 1027–1040. doi:10.1007/s00114-014-1242-7

297. Rieppel O. Handbuch der Paläoherpetologie. Encyclopedia of paleoherpetology. Teil 12A/Part 12A. Sauropterygia I. Placodontia, Pachypleurosauria, Nothosauroidea, Pistosauroidea. Wellnhofer P, editor. Munich: Verlag Dr. Friedrich Pfeil; 2000.

298. Rubidge BS, Govender R, Romano M. The postcranial skeleton of the basal tapinocephalid dinocephalian *Tapinocaninus pamelae* (Synapsida: Therapsida) from the South African Karoo Supergroup. J Syst Palaeontol. 2019;17: 1547–1569. doi:10.1080/14772019.2018.1559244

299. Boonstra LD. The girdles and limbs of the South African Deinocephalia. Ann South African Museum. 1855;42: 185–326.

300. Gregory WK, Camp CL. Studies in comparative myology and osteology. No. III. Bull Am Museum Nat Hist. 1918;38: 447–563. Available: http://digitallibrary.amnh.org/dspace/handle/2246/1061?mode=full&submit_simple=More+bibliographic+detail+

301. Fourie H, Rubidge BS. The postcranial skeleton of the basal therocephalian *Glanosuchus*

macrops (Scylacosauridae) and comparison of morphological and phylogenetic trends amongst the Theriodontia. Palaeontol Africana. 2009;44: 27–39.

302. Botha-Brink J, Soares MB, Martinelli AG. Osteohistology of Late Triassic prozostrodontian cynodonts from Brazil. PeerJ. 2018;6: e5029. doi:10.7717/peerj.5029

303. Bi S, Zheng X, Meng J, Wang X, Robinson N, Davis B. A new symmetrodont mammal (Trechnotheria: Zhangheotheriidae) from the Early Cretaceous of China and trechnotherian character evolution. Sci Rep. 2016;6: 26668. doi:10.1038/srep26668

304. Dawkins B. On the Rhaetic Beds and White Lias of western and central Somerset; and on the discovery of a new fossil mammal in the Grey Marlstones beneath the bone-bed. Q J Geol Soc. 1864;20: 396–412.

305. Owen R. Monograph of the fossil Mammalia of the Mesozoic formations. London: Palaeontographical Society; 1871.

306. Fraser NC, Walkden GM, Stewart V. The first pre-Rhaetic therian mammal. Nature. 1985;314: 161–163. doi:10.1038/315279a0

307. Plieninger T. Abbildungen von Zähnen aus der oberen Grenzbreccie des Keupers bei Degerloch und Stainenbronn vor, mit folgenden Bemerkungen. Jahreshefte des Vereins für Vaterländische Naturkd Württemb. 1847;3: 164–167. Available: http://www.biodiversitylibrary.org/item/35802

308. Simpson GG. A catalogue of the Mesozoic Mammalia in the Geological Department of the British Museum. London: Order of the Trustees; 1928.

309. Zeigler KE, Heckert AB, Lucas SG. The vertebrate fauna of the Upper Triassic (Revueltian) Snyder Quarry. New Mex Museum Nat Hist Sci Bull. 2003;24: 71–79. Available: http://libres.uncg.edu/ir/asu/f/Heckert_A_2003_24_The_vertebrate_fauna.pdf

310. Ameghino F. Los diprotodontes del orden de los plagiaulacoideos. An del Mus Nac Buenos Aires. 1903;9: 81-.

311. Hennig E. Die Säugerzähne des württembergischen Rhät-Lias-Bonebeds. Neues Jahrb für Mineral Geol und Paläontologie. 1922;46: 181–267.

312. Waldman M, Savage RJG. The first Jurassic mammal from Scotland. J Geol Soc London. 1972;128: 119–125. doi:10.1144/gsjgs.128.2.0119

313. Das P, Gupta A. New cynodont record from the Lower Triassic Panchet Formation, Damodar Valley. J Geol Soc India. 2012;79: 175–180. doi:10.1007/s12594-012-0022-2

314. Bonaparte JF. Evolution of the Brasilodontidae (Cynodontia-Eucynodontia). Hist Biol. 2013;25: 643–653. doi:10.1080/08912963.2012.731154

315. Bonaparte JF, Soares MB, Schultz CL. A new non-mammalian cynodont from the Middle Triassic of southern Brazil and its implications for the ancestry of mammals. New Mex Museum Nat Hist Sci Bull. 2006;37: 599–607.

316. Fernandez V, Abdala F, Carlson KJ, Cook DC, Rubidge BS, Yates A, et al. Synchrotron reveals Early Triassic odd couple: injured amphibian and aestivating therapsid share burrow. PLoS One. 2013;8: e64978. doi:10.1371/journal.pone.0064978

317. Rubidge BS. A new primitive dinocephalian mammal-like reptile from the Permian of southern Africa. Palaeontology. 1991;34: 547–559.

Figure captions

Fig 1. *Saurodersmus robertsoni* NHMUK PV OR 28877, right femur and associated rock fragment before they were separated. “Drawn by J. Stiven, from a specimen found at Linksfield by Alex^{r}. Robertson Esqr. Inverugie F. G. S. Elgin 1840”. Watercolour. Note the bone is figured upside-down (proximal end towards the bottom of the page). Reproduced by permission of the Geological Society of London.

Fig 2. *Saurodersmus robertsoni* NHMUK PV OR 28877, right femur (A–B, D–I) and associated

rock fragment with natural mould of the posterior surface of the bone (A, C) in anterior (A, C, D), posterior (B, F), lateral (E), medial (G), proximal (H), and distal (I) view.

Fig 3. Comparison of *Saurodersmus robertsoni* NHMUK PV OR 28877 (A–F) with humeri of turtles. G–L, *Proterochersis porebensis* ZPAL V. 39/156 (Proterochersidae, right, juvenile); M–R, *Proterochersis porebensis* ZPAL V. 39/50 (Proterochersidae, right, adult); S–X, *Proganochelys quenstedti* SMNS 17203 (Proganochelyidae, left mirrored to right, juvenile); Y–D′, *Proganochelys quenstedti* SMNS 16980 (Proganochelyidae, right, subadult); E′–J′, *Palaeochersis talampayensis* PULR 068 (Australochelyidae, right); K′–P′, *Trionyx triunguis* UF H65522 (Cryptodira: Trionychidae, right). A, G, M, S, Y, E′, K′, dorsal view; B, H, N, T, Z, F′, L′, medial (posterior) view; C, I, O, U, A′, G′, M′, ventral view; D, J, P, V, B′, H′, N′, lateral (anterior) view; E, K, Q, W, C′, I′, O′, proximal view; F, L, R, X, D′, J′, P′, distal view. Ectepicondylar grooves/foramina indicated by red arrowheads. Not to scale.

Fig 4. Comparison of *Saurodersmus robertsoni* NHMUK PV OR 28877 (A–F) with femora of turtles. G–L, *Proterochersis porebensis* ZPAL V. 39/432 (Proterochersidae, right, Late Triassic); M–R, *Proganochelys quenstedti* SMNS 17203 (Proganochelyidae, left mirrored to right, juvenile, Late Triassic); S–X, *Proganochelys quenstedti* SMNS 16980 (Proganochelyidae, left mirrored to right, subadult, Late Triassic); Y–D′, *Palaeochersis talampayensis* PULR 068 (Australochelyidae, left mirrored to right, Late Triassic); E′–J′, *Trionyx triunguis* UF H65522 (Cryptodira: Trionychidae, right, extant); K′–P′, *Chelus fimbriatus* UF H85199 (Pleurodira: Chelidae, right, extant). A, G, M, S, Y, E′, K′, dorsal view; B, H, N, T, Z, F′, L′, medial (posterior) view; C, I, O, U, A′, G′, M′, ventral view; D, J, P, V, B′, H′, N′, lateral (anterior) view; E, K, Q, W, C′, I′, O′, proximal view; F, L, R, X, D′, J′, P′, distal view. Not to scale.

Fig 5. Comparison of *Saurodersmus robertsoni* NHMUK PV OR 28877 (A–F) with femora of non-diapsid reptiles. G–L, *Bradysaurus baini* NHMUK PV R 4066 (Pareiasauria, right, middle Permian); M–R, *Labidosaurus hamatus* MB.R.4433 (Captorhinidae, right, early Permian, cast); A, G, M, anterior view; B, H, N, lateral view; C, I, O, posterior view; D, J, P, medial view; E, K, Q, proximal view; F, L, R, distal view. Not to scale.

Fig 6. Comparison of *Saurodersmus robertsoni* NHMUK PV OR 28877 (A–F) with humeri of archosaurs. G–L, *Crocodylus porosus* Laboratory of Stephen Wroe uncat. (Crocodylia, right, extant); M–R, *Stagonolepis olenkae* ZPAL Ab. III/1175 (Aetosauria, right, Late Triassic); S–X, *Parasuchus* sp. ZPAL Ab. III/1994 (Phytosauria, right, Late Triassic); X–C′, *Silesaurus opolensis* ZPAL Ab. III/452 (Dinosauromorpha, left mirrored to right, Late Triassic). A, G, M, S, X, dorsal view; B, H, N, T, Y, medial view; C, I, O, U, Z, ventral view; D, J, P, V, A′, lateral view; E, K, Q, W, B′, proximal view; F, L, R, X, C′, distal view. Ectepicondylar groove indicated by red arrowheads. Not to scale.

Fig 7. Comparison of *Saurodersmus robertsoni* NHMUK PV OR 28877 (A–F) with femora of non-cynodontian synapsids. G–L, *Tapinocaninus pamelae* NMQR 2987 (Dinocephalia, left mirrored to right, late Permian); M–R, *Moschorhinus kitchingi* NMQR 3939 (Therocephalia, left mirrored to right, late Permian to Early Triassic). A, G, M, anterior view; B, H, N, lateral view; C, I, O, posterior view; D, J, P, medial view; E, K, Q, proximal view; F, L, R, distal view. Not to scale.

Fig 8. Comparison of *Saurodersmus robertsoni* NHMUK PV OR 28877 (A–F) with femora of non-mammaliamorph cynodonts. G–L, *Thrinaxodon liorhinus* BP/1/7199 (non-eucynodont Epicynodontia, right, Early Triassic); M–R, *Cynognathus crateronotus* NMQR 1206 (Eucynodontia: stem Cynognathia, right, Middle to Late Triassic); S–X, Cynognathia indet. (*Diademodon* sp. or *Cynognathus* sp.) BP/1/1675 (Eucynodontia: stem Cynognathia, right, Middle to Late Triassic); Y-D′, *Langbergia modisei* NMQR 3255 (Cynognathia: Trirachodontidae). A, G, M, S, Y, anterior view; B, H, N, T, Z, lateral view; C, I, O, U, A′, posterior view; D, J, P, V, B′, medial view; E, K, Q, W, C′, proximal view; F, L, R, X, D′, distal view. Not to scale. Note the variable development of the hooked greater trochanter in cynognathians.

Fig 9. Comparison of *Saurodersmus robertsoni* NHMUK PV OR 28877 (A–F) with femora of mammaliamorph cynodonts. G–P, *Tritylodon longaevus* BP/1/5089 (G–K, proximal end) and BP/1/4783 (L–P, distal end) (Tritylodontidae, left mirrored to right, Early Jurassic, casts); Q–V, *Bienotherium yunnanense* FMNH CUP 2285 (Tritylodontidae, left mirrored to right, Early Jurassic); W–B′, *Brasilodon quadrangularis* UFRGS-PV-1043-T (Brasilodontidae, right, Late Triassic; modified from Guignard *et al.* [79]); C′–H′, *Ornithorhynchus anatinus* ZPAL z.p. M. 12/13

(Mammalia: Monotremata, left mirrored to right, extant). A, G, L, Q, W, C′, anterior view; B, H, M, R, X, D′, lateral view; C, I, N, S, Y, E′, posterior view; D, J, O, T, Z, F′, medial view; E, K, U, A′, G′, proximal view; F, P, V, B′, H′, distal view. Not to scale.

Figure 1

Click here to access/download;Figure;Fig 1.tif

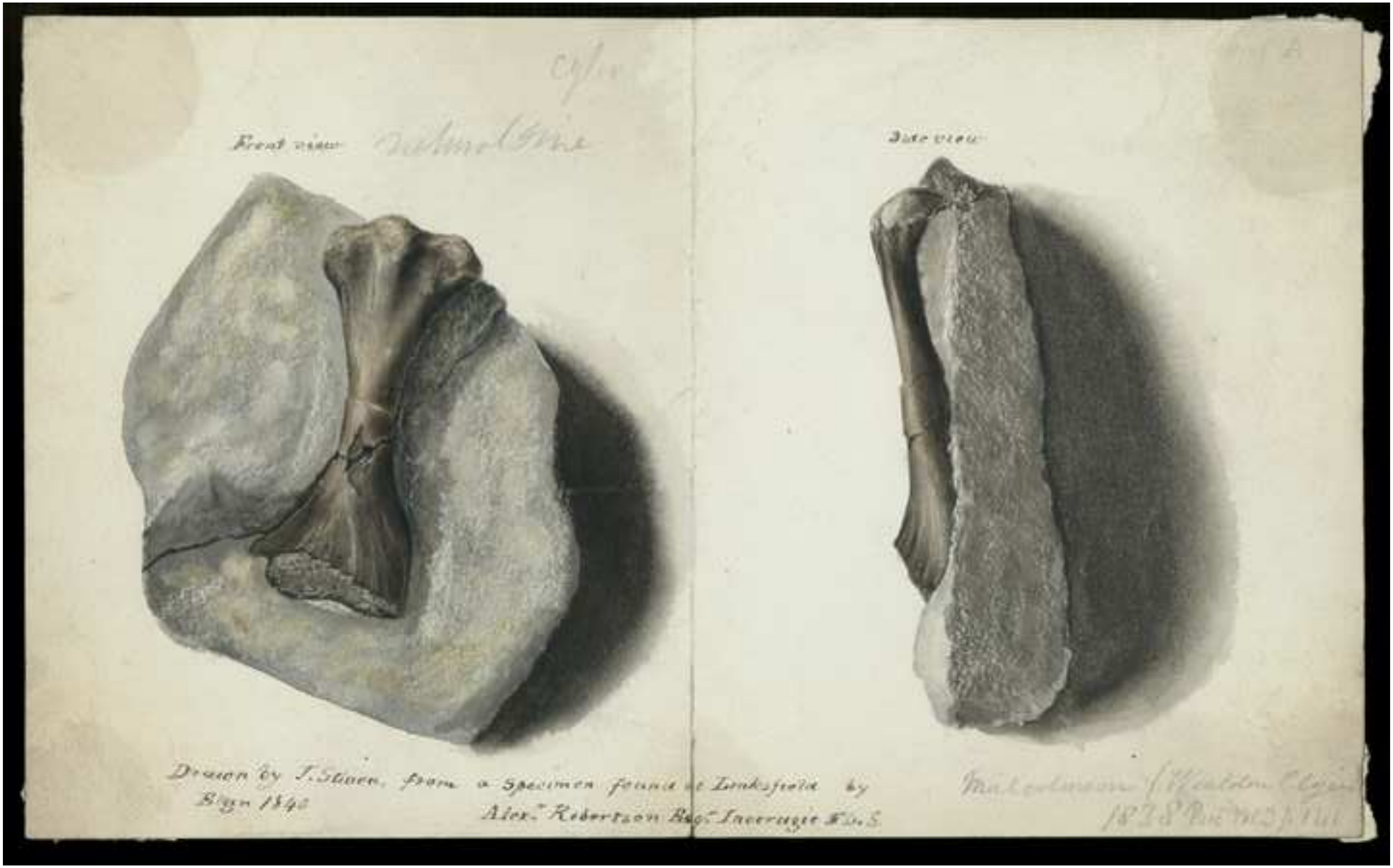

Figure 2

Click here to access/download;Figure;Fig 2.tif

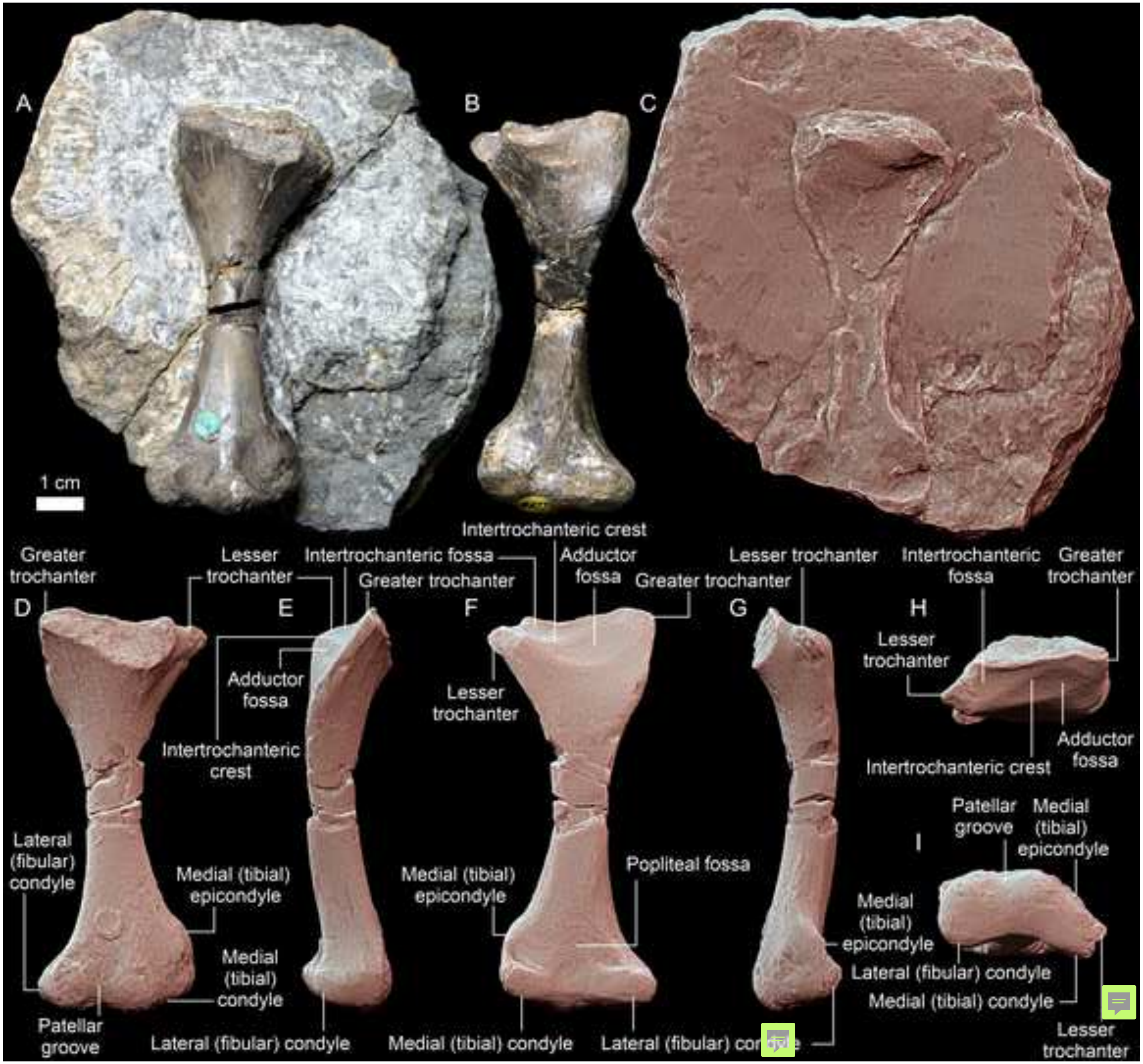

Figure 3

Click here to access/download;Figure;Fig 3 turtle humeri.tif

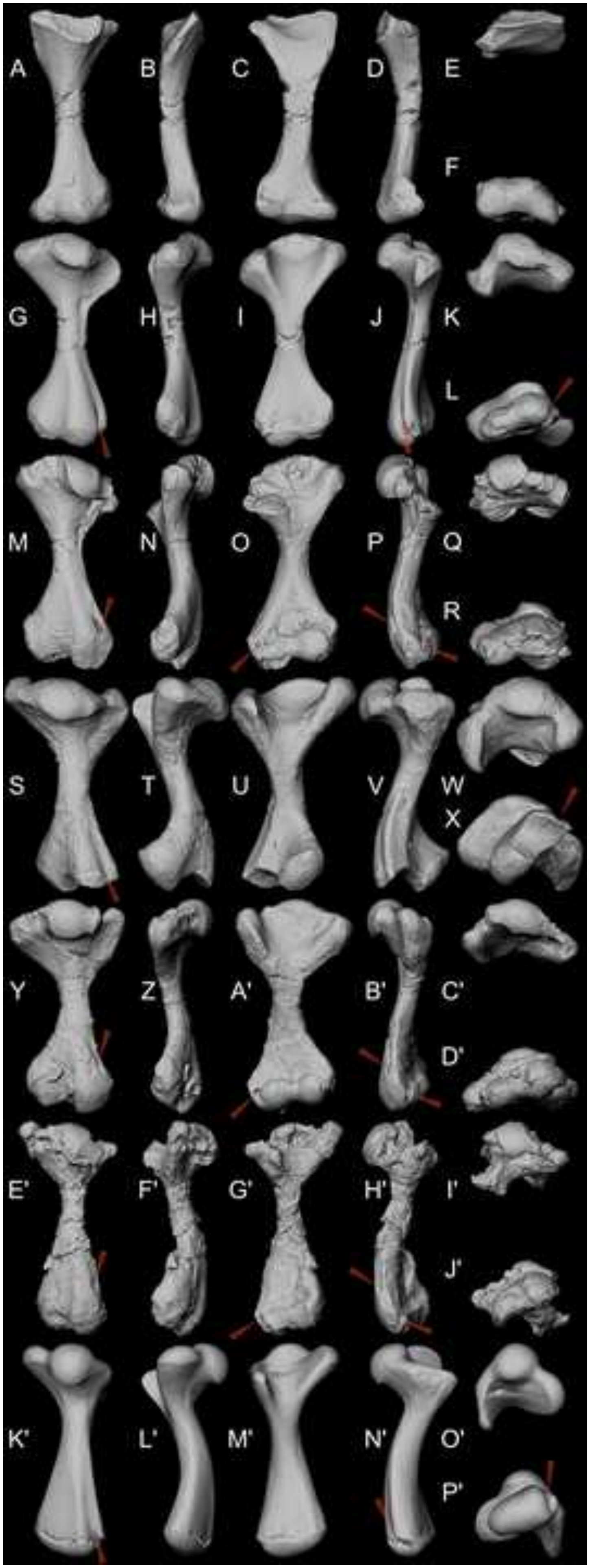

Figure 4

Click here to access/download;Figure;Fig 4 turtle femora.tif

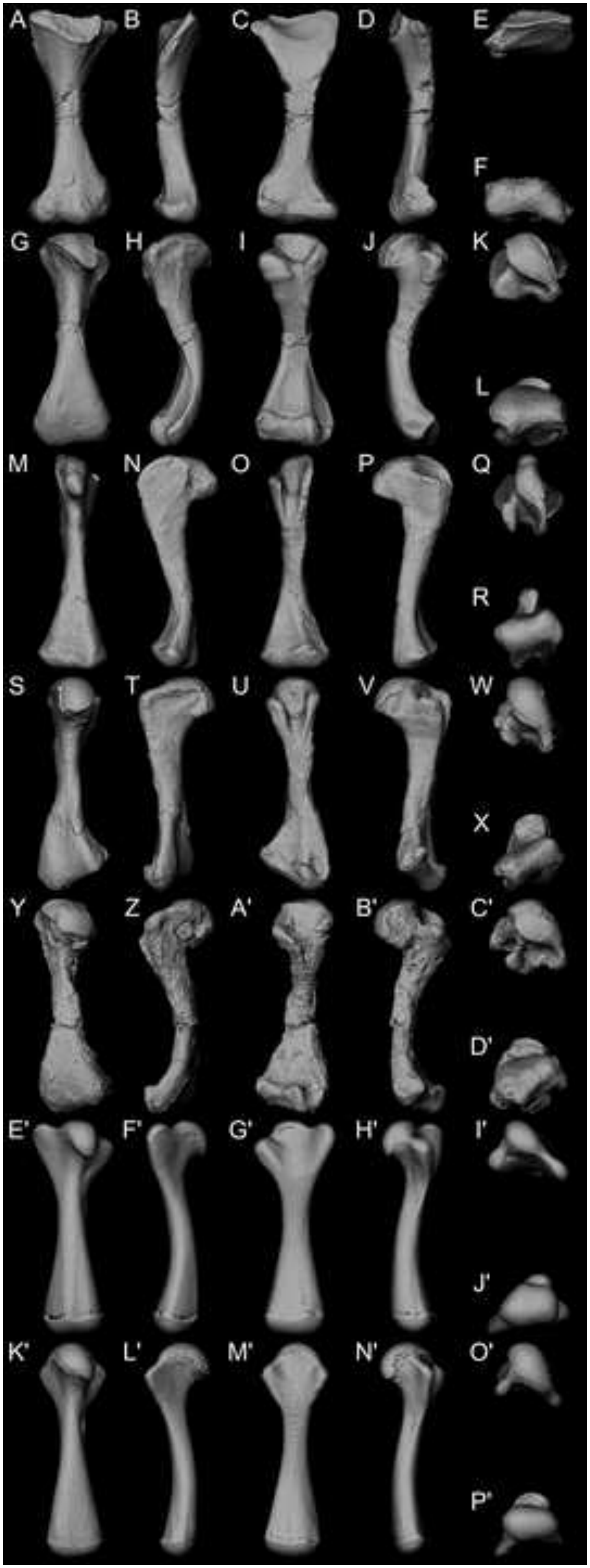

Figure 5

Click here to access/download;Figure;Fig 5 other femora.tif

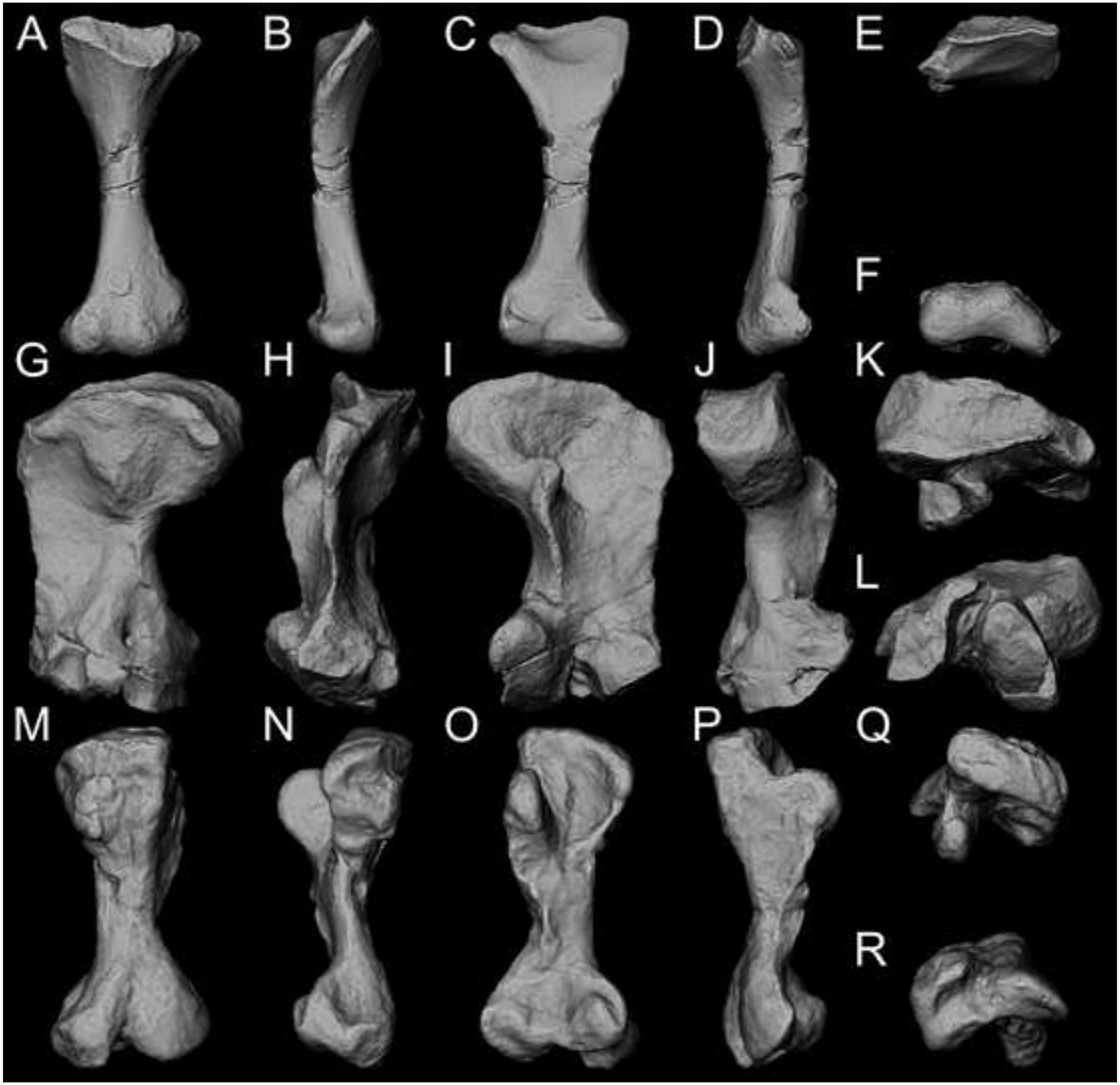

Figure 6

Click here to access/download;Figure;Fig 6 other humeri.tif

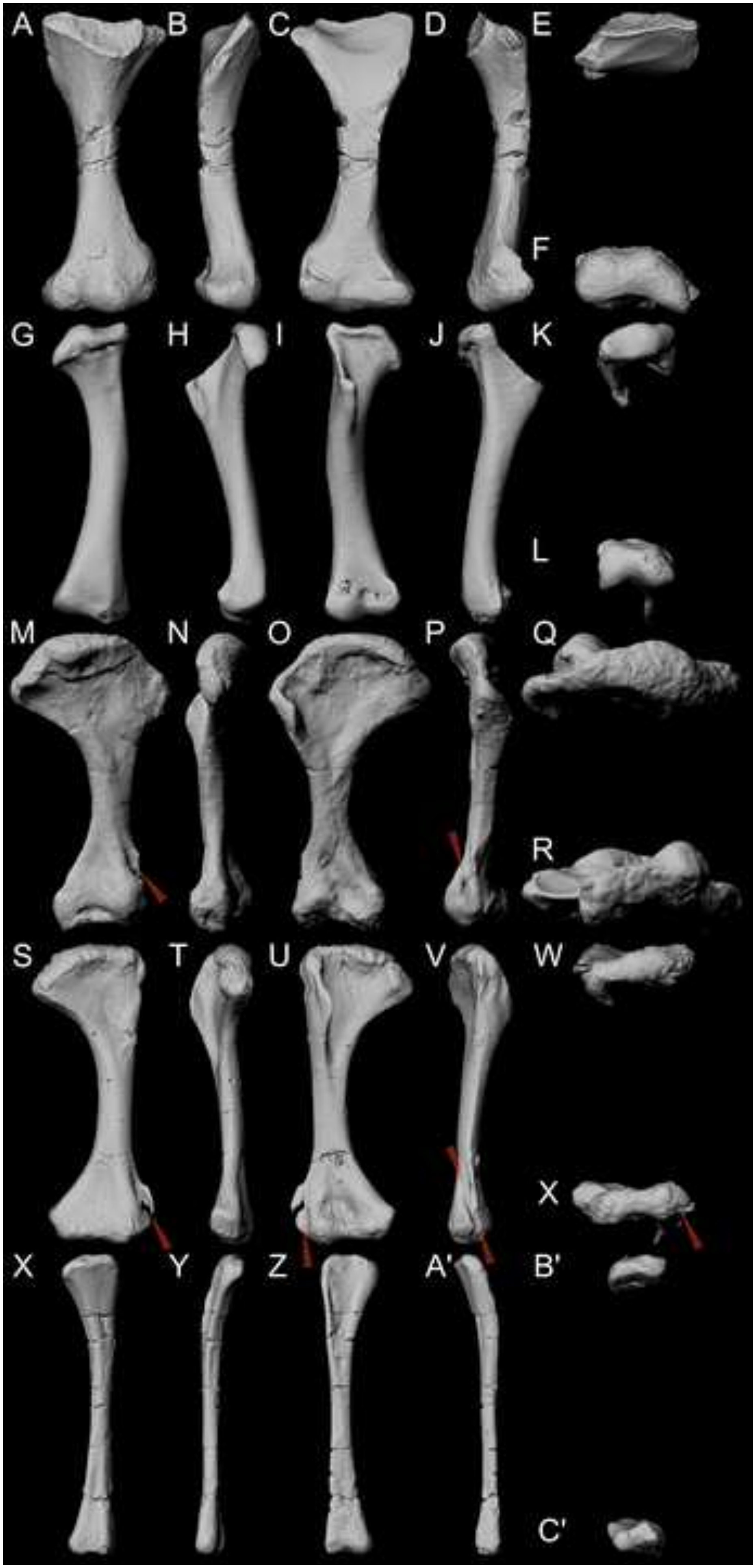

Figure 7

Click here to access/download;Figure;Fig 7 non-cynodont femora.tif

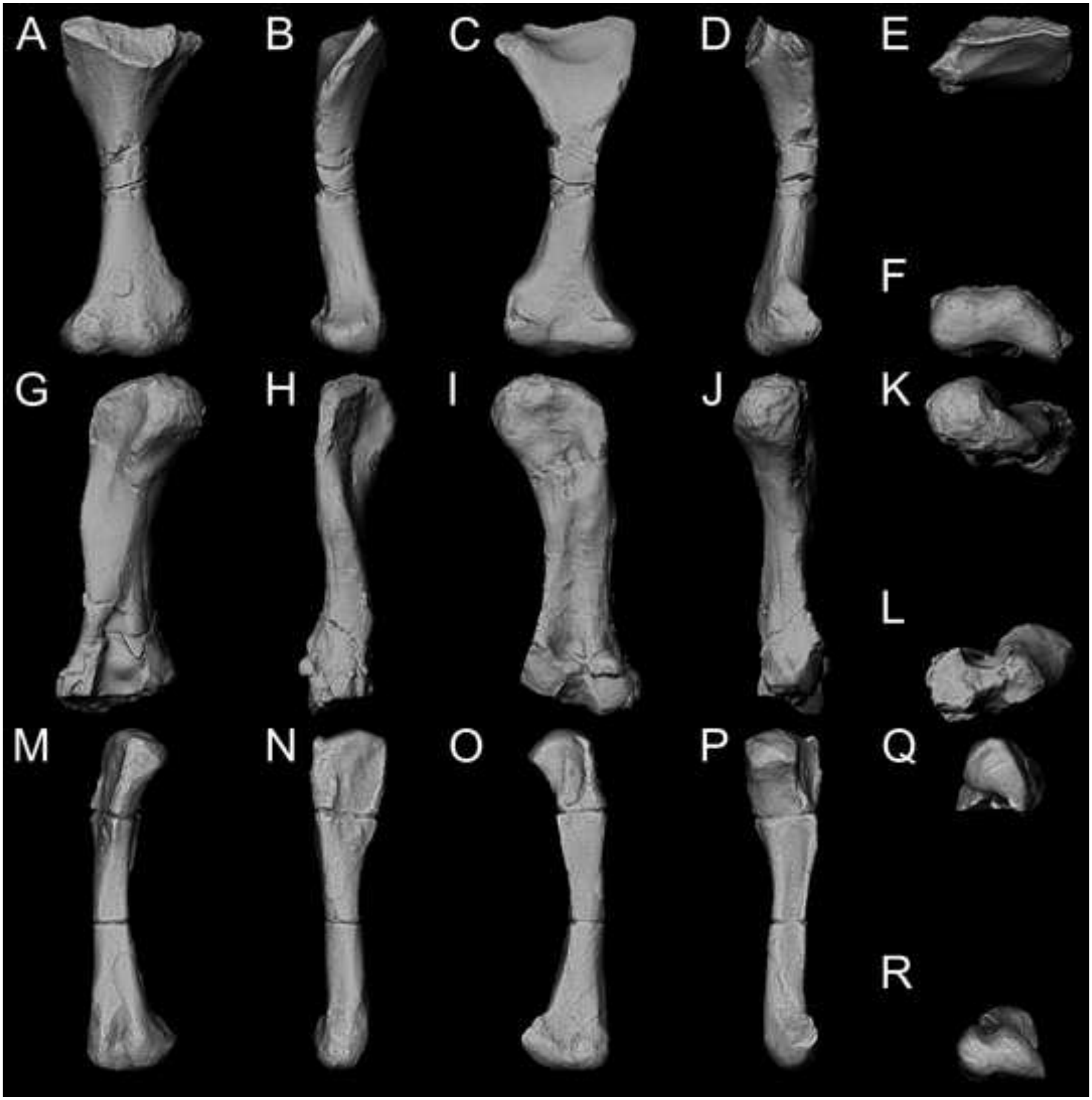

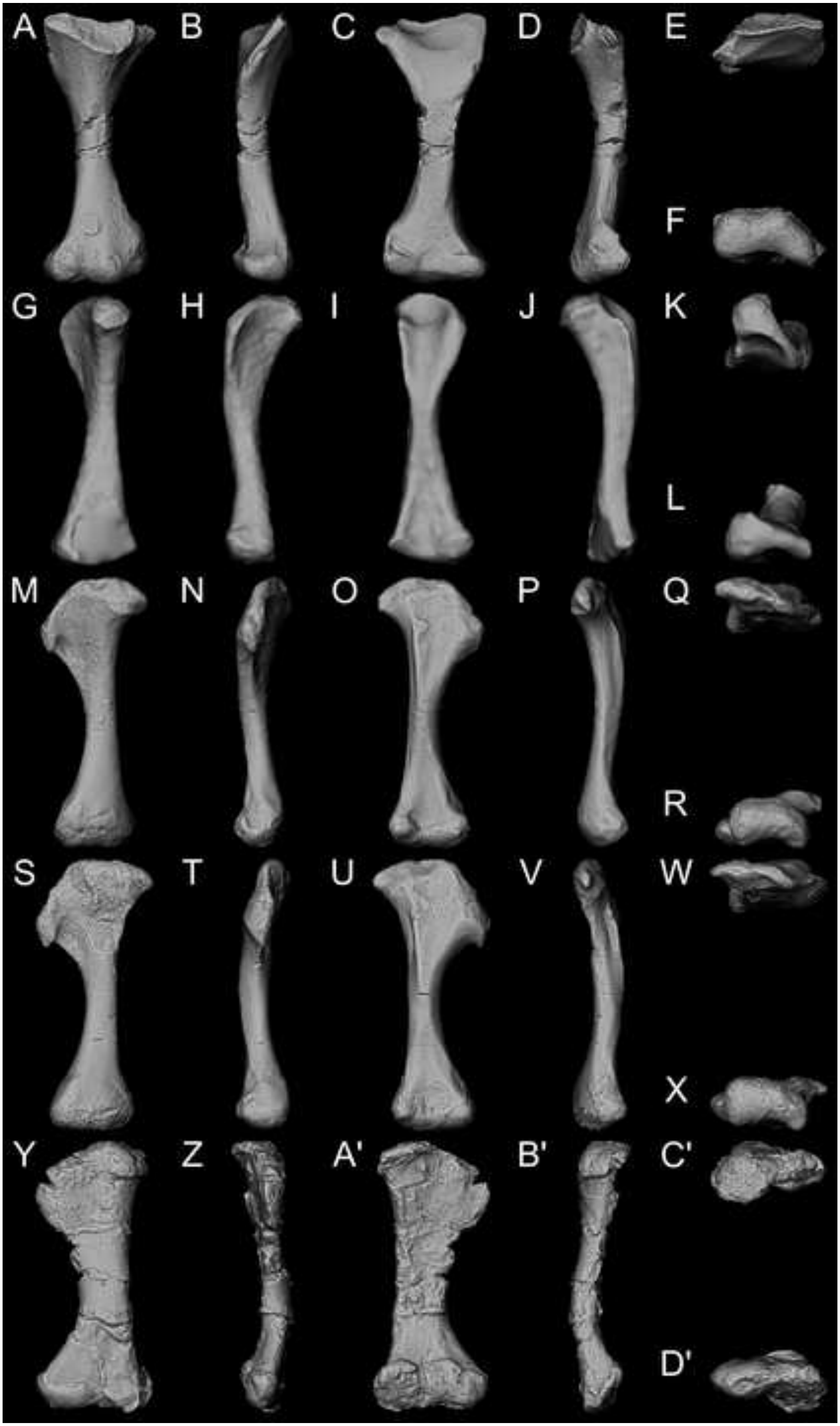
A
B
C
D
E
F
G
H
I
J
K
L
M
N
O
P
Q
R
S
T
U
V
W
X
Y
Z
A'
B'
C'
D'

Figure 9 Click here to access/download;Figure;Fig 9 cynodont femora B.tif

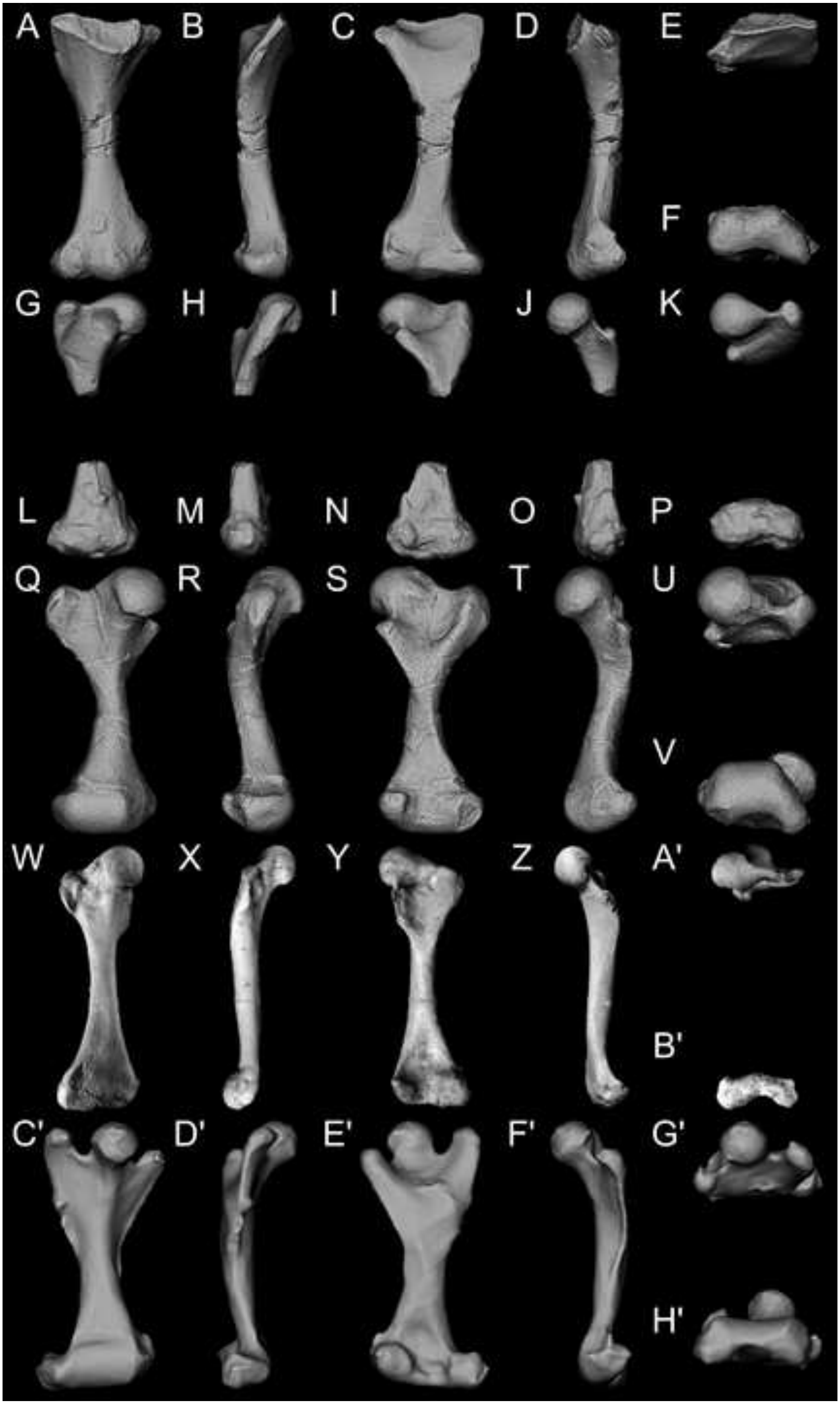

Supplement: Attachment — Submitted filename: Plos One Abdala review.pdf [file pone.0303973.s002.pdf]
