# Supplementary material for: Saurodesmus robertsoni Seeley 1891—The oldest Scottish cynodont
Source: PLoS One. 2024 May 29;19(5):e0303973. doi: 10.1371/journal.pone.0303973 (PMC11135747; doi:10.1371/journal.pone.0303973)
Supplement: Attachment — Submitted filename: Responses 2024 04 05.docx [file pone.0303973.s005.docx]

**Dear Editor, dear Reviewera,**

Thank you once again for your helpful comments and suggestions. We introduced modifications to the text and indicated them with change tracking. We furthermore improved the figures by increasing the visibility of arrowheads, adding a sharper photograph of the specimen and natural cross sections in Fig 2, and adding a femur of *Procynosuchus*.

**Yours sincerely,**

**Tomasz Szczygielski**

**Reviewer #1**

**“In the last review, I asked the synapomrphies, your fig 12 really give some and it lay the foundation for further studies. I did not read in detail in this round, but am satisfy on current structure. Your previous version give a broad comparison and could be a good reference for the study of limb bones of early tetrapods, but not a good paper itself for your purpose.”**

Thank you very much!

**Reviewer #2**

**“This is a much improved version of the manuscript and there are only a few additional comments to the authors included in the pdf.”**

Thank you very much for all the corrections!

**Figure 3: “not sure if it is for the resolution of the manuscript figure, but this should be of better final quality as it is very difficult to read.”**

Yes, this is the fault of review PDF resolution, the image is indeed very compressed but it is much clearer in the original.

**Figure 4: “use a sharper red color. It is barely visible in the figure.”**

The red is now more striking.

**Figure 7: “I did not see in the manuscript that you compare Stagonolepis, Parasuchus and Silesaurus morphologies with that of Saurodesmus. If they are compared in the supplementary material (I did not check!) you should then include these images in that section.”, “Make more visible the arrows.”**

We do not refer to those taxa specifically, but they are shown as representatives of the groups discussed in the text: parasuchians (we added a more direct reference to phytosaurs and aetosaurs in line 610 and it is discussed more in lines 625–634) and dinosauromorphs (line 658).

**Table 1: “Langbergia modisei?”**

The femur of *Langbergia modisei* was not published, we produced a 3D model from photographs of the original specimen taken by one of us (M.v.d.B.). To make it clear, we also listed numbers of all relevant specimens examined for this work, either personally or from unpublished photographs or 3D models.

**Lines 337–340: “This is confuse with the "not only" I keep expecting what else (besides incompletness and odd morphology but the third elements that should be lack of knowledge in vertebrate morphology is somewhat altered in the reasoning.**

**In addition why you will say odd morphology if it was compared with several taxa?.”**

The intended meaning here is clearer when read together with the subsequent sentences. What is meant here is that correct identification of *Saurodesmus robertsoni* was hampered by its peculiar morphology and incompleteness, but also by the ongoing dynamic development of vertebrate paleontology. This caused difficulties in proper understanding of relationships of various taxa, their anatomy, anatomical diversity and taxonomy, and made some past referrals lose meaning due to the abandonment of some taxa or outdated definitions.

The fact that *Saurodesmus robertsoni* was compared to various taxa does not preclude it from being considered odd. Various researchers tried to fit the most similar bones available to them at the time but virtually always differences were noted, and the result was considered uncertain.

**Line 477: “In Proganochelys they seems to be parallel.”**

Please note that we mean here the proximal view.

**Line 582: “Check this- It is confuse to me. You consider Saurodesmus to show a bipartite distal end? That is what i beleive from the figure, but here the idea I get from the writing is the opposite.”**

No, the distal end of the femur of *Saurodesmus robertsoni* and most other cynodonts does not have a typical bipartite shape, with two roughly symmetrical, rounded condyles separated by a groove. Instead, it has three eminences above the condyles, visible in dorsal view, as explained in the text and indicated in Fig 11.

**Line 768: “Then why to add this here? You are saying that there is no similarity whatsoever between this bone and that of Saurodesmus and nobody found there were similarieties before. Then I don´t follow why to include all this comparison here.”**

We added this sentence to clarify and rule out such a possibility for the readers who may not be particularly familiar with the morphology of the crocodilian line archosaurs. We feel it is justified due to the history of assigning *Saurodesmus robertsoni* to crocodylians and their relatives.

**Lines 799–800: “Check writing, the sentence is not close and it is confuse, because it gives me the impression that you are discussing them in relation to Saurodesmus (which does not have osteoderms).”**

This part explains the difference in views on the interrelationships and taxonomy of crocodile-line archosaurs and phytosaurs during Huene’s time, which now obscures somewhat the original meaning of Huene’s attribution. However, we fixed the sentence by removing the unnecessary “at” in the middle.

**Line 866: “Not sure what do you mean with "form factor"**

We mean the general shape and proportions of the bone.

**Line 943: “any quote here? How do you know it is morganucodontid?”**

To avoid repeating the same references over and over again and muddling the text, we grouped all of them in Table 1. The fact that the Peski specimen is a morganucodontid was established by Gambaryan and Averianov (2001, ref. 118).
